# Phylogeny of the supertribe Nebriitae (Coleoptera, Carabidae) based on analyses of DNA sequence data

**DOI:** 10.3897/zookeys.1044.62245

**Published:** 2021-06-16

**Authors:** David H. Kavanaugh, David R. Maddison, W. Brian Simison, Sean D. Schoville, Joachim Schmidt, Arnaud Faille, Wendy Moore, James M. Pflug, Sophie L. Archambeault, Tinya Hoang, Jei-Ying Chen

**Affiliations:** 1 Department of Entomology, California Academy of Sciences, 55 Music Concourse Drive, San Francisco, CA 94118, USA; 2 Department of Integrative Biology, Oregon State University, Corvallis, OR 97331, USA; 3 Center for Comparative Genomics, California Academy of Sciences, 55 Music Concourse Drive, San Francisco, CA 94118, USA; 4 Department of Entomology, University of Wisconsin, Madison, WI 53706, USA; 5 Institute of Biosciences, University of Rostock, Universitätsplatz 2, D-18055 Rostock, Germany; 6 Department of Entomology, Coleoptera, Stuttgart State Museum of Natural History, Rosenstein 1, 70191 Stuttgart, Germany; 7 Department of Entomology, University of Arizona, Tucson, AZ 85721-0036, USA; 8 University of California, Berkeley, 142 Weill Hall #3200, Berkeley, CA 94720, USA; 9 University of California, Santa Cruz, Long Marine Lab, 117 McAllister Way, Santa Cruz, CA 95060, USA

**Keywords:** DNA, evolutionary tree, ground beetles, molecular phylogenetics, nomenclature, systematics, taxonomy

## Abstract

The phylogeny of the carabid beetle supertribe Nebriitae is inferred from analyses of DNA sequence data from eight gene fragments including one nuclear ribosomal gene (28S), four nuclear-protein coding genes (CAD, topoisomerase 1, PEPCK, and *wingless*), and three mitochondrial gene fragments (16S + tRNA-Leu + ND1, COI (“barcode” region) and COI (“Pat/Jer” region)). Our taxon sample included 264 exemplars representing 241 species and subspecies (25% of the known nebriite fauna), 39 of 41 currently accepted genera and subgenera (all except *Notiokasis* and *Archileistobrius*), and eight outgroup taxa. Separate maximum likelihood (ML) analyses of individual genes, combined ML analyses of nuclear, nuclear protein-coding, and mitochondrial genes, and combined ML and Bayesian analyses of the eight-gene-fragment matrix resulted in a well-resolved phylogeny of the supertribe, with most nodes in the tree strongly supported. Within Nebriitae, 167 internal nodes of the tree (out of the maximum possible 255) are supported by maximum-likelihood bootstrap values of 90% or more. The tribes Notiophilini, Opisthiini, Pelophilini, and Nebriini are well supported as monophyletic but relationships among these are not well resolved. *Nippononebria* is a distinct genus more closely related to *Leistus* than *Nebria*. *Archastes*, *Oreonebria*, *Spelaeonebria*, and *Eurynebria*, previously treated as distinct genera by some authors, are all nested within a monophyletic genus *Nebria.* Within *Nebria*, four major clades are recognized: (1) the *Oreonebria* Series, including eight subgenera arrayed in two subgeneric complexes (the *Eonebria* and *Oreonebria* Complexes); (2) the Nebriola Series, including only subgenus Nebriola; (3) the *Nebria* Series, including ten subgenera arrayed in two subgeneric complexes, the *Boreonebria* and *Nebria* Complexes, with the latter further subdivided into three subgeneric subcomplexes (the *Nebria*, *Epinebriola*, and *Eunebria* Subcomplexes)); and (4) the *Catonebria* Series, including seven subgenera arrayed in two subgeneric complexes (the *Reductonebria* and *Catonebria* Complexes). A strong concordance of biogeography with the inferred phylogeny is noted and some evident vicariance patterns are highlighted. A revised classification, mainly within the Nebriini, is proposed to reflect the inferred phylogeny. Three genus-group taxa (*Nippononebria*, *Vancouveria* and *Archastes*) are given revised status and seven are recognized as new synonymies (*Nebriorites* Jeannel, 1941 and *Marggia* Huber, 2014 = *Oreonebria* Daniel, 1903; *Pseudonebriola* Ledoux & Roux, 1989 = *Boreonebria* Jeannel, 1937; *Patrobonebria* Bänninger, 1923, *Paranebria* Jeannel, 1937 and *Barbonebriola* Huber & Schmidt, 2017 = *Epinebriola* Daniel & Daniel, 1904; and *Asionebria* Shilenkov, 1982 = *Psilonebria* Andrewes, 1923). Six new subgenera are proposed and described for newly recognized clades: Parepinebriola Kavanaugh subgen. nov. (type species: *Nebria delicata* Huber & Schmidt, 2017), Insulanebria Kavanaugh subgen. nov. (type species: *Nebria carbonaria* Eschscholtz, 1829), Erwinebria Kavanaugh subgen. nov. (type species *Nebria sahlbergii* Fischer von Waldheim, 1828), *Nivalonebria* Kavanaugh **subgen. nov.** (type species: *Nebria paradisi* Darlington, 1931), *Neaptenonebria* Kavanaugh **subgen. nov.** (type species: *Nebria ovipennis* LeConte, 1878), and *Palaptenonebria* Kavanaugh **subgen. nov.** (type species: *Nebria mellyi* Gebler, 1847). Future efforts to better understand relationships within the supertribe should aim to expand the taxon sampling of DNA sequence data, particularly within subgenera *Leistus* and *Evanoleist*us of genus *Leistus* and the *Nebria* Complex of genus *Nebria*.

## Introduction

**Figure 1.**
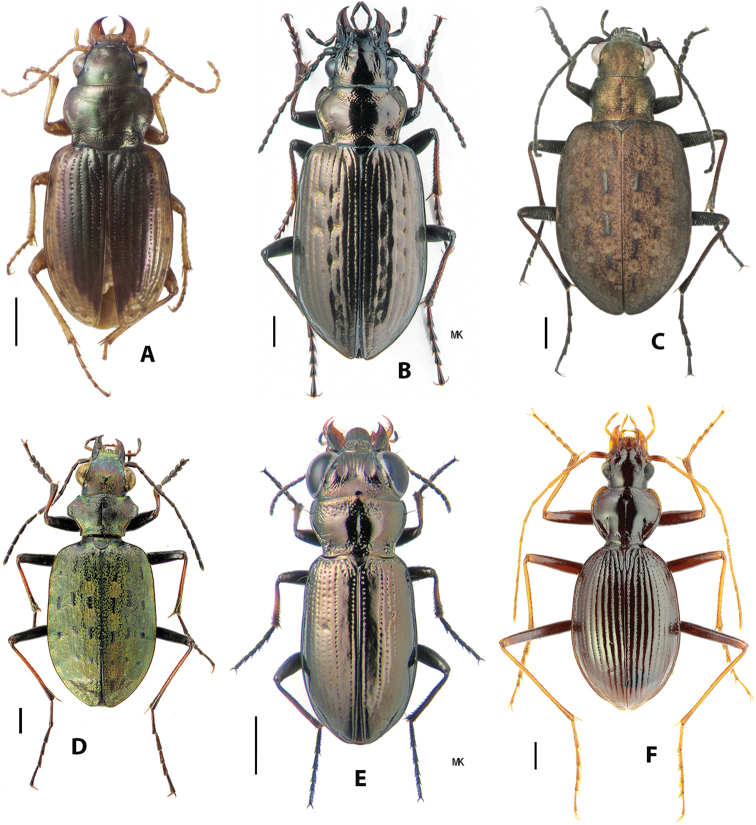
Habitus images of Nebriitae**A***Notiokasis chaudoiri* Kavanaugh & Nègre **B***Pelophila borealis* (Paykull) **C***Opisthius richardsoni* Kirby **D***Paropisthius indicus chinensis* Bousquet & Smetana **E***Notiophilus palustris* Duftschmid **F***Archileistobrius hwangtienyuni* Shilenkov & Kryzhanovskij. Scale bars: 1.0 mm. Photograph credits: **A, C** David Maddison; **B, E** Kiril Makarov; **D, F** Alexander Anischenko.

**Figure 2.**
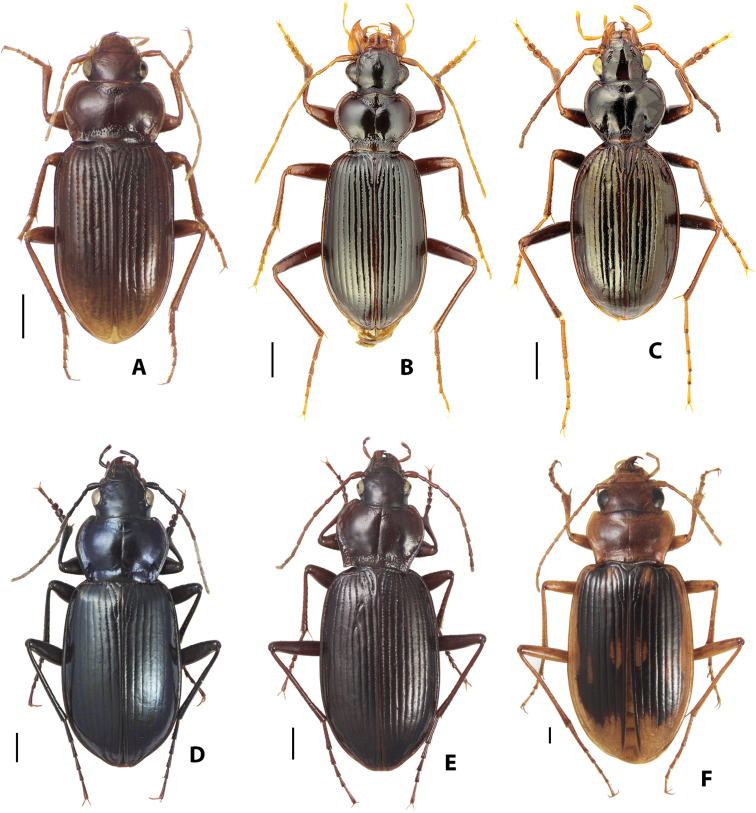
Habitus images of Nebriini**A**Leistus (Nebrileistus) nubivagus Wollaston **B**L. (Leistus) ferruginosus Mannerheim **C***Archastes solitarius* (Ledoux & Roux) **D**Nippononebria (Vancouveria) virescens (Horn) **E**Nebria (Oreonebria) castanea Bonelli **F**N. (Eurynebria) complanata (Linnaeus). Scale bars: 1.0 mm. Photograph credits: **A, D–F** David Maddison; **B, C** Alexander Anischenko.

**Figure 3.**
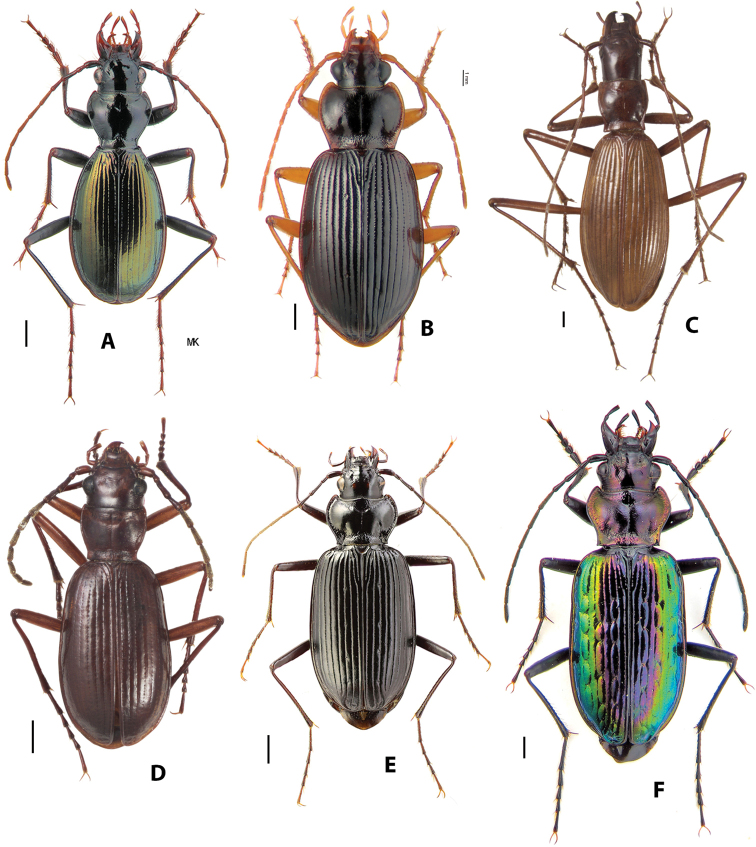
Habitus images of *Nebria***A**N. (Eonebria) djakonovi Semenov & Znojko **B**N. (Orientonebria) coreica Solsky **C**N. (Spelaeonebria) nudicollis Peyerimhoff **D**N. (Psilonebria) superna Andrewes **E**N. (Reductonebria) ochotica Sahlberg **F**N. (Catonebria) banksii Crotch. Scale bars: 1.0 mm. Photograph credits: **A, B, F** Kiril Makarov; **C, D** David Maddison; **E** Alexander Anischenko.

The carabid beetle supertribe Nebriitae is a moderately diverse group comprised of small to medium-sized beetles, varied in form (see Figs 1–3), but all sharing a suite of morphological features most of which are plesiomorphic among carabids. Most recognizable of these features include mandibles with a scrobal seta present, procoxal cavities open and confluent, mesocoxal cavities disjunct and confluent, metacoxal cavities conjunct and confluent, and protibiae with a simple, sulcate antennal cleaner (Grades “A” and “B” of Hlavac 1971) and both tibial spurs inserted apically or nearly so. At present, the supertribe includes nearly 1,100 described species and subspecies assigned to five tribes. These include the Notiophilini, Notiokasiini, Opisthiini, Pelophilini, and Nebriini.

Among these, Notiokasiini is the least diverse, represented by a single known species (Fig. 1A) in the genus *Notiokasis* Kavanaugh & Nègre, 1983, and most geographically divergent, known only from two localities in the Neotropical Region, one in Brazil and one in Uruguay. Virtually nothing is known about the habitat or life history of members of this group, and, as far as we are aware, no specimens have been collected in at least 60 years.

Pelophilini includes only two described species, both in genus *Pelophila* Dejean, 1821 (Fig. 1B), one with a Holarctic distribution, the other apparently strictly Nearctic. Both species live in boreal and subarctic areas, typically in moist to wet habitats along the margins of streams, lakes and marshes.

Opisthiini is comprised of six described species-group taxa, with one species in the Nearctic genus *Opisthius* Kirby, 1837 (Fig. 1C) and four species and one additional subspecies in the Palearctic genus *Paropisthius* Casey, 1920 (Fig. 1D). Adults and larvae of *Opisthius* are found on the bare or sparsely vegetated and exposed banks of medium-sized to large streams with soft clay or sandy soil and at least some rocky cover for hiding. *Paropisthius* adults are found under stones and other cover in open disturbed habitats, such as road-cuts, trail margins, and landslides at middle to high elevations. Adults of both genera are active mainly at night but occasionally can be found active during daylight hours.

Notiophilini includes ca. 60 described species and six additional subspecies, all in genus *Notiophilus* Duméril, 1806 (Fig. 1E). Members of all species share a body form that is unique among Carabidae and easily recognizable. Two species are Holarctic in distribution, 15 species (two of which have been introduced from Europe) occur in the Nearctic Region, two species are found in the mountainous northern part of the Neotropical Region in Middle America, and the remaining species occur in the Palearctic Region. Members of this group occur mainly in open exposed habitats or those with thin forest cover, often on gravel substrate. They prefer drier conditions than almost all other nebriites and are active in daytime, even in direct sunlight, as well as at night.

Nebriini is the most diverse tribe of the supertribe with ca. 1,000 described species and subspecies, and additional new species are discovered, mainly in Asia, almost every year. The tribe is Holarctic in distribution, with more than 90% of the described species-group taxa and most of the genus-group taxa found in the Palearctic Region. The number of extant genera currently recognized as distinct ranges from four to seven, depending on the authors. At present, the widely accepted genera include *Leistus* Frölich, 1799, *Nebria* Latreille, 1802, *Archastes* Jedlička, 1935, and *Archileistobrius* Shilenkov & Kryzhanovskij, 1983. Lorenz (2005) and Huber (2017) considered *Eurynebria* Ganglbauer, 1891b, *Oreonebria* Daniel, 1903, and *Nippononebria* Uéno, 1955 as distinct genera rather than as subgenera of *Nebria* as ranked by Ledoux and Roux (2005). Lorenz treated *Archileistobrius* as a junior synonym of *Archastes*, but Huber (2017) followed Ledoux and Roux (2009) in maintaining these as separate genera. Habu (1958), Kavanaugh (1995, 1996) and Bousquet (2012) have also treated *Nippononebria* as a distinct genus.

*Archileistobrius* (Fig. 1F) includes a single described species, *A. hwangtienyuni* Shilenkov & Kryzhanovskij, 1983, known only from a single mountain, Emei Shan, in Sichuan Province, China.

Genus *Leistus* (Fig. 2A, B) currently includes ca. 250 species and 25 additional subspecies arranged among six subgenera. The genus is Holarctic in distribution but only four species occur in the Nearctic Region and one of these has been introduced from Europe, so the genus is predominantly Palearctic. All species share a distinctive suite of modifications to the mouthparts, the ventral face of the head, and the setae and their insertion points associated with these structures that together form a basket thought to function in small prey (e.g., springtails) capture (see Bauer 1985). Different *Leistus* species occupy a broad elevational range, occurring in moist woodlands at various elevations and also above treeline at the edges of alpine streams and persistent snowfields.

*Archastes* (Fig. 2C) includes 37 described species and an additional five subspecies. Its known geographical range is confined to southcentral China, including only Sichuan, Gansu, Shaanxi, and Ningxia provinces, mainly in mountain ranges along the dissected southern edge of the Tibetan Plateau. Species occur in both forested areas and alpine steppes and talus slopes.

*Nebria* (Figs 2D–3F) is by far the most diverse genus within the tribe Nebriini with nearly 500 species and more than 100 additional subspecies described. Of these, 82 species occur in the Nearctic Region, including one introduced from Europe, and the remainder can be found in the Palearctic and northern Indomalayan regions. Members of the genus occupy cool to cold habitats from sea level to more than 5500 m in elevation, including sea beaches, the open or shaded shores of streams and lakes, the edges of persistent snowfields and glaciers, alpine talus slopes and fellfields, and even alpine caves. This diversity is arrayed among 27 currently accepted subgenera (see Table 1). As noted above, three of these have been considered separate genera by some authors. If treated as a separate genus, *Nippononebria* (Fig. 2D) includes nine described species and one additional subspecies in two subgenera: (1) the nominate subgenus, comprised of six species and one subspecies and known only from Japan and Jilin Province on the Chinese mainland; and (2) subgenus Vancouveria Kavanaugh, 1995, comprised of three described species and restricted to western North America. Members of this group range from moist lowland forests to the margins of alpine streams, seeps, and snowfields at and above treeline. *Oreonebria* (Fig. 2E) includes 14 described species and 13 additional subspecies, all restricted to the Alps mountain system of Europe and all with reduced hindwings. *Eurynebria* (Fig. 2F) includes a single species, *E. complanata* (Linnaeus), which, at least historically, inhabited sandy sea beach habitat along the Atlantic and Mediterranean coasts of southern Europe (from the southern British Isles to Turkey), and northern Africa (at least from Morocco to Tunisia).

**Table 1.** Genus-group names in supertribe Nebriitae. All published genus-group names are represented in this list in order of publication and include author, year of publication, type species and current taxonomic status (Ledoux and Roux 2005; Lorenz 2005). The “Type” column indicates if the type species for the name is represented (Y) or not (N) in our taxon sample. The “Div” column lists the number of known species for each valid genus-group name. The “Rep” column indicates how many species-group taxa are represented in our taxon sample, and the “% Rep” column indicates what percentage of the known diversity is represented in our taxon sample. The “Proposed status” column indicates the appropriate status of the names based on the results of our study, with any changes in status noted in the “Result” column.

Genus group name	Type species	Current status	Type	Div	Rep	% Rep	Proposed status	Result
*Leistus* Frölich, 1799	*Leistus testaceus* Frölich = *Carabus ferrugineus* Linnaeus	valid genus and subgenus	Y	52	11	21	valid genus and subgenus	
*Pogonophorus* Latreille, 1802	*Carabus spinibarbis* Fabricius	valid subgenus of *Leistus*	N	60	3	5	valid subgenus of *Leistus*	
*Nebria* Latreille, 1802	*Carabus brevicollis* Fabricius	valid genus and subgenus	Y	109	6	6	valid genus and subgenus	
*Manticora* Panzer, 1803	*Manticora pallipes* Panzer = *Leistus spinibarbis* Fabricius	junior homonym of *Manticora*Fabricius 1792	N	–	–	–	junior homonym of *Manticora*Fabricius 1792	
*Notiophilus* Duméril, 1805	*Cicindela aquatica* Linnaeus	valid genus	N	66	4	6	valid genus	
*Alpaeus* Bonelli, 1810	*Carabus hellwigii* Panzer	subjective junior synonym of *Nebria* s. str.	N	–	–	–	subjective junior synonym of *Nebria* s. str.	
*Pelophila* Dejean, 1821	*Carabus borealis* Paykull	valid genus	Y	2	2	100	valid genus	
*Helobia* Stephens, 1828	*Carabus brevicollis* Fabricius	objective junior synonym of *Nebria* s. str.	Y	–	–	–	objective junior synonym of *Nebria* s. str.	
*Eonebria* Semenov & Znojko, 1828	*Nebria komarovi* Semenov & Znojko	valid subgenus of *Nebria*	Y	77	6	7	valid subgenus of *Nebria*	
*Opisthius* Kirby, 1837	*Opisthius richardsoni* Kirby	valid genus	Y	1	1	100	valid genus	
*Harpazobia* Gistel, 1856	*Carabus brevicollis* Fabricius	objective junior synonym of *Nebria* s. str.	Y	–	–	–	objective junior synonym of *Nebria* s. str.	
*Eurynebria* Ganglbauer, 1891	*Carabus complanatus* Linnaeus	valid subgenus of *Nebria*	Y	1	1	100	valid subgenus of *Nebria*	
*Leistidius* Daniel, 1903	*Leistus piceus* Frölich	subjective junior synonym of *Leistus* s. str.	N	–	–	–	subjective junior synonym of *Leistus* s. str.	
*Oreonebria* Daniel, 1903	*Alpaeus castaneus* Bonelli	valid subgenus of *Nebria*	Y	26	9	35	valid subgenus of *Nebria*	
*Oreobius* Daniel, 1903	*Leistus gracilis* Fuss	subjective junior synonym of *Pogonophorus*	N	–	–	–	subjective junior synonym of *Pogonophorus*	
*Nebriola* Daniel, 1903	*Nebria laticollis* Dejean	valid subgenus of *Nebria*	Y	19	2	11	valid subgenus of *Nebria*	
*Epinebriola* Daniel & Daniel, 1904	*Nebria oxyptera* Daniel & Daniel	valid subgenus of *Nebria*	Y	28	6	21	valid subgenus of *Nebria*	
*Chaetoleistus* Semenov,1904	*Leistus relictus* Semenov	subjective junior synonym of *Pogonophorus*	N	–	–	–	subjective junior synonym of *Pogonophorus*	
*Acroleistus* Reitter, 1905	*Leistus denticollis* Reitter	subjective junior synonym of *Leistus* s. str.	N	–	–	–	subjective junior synonym of *Leistus* s. str.	
*Euleistulus* Reitter, 1905	*Leistus ellipticus* Reitter = *Leistus fulvus* Chaudoir	subjective junior synonym of *Leistus* s. str.	Y	–	–	–	subjective junior synonym of *Leistus* s. str.	
*Leistophorus* Reitter, 1905	*Leistus fulvibarbis* Dejean	subjective junior synonym of *Leistus* s. str.	Y	–	–	–	subjective junior synonym of *Leistus* s. str.	
*Spelaeonebria* Peyerimhoff, 1911	*Spelaeonebria nudicollis* Peyerimhoff	valid subgenus of *Nebria*	Y	2	1	50	valid subgenus of *Nebria*	
*Eurinoleistus* Breit, 1914	*Leistus depressus* Breit	subjective junior synonym of *Pogonophorus*	N	–	–	–	subjective junior synonym of *Pogonophorus*	
*Paropisthius* Casey, 1920	*Opisthius indicus* Chaudoir	valid genus	Y	5	4	80	valid genus	
*Patrobonebria* Bänninger, 1923	*Nebria desgodinsi* Oberthür	valid subgenus of *Nebria*	Y	11	4	36	subjective junior synonym of *Epinebriola*	New Synonymy
*Psilonebria* Andrewes, 1923	*Nebria superna* Andrewes	valid subgenus of *Nebria*	Y	4	2	50	valid subgenus of *Nebria*	
*Nebrileistus* Bänninger, 1925	*Leistus ellipticus* Wollaston	valid subgenus of *Leistus*	N	2	1	50	valid subgenus of *Leistus*	
*Archastes* Jedlička, 1935	*Archastes sterbai* Jedlička	valid genus	N	42	1	2	valid subgenus of *Nebria*	New Status
*Boreonebria* Jeannel, 1937	*Carabus rufescens* Ström = *Carabus gyllenhali* Schönherr	valid subgenus of *Nebria*	Y	42	19	45	valid subgenus of *Nebria*	
*Eunebria* Jeannel, 1937	*Carabus psammodes* Rossi	valid subgenus of *Nebria*	Y	54	15	28	valid subgenus of *Nebria*	
*Paranebria* Jeannel, 1937	*Carabus lividus* Linnaeus	valid subgenus of *Nebria*	Y	3	2	67	subjective junior synonym of *Epinebriola*	New Synonymy
*Neonebria* Hatch, 1939	none designated	nomen nudum	–	–	–	–	nomen nudum	
*Nebriorites* Jeannel, 1941	*Alpaeus gagates* Bonelli	valid subgenus of *Nebria*	Y	2	1	50	valid subgenus of *Nebria*	
*Alpaeonebria* Csiki, 1946	*Nebria fuscipes* Fuss	valid subgenus of *Nebria*	N	32	1	3	subjective junior synonym of *Nebria* s. str.	New Synonymy
*Nippononebria* Uéno, 1955	*Nebria pusilla* Uéno	valid subgenus of *Nebria*	N	7	2	29	valid genus and subgenus	New Status
*Agonoamara* Jedlička, 1962	*Agonoamara chujoi* Jedlička = *Nebria chalceola* Bates	subjective junior synonym of *Nippononebria*	Y	–	–	–	subjective junior synonym of *Nippononebria* s. str.	
*Evanoleistus* Jedlička, 1965	*Leistus nepalensis* Jedlička	valid subgenus of *Leistus*	N	154	9	5	valid subgenus of *Leistus*	
*Neoleistus* Erwin, 1970	*Leistus ferruginosus* Mannerheim	valid subgenus of *Leistus*	Y	3	3	100	subjective junior synonym of *Leistus* s. str.	New Synonymy
*Germaria* Jeanne, 1972	*Nebria bremii* Germar	junior homonym of *Germaria* Robineau-Desvoidy, 1830	Y	–	–	–	junior homonym of *Germaria* Robineau-Desvoidy, 1830	
*Catonebria* Shilenkov, 1975	*Carabus nitidulus* Fabricius = *Nebria banksii* Crotch	valid subgenus of *Nebria*	Y	42	39	93	valid subgenus of *Nebria*	
*Orientonebria* Shilenkov, 1975	*Nebria coreica* Solsky	valid subgenus of *Nebria*	Y	1	1	100	valid subgenus of *Nebria*	
*Reductonebria* Shilenkov, 1975	*Nebria ochotica* Sahlberg	valid subgenus of *Nebria*	Y	17	15	83	valid subgenus of *Nebria*	
*Sardoleistus* Perrault, 1980	*Leistus sardous* Baudi di Selve	valid subgenus of *Leistus*	Y	1	1	100	valid subgenus of *Leistus*	
*Tetungonebria* Shilenkov, 1982	*Nebria tetungi* Shilenkov	subjective junior synonym of *Eunebria*	N	–	–	–	incertae sedis	
*Asionebria* Shilenkov, 1982	*Nebria roborowskii* Semenov	valid subgenus of *Nebria*	Y	9	3	33	subjective junior synonym of *Psilonebria*	New Synonymy
*Notiokasis* Kavanaugh & Nègre, 1983	*Notiokasis chaudoiri* Kavanaugh & Nčgre	valid genus	N	1	0	0	valid genus	
*Archileistobrius* Shilenkov & Kryzhanovskij, 1983	*Archileistobrius hwangtienyuni* Shilenkov & Kryzhanovskij	valid genus	N	1	0	0	incertae sedis	
*Germarina* Jeanne, 1985	*Nebria bremii* Germar	junior homonym of *Germarina* Mesnil 1963	Y	–	–	–	junior homonym of *Germarina* Mesnil 1963	
*Himalayonebria* Ledoux, 1985	*Nebria nouristanensis* Ledoux	subjective junior synonym of *Epinebriola*	N	–	–	–	incertae sedis	
*Pseudonebriola* Ledoux & Roux, 1989	*Nebria saurica* Shilenkov	valid subgenus of *Nebria*	N	18	7	39	subjective junior synonym of *Boreonebria*	New Synonymy
*Latviaphilus* Barsevskis, 1994	*Elaphrus biguttatus* Fabricius	subjective junior synonym of *Notiophilus*	N	–	–	–	subjective junior synonym of *Notiophilus*	
*Makarovius* Barsevskis, 1994	*Notiophilus rufipes* Curtis	subjective junior synonym of *Notiophilus*	N	–	–	–	subjective junior synonym of *Notiophilus*	
*Sphodronebria* Sciaky & Pavesi, 1994	*Nebria paradoxa* Sciaky & Pavesi = *Nebria tetungi* Shilenkov	objective junior synonym of *Tetungonebria*	N	–	–	–	objective junior synonym of *Tetungonebria*	
*Vancouveria* Kavanaugh, 1995	*Nebria virescens* Horn	valid subgenus of *Nebria*	Y	3	3	100	valid subgenus of *Nippononebria*	
*Ledouxnebria* Deuve, 1998	*Ledouxnebria brisaci* Deuve	valid genus (fossil only)	–	1	–	–	valid genus (fossil only)	
*Epispadias* Ledoux & Roux, 1999	*Nebria janschneideri* Ledoux & Roux	valid subgenus of *Nebria*	N	2	1	50	valid subgenus of *Nebria*	
*Falcinebria* Ledoux & Roux, 2005	*Nebria reflexa* Bates	valid subgenus of *Nebria*	Y	16	2	13	valid subgenus of *Nebria*	
*Nakanebria* Ledoux & Roux, 2005	*Nebria kurosawai* Nakane	valid subgenus of *Nebria*	N	6	1	17	valid subgenus of *Nebria*	
*Sadonebria* Ledoux & Roux, 2005	*Nebria sadona* Bates	valid subgenus of *Nebria*	N	17	4	24	valid subgenus of *Nebria*	
*Tyrrhenia* Ledoux & Roux, 2005	*Carabus rubicundus* Quesnel	valid subgenus of *Nebria*	Y	18	2	11	valid subgenus of *Nebria*	
*Marggia* Huber, 2014	*Nebria bremii* Germar	valid subgenus of *Nebria*	Y	2	1	50	subjective junior synonym of *Oreonebria*	New Synonymy
*Barbonebriola* Huber & Schmidt, 2017	*Nebria barbata* Andrewes	valid subgenus of *Nebria*	N	6	1	17	subjective junior synonym of *Epinebriola*	New Synonymy
*Archaeonebria* Kavanaugh & Schmidt, 2019	*Archaeonebria inexspectata* Schmidt & Kavanaugh	valid genus (fossil only)	–	1	–	–	valid genus (fossil only)	
Parepinebriola subgen. nov.	*Nebria delicata* Huber & Schmidt	part of *Epinebriola Daniel*	Y	5	5	?	new subgenus	New Subgenus
Insulanebria subgen nov.	*Nebria snowi* Bates	part of *Reductonebria* Shilenkov	Y	2	2	100	new subgenus	New Subgenus
Erwinebria subgen. nov.	*Nebria sahlbergii* Fisher von Waldheim	part of *Reductonebria* Shilenkov	Y	21	20	95	new subgenus	New Subgenus
Nivalonebria subgen. nov.	*Nebria paradisi* Darlington	part of *Nakanebria* Ledoux & Roux	Y	2	2	100	new subgenus	New Subgenus
Neaptenonebria subgen. nov.	*Nebria ovipennis* LeConte	part of *Catonebria* Shilenkov	Y	6	6	100	new subgenus	New Subgenus
Palaptenonebria subgen. nov.	*Nebria mellyi* Gebler	part of *Catonebria* Shilenkov	Y	14	11	79	new subgenus	New Subgenus

Two additional nebriine genera have been described from fossil remains. *Ledouxnebria*Deuve (1998) was described from diatomite deposits in the Massif Central of France and dated to the Upper Miocene (10 to 5 million years ago). *Archaeonebria* Kavanaugh and Schmidt (in Schmidt et al. 2019) was described from Baltic amber and dated to the Eocene (50–35 Mya). The latter appears to be a stem lineage of the *Nippononebria* clade (Schmidt et al. 2019), but the affinities of *Ledouxnebria* are unclear.

The taxonomic history of nebriite carabids began with Linnaeus, who, in the Tenth Edition of his *Systema Naturae* (Linnaeus 1758), described one species in his genus *Cicindela* (*C. aquaticus*) and two in his genus *Carabus* (*C. ferruginosus* and *C. lividus*). Later, Linnaeus (1767) added a fourth species destined to be included among nebriites: *Carabus complanatus*. Three of these four would later be designated as the type species of different nebriite genera. All four of them occurred in Europe or North Africa, where new species continue to be discovered (e.g., Farkač et al. 2010; Szallies and Huber 2014). Description of endemic Nearctic nebriite species began with *Elaphrus aeneus*Herbst (1806) (now included in *Notiophilus*), *Nebria pallipes*Say (1823), *Leistus ferrugineus*Dejean (1831) (= *L. ferruginosus* Mannerheim, 1843, not *L. ferrugineus* (Linnaeus, 1758)), *Opisthius richardsoni*Kirby (1837), and *Nebria rudis*LeConte (1863) (now included in *Pelophila*). The Nearctic fauna is now reasonably well-known, but, again, with occasional additions (e.g., Kavanaugh and Schoville 2009, Kavanaugh 2015). Knowledge of the nebriite fauna of Asia was slower and generally later to develop, and the fauna of that vast and largely inaccessible region is still poorly known. Early descriptions for each genus from Asia included *Nebria carbonaria* Eschscholtz, 1829, *Leistus niger* Gebler, 1847, *Opisthius indicus* Chaudoir, 1863 (now included in *Paropisthius*), *Archastes sterbai*Jedlička (1935), and *Archileistobrius hwangtienyuni* Shilenkov & Kryzhanovskij, 1983. As shown graphically by Ledoux and Roux (2005:35), the accumulation curve for described species and subspecies of *Nebria* has still not begun to level off. The same is true for *Leistus* and *Archastes* species, with the main increases for each due to the discovery of new taxa from previously unsampled parts of Asia. Just a few examples of recent major contributions to the knowledge of this fauna include Deuve (2009, 2010, 2011), Dudko (2006), Dudko and Shilenkov (2001), Huber and Schmidt (2007, 2017, 2018) and Ledoux and Roux (2005, 2009). The last nebriite fauna to be described was that of the Neotropical Region, which currently includes only three species: two *Notiophilus* species (*N. specularis* Bates, 1881 and *N. chihuahuae* Casey, 1920) and *Notiokasis chaudoiri* Kavanaugh & Nègre, 1983.

As the number of described species increased rapidly in the 19^th^ and 20^th^ centuries and it became clear that groups of more or less similar species could be recognized, the description of nebriite genera and subgenera began, first through extracting species from genus *Carabus* of Linnaeus and then adding new genus-group taxa to accommodate species unknown to Linnaeus as needed. The first of these described was genus *Leistus* Frölich, 1799, followed by *Nebria* Latreille, 1802 and *Notiophilus* Duméril, 1805, and each of these included Linnaean species. Subsequently, *Pelophila* Dejean, 1821, *Opisthius* Kirby, 1837, *Eurynebria* Ganglbauer, 1891b, *Paropisthius* Casey, 1920, *Archastes* Jedlička, 1935, *Nippononebria* Uéno, 1955, *Notiokasis* Kavanaugh & Nègre, 1983, and *Archileistobrius* Shilenkov & Kryzhanovskij, 1983 were described. For three of these genera, one or more additional subgenera have been proposed. Barševskis (1994) proposed two new subgenera for *Notiophilus*, but these have not been widely accepted and are currently considered as junior synonyms of *Notiophilus*. A total of 13 additional subgenera has been proposed for *Leistus*; but in an excellent series of papers published from 1979 through 1994, Perrault treated the genus worldwide and recognized only six subgenera, including *Leistus* s. str. (Perrault 1980, 1991). His subgeneric classification is the currently accepted standard (Lorenz 2005). In total, 37 additional genus-group names have been proposed for *Nebria* in the broad sense. Of these, Ledoux and Roux (2005) recognized 27 as valid subgenera, the rest as junior synonyms or junior homonyms and one as a *nomen nudum*. As noted above, three of these have been considered as distinct genera by some authors, but only two of them (*Nebria* and *Eurynebria*) were originally described as distinct genera. See Table 1 for a list of all published nebriite genus-group names and their current status.

The suprageneric classification of nebriites has been relatively stable during the last century and a half compared with that of many other carabid groups, although the ranking and inclusiveness of various taxa has changed somewhat through time. The post-Linnaean era of carabid classification, which Ball (1979) has called the “Latreillean Period”, began with Latreille (1802), when he introduced the family name, Carabidae. He grouped the two nebriite genera known to him (*Pogonophorus* and *Nebria*, both newly described in that work) in his “Celerigrades” (equivalent to a subfamily rank) and then in his “Barbus” (equivalent to a tribal rank). Also included in “Barbus” were *Loricera* Fabricius and *Omophron* (also newly described in the same work). Laporte de Castelnau (1834) was the first to use the names Nébriites and Nebriidae. His concept of the group was broader than at present, including *Notiophilus*, *Pelophila*, *Leistus* and *Nebria*, the nebriite genera known at that time, but also *Metrius* Eschscholtz, *Omophron* Latreille, *Elaphrus* Fabricius, *Blethisa* Bonelli, *Notiobia* Perty, and even *Pteroloma* Dejean (now included in the polyphagan family Agyrtidae). Motschulsky (1850) considered *Notiophilus* as a separate tribe and Dupuis (1912) did the same for *Opisthius*, whereas Horn (1881) included both of these genera in his “Nebriini”. For Thomson (1859), Ganglbauer (1891a), Reitter (1908), Ball (1960) and Lindroth (1961), among others, Nebriini included only *Pelophila*, *Leistus*, and *Nebria* (although Ganglbauer also treated *Eurynebria* as a distinct genus within Nebriini). Kavanaugh and Nègre (1983) introduced the tribe Notiokasiini; and Kavanaugh (1996) proposed that *Pelophila* should be recognized as a tribe separate from the Nebriini. The group as whole and as presently comprised has been ranked as a single tribe, the Nebriini (Horn 1881), a supertribe, the Nebriitae (Kryzhanovskij 1976; Erwin 1985), a subfamily, the Nebriinae (Laporte de Castelnau 1834; Ball and Bousquet 2001; Lorenz 2005; Bousquet 2012; Löbl and Löbl 2017), and as a family, the Nebriidae (Jeannel 1937; Ledoux and Roux 2005). In urging use of the supertribe as a rank between tribe and subfamily, Kryzhanovskij (1978) argued against the excessive proliferation of subfamilies and for a more fine-grained hierarchic classification for groups of closely related tribes, especially within the main subfamilies, Carabinae and Harpalinae. We follow Kryzhanovskij’s suggestion for ranking in this presentation.

Few studies have explored phylogenetic relationships among the nebriite taxa through formal analyses and most of them have used morphological data. Kavanaugh (1978) used purely “manual” methods (i.e., without the aid of computer algorithms) proposed by Hennig (1966) to analyze phylogenetic relationships among the Nearctic *Nebria* and closely related Palearctic species. He mainly used the “outgroup comparison method” (Hennig 1966; see also Watrous and Wheeler 1981) and parsimony on a character-by-character basis to establish the polarity of transformation series of character states for each of 318 morphological and life history characters. Outgroup sampling included representatives of most adephagan families recognized at that time, most carabid tribes, all nebriite genera and most subgenera, including endemic Palearctic *Nebria* subgenera (see appendix C in Kavanaugh 1978 for a list of outgroup taxa and the text for methods of tree construction and optimization). The resulting phylogenetic hypothesis suggested that the Nearctic *Nebria* fauna included representatives of only four monophyletic groups, corresponding to the subgenera *Nippononebria*, *Boreonebria* Jeannel, 1937, *Reductonebria* Shilenkov, 1975 and *Catonebria* Shilenkov, 1995, and that *Nippononebria* was sister to a group including all the other subgenera of *Nebria*. Later, Kavanaugh (1996) reported on the results of a parsimony analysis of morphological data for all nebriite genera (except *Archileistobrius*) and selected *Nebria* subgenera with a small set of outgroup taxa. The monophyly of supertribe Nebriitae was well supported in this analysis, but that of tribe Nebriini including *Pelophila* was not, so a new tribe, Pelophilini, was proposed. A clade including *Nippononebria* and *Leistus* but not *Nebria* was also supported, which served as the basis for subsequent assertion of *Nippononebria* as a genus distinct from *Nebria* (Kavanaugh 1995). However, results from a second parsimony analysis (Kavanaugh 1998), with an expanded set of outgroup taxa but a reduced set of morphological characters, found the Nebriitae to be paraphyletic and *Pelophila* to be more closely related to the nebriine genera than a clade including Notiokasiini, Notiophilini, and Opisthiini. These conflicting results from different analyses and datasets were interpreted as resulting more from homoplasy in many of the morphological characters examined than to differences in the composition of the outgroup or suites of characters included. Ledoux and Roux (2005) provided a reclassification of *Nebria* worldwide based primarily on a custom-built, distance-based clustering method, which used thresholds for defining boundaries between taxa; they created useful groupings by adjusting the values of these thresholds. They recognized clusters and isolated outliers as subgenera, a few of which were monospecific and several of which they described as new, and their classification represents a major advance in our understanding of the most diverse genus of nebriites. They too found homoplasy in morphological characters traditionally used to characterize subgenera of *Nebria* as a major impediment to phylogenetic reconstruction.

Until now, the use of molecular data for phylogenetic studies of nebriites has been very limited. Clarke et al. (2001) examined phylogenetic relationships within the *gregaria* infragroup of *Nebria* in western North America using a rapid amplified polymorphic DNA (RAPD) analysis of molecular data from five mitochondrial DNA regions. Kavanaugh et al. (2011) used morphological, morphometric, and molecular sequence data from four nuclear and two mitochondrial genes to investigate whether *Nebria lacustris*Casey (1913) represented one or more distinct species. Schoville et al. (2012), Weng et al. (2016) and Weng et al. (2020) have published phylogeographic analyses of several species of *Nebria* from high elevations in the Sierra Nevada of California and Taiwan and the western North American species of *Nippononebria*, respectively. Raupach et al. (2019) presented a first analysis of phylogenetic relationships among 15 *Notiophilus* species and their putative colonization of the continents based on sequence data from the COI barcode region of mitochondrial DNA. The phylogenetic analyses that we present here are the first for the Nebriitae as a whole based on DNA sequence data.

Working with insights gained from phylogenetic studies based on morphological data gathered during the past 50 years and guided by Perrault’s (1980, 1991) classification of *Leistus* and Ledoux and Roux’s (2005) phylogenetic classification of *Nebria* for sampling the two most diverse elements of the nebriite fauna worldwide, the lead author (DHK) began collecting material suitably preserved for DNA extraction in mid-1990’s. Co-authors Faille, Maddison, Schmidt, and Schoville collected and subsequently provided important additional samples. We stopped collecting and accepting additional materials for these analyses at the end February 2017. In this work, we provide: (1) the results of our analyses and their implications for the current nebriite phylogenetic hypothesis and classification; (2) the evidence for or against the monophyly of selected nebriite taxa at all levels in the classification; (3) proposed changes to nebriite classification based on our findings; and (4) suggestions for some next steps that would expand and improve on these research results.

## Materials and methods

### Taxon sampling

In total, 264 exemplar specimens were used in this study. These included representatives of 241 different nebriite species-group taxa plus 14 replicates and eight outgroup taxa plus one replicate (Table 2). Locality data for these specimens are provided in Appendix A. Outgroup selection was based on morphological phylogenetic studies by Kavanaugh (1978, 1996, 1998) and the molecular phylogenetic study by Maddison et al. (1999), subject to the availability of material suitable for extraction. Nebriite taxa included in the study represent ca. 25% of the known species-group taxa and 95% (39 out of 41) of the currently accepted genus-group taxa for the supertribe. For the purposes of this study, taxa currently ranked as species and subspecies are all treated as distinct species here and lumped together as “species-group taxa” in most statements about the diversity of groups. From the Nearctic fauna, sampling included four (27%) of 15 *Notiophilus* species, the single *Opisthius* species, both *Pelophila* species, all four *Leistus* species, all three *Nippononebria* species, 84 (98%) of the 86 described *Nebria* species, and all eight genus-group taxa represented. Sampling from the Palearctic fauna was much less comprehensive, but with 35 (97%) of the 36 genus-group taxa represented. The sample included none of the Palearctic *Notiophilus* species, four (80%) of five of the *Paropisthius* species, the single *Pelophila* species, 24 (9%) of 259 *Leistus* species, two (29%) of seven *Nippononebria* species, only one (2%) of 42 *Archastes* species and 125 (ca. 25%) of approximately 500 described *Nebria* species-group taxa. The only currently accepted nebriite genera not represented in our sample were *Notiokasis*Kavanaugh and Nègre (1983) and *Archileistobrius*Shilenkov and Kryzhanovskij (1983). We tried to include the type species for each genus-group name in our sample and were successful for 26 (63%) of the 41 currently accepted generic and subgeneric names and three (23%) of the 13 subgeneric names currently ranked as subjective junior synonyms. Table 1 lists all published nebriite genus-group names, their date of publication, type species, representation (or not) in our taxon sample, known species and subspecies diversity, current taxonomic status, and proposed new taxonomic status based on the results of this study.

**Table 2.** Sampling of Nebriitae and outgroup species. The ID column indicates how each voucher specimen was identified. A number indicates that the specimen was identified by the lead author (DK) using the corresponding numbered references as follows: (1) Lindroth (1961), (2) Andrewes (1929), (3) Bousquet and Smetana (1996), (4) Kasahara (1989), (5) Uéno (1955), (6) Perrault (1981), (8) Jeannel (1941), (9) Perrault (1985), (10) Minowa (1932), (11) Farkač (1995), (12) Perrault (1986), (13) Erwin (1970), (14) Dudko (2003), (15) Perrault (1991), (16) Huber and Schmidt (2016), (17) Ledoux and Roux (2005), (18) Shilenkov (1975), (19) Ledoux and Roux (2009), (20) Trautner (1987), (21) Huber and Schmidt (2013), (22) Dudko (2006) and (23) Sasakawa (2020). A “T” indicates that the specimen was compared with the primary type specimen. A two-letter code indicates that the specimen was identified by one of the following: AF = A. Faille; DM = D.R. Maddison; JS = J. Schmidt; and RD = R.Y. Dudko. Three-letter codes indicate the following: CRC = compared with material in Philippe Roux collection and matching no taxon therein; and NID = could not be identified to species. In the remaining columns, GenBank numbers (MW359101 through MW361062) are provided for each specimen from which the listed gene sequence was successfully recovered in this study. Additional information on these voucher specimens is provided in Appendix A. Other GenBank numbers are for previously published sequences from Kavanaugh et al. (2011), Maddison (2008), Maddison et al. (2009), Ober (2002), and Wild and Maddison (2008). Sequences of the five specimens marked with an asterisk in the ID column are paratypes and the sequences are thus “genseq-2”, with the remainder being “genseq-4” (Chakrabarty et al. 2013).

	ID	28S	16S-ND1	CO1 BC	CO1 PJ	CAD2	PEPCK	Topo	*wg*
**Supertribe Nebriitae**									
**Tribe Notiophilini**									
**Genus *Notiophilus* Duméril**									
*Notiophilus borealis* Harris	1	MW359101	MW360814	MW359348	MW359611	MW360101	MW360345	MW359863	MW360574
*Notiophilus semistriatus* Say	1	MW359102	MW360815	MW359349	MW359612	MW360102	MW360346	MW359864	MW360575
*Notiophilus sierranus* Casey	1	MW359103	MW360816	MW359350	MW359613	MW360103	MW360347	MW359865	MW360576
*Notiophilus sylvaticus* Dejean	1	MW359104	MW360817	MW359351	MW359614	MW360104	MW360348	MW359866	MW360577
**Tribe Opisthiini**									
**Genus *Opisthius* Kirby**									
*Opisthius richardsoni* Kirby	1	MW359105	MW360818	MW359352	MW359615	MW360105	MW360349	MW359867	MW360578
**Genus *Paropisthius* Casey**									
*Paropisthius davidis* (Fairmaire)	2	MW359106	MW360819	MW359353	MW359616	MW360106	MW360350	MW359868	MW360579
*Paropisthius indicus chinensis* Bousquet & Smetana	3	MW359107	MW360820	MW359354	MW359617	MW360107	MW360351	MW359869	MW360580
*Paropisthius indicus indicus* (Chaudoir)	3	MW359108	MW360821	MW359355	MW359618	MW360108	MW360352	MW359870	MW360581
*Paropisthius masuzoi* Kasahara	4	MW359109	MW360822	MW359356	MW359619	MW360109	MW360353	MW359871	MW360582
**Tribe Pelophilini**									
**Genus *Pelophila* Dejean**									
*Pelophila borealis* (Paykull) AK	1	MW359110	MW360823	MW359357	MW359620	MW360110	MW360354	MW359872	MW360583
*Pelophila borealis* (Paykull) RFE	1	MW359111	–	MW359358	MW359621	MW360111	–	MW359873	MW360584
*Pelophila rudis* (LeConte)	1	MW359112	MW360824	MW359359	MW359622	MW360112	MW360355	MW359874	MW360585
**Tribe Nebriini**									
**Genus *Nippononebria* Uéno**									
**Subgenus Nippononebria Uéno**									
*Nippononebria chalceola* (Bates)	5	–	–	MN982525	MN982525	–	–	–	–
*Nippononebria changbaiensis* Kavanaugh & Liang	T	MW359113	MW360825	MW359360	MW359623	MW360113	MW360356	MW359875	MW360586
**Subgenus Vancouveria Kavanaugh**									
*Nippononebria altisierrae* Kavanaugh	T	MW359114	MW360826	MW359361	MW359624	MW360114	MW360357	MW359876	MW360587
*Nippononebria campbelli* Kavanaugh	T	MW359115	MW360827	MW359362	MW359625	MW360115	MW360358	MW359877	MW360588
*Nippononebria virescens* (Horn)	T	MW359116	MW360828	MW359363	MW359626	MW360116	MW360359	MW359878	MW360589
**Genus *Leistus* Frölich**									
**Subgenus Nebrileistus Bänninger**									
*Leistus nubivagus* Wollaston	6	MW359117	MW360829	MW359364	MW359627	MW360117	MW360360	MW359879	MW360590
**Subgenus Sardoleistus Perrault**									
*Leistus sardous* Baudi di Selve	AF	MW359118	MW360830	MW359365	MW359628	MW360118	–	–	MW360591
**Subgenus Pogonophorus Latreille**									
*Leistus parvicollis* Chaudoir	8	MW359119	MW360831	MW359366	MW359629	MW360119	MW360361	–	MW360592
*Leistus pyrenaeus* Kraatz	8	MW359120	MW360832	MW359367	MW359630	MW360120	–	MW359880	MW360593
*Leistus rufomarginatus* (Duftschmid)	8	MW359121	MW360833	MW359368	MW359631	MW360121	MW360362	MW359881	MW360594
**Subgenus Evanoleistus Jedlička**									
*Leistus birmanicus* Perrault	9	MW359122	MW360834	MW359369	MW359632	MW360122	MW360363	MW359882	MW360595
*Leistus gaoligongensis* Kavanaugh & Long	T	MW359123	MW360835	MW359370	MW359633	MW360123	MW360364	MW359883	MW360596
*Leistus lihengae* Kavanaugh & Long	T	MW359124	MW360836	MW359371	MW359634	MW360124	MW360365	MW359884	MW360597
*Leistus niitakaensis* Minowa	10	MW359125	MW360837	MW359372	MW359635	MW360125	MW360366	MW359885	MW360598
*Leistus nokoensis* Minowa	10	MW359126	MW360838	MW359373	MW359636	MW360126	MW360367	MW359886	MW360599
*Leistus smetanai* Farkač	11	MW359127	MW360839	MW359374	MW359637	MW360127	MW360368	MW359887	MW360600
*Leistus taiwanensis* Perrault	12	MW359128	MW360840	MW359375	MW359638	MW360128	MW360369	MW359888	MW360601
*Leistus* sp NEP	NID	MW359129	MW360841	MW359376	MW359639	MW360129	–	MW359889	MW360602
*Leistus* sp YUN 1	NID	MW359130	MW360842	MW359377	MW359640	MW360130	MW360370	MW359890	MW360603
**Subgenus Leistus Frölich**									
*Leistus crenatus* Fairmaire	8	MW359131	MW360843	MW359378	MW359641	MW360131	MW360371	MW359891	MW360604
*Leistus ferrugineus* (Linnaeus)	8	MW359132	MW360844	MW359379	MW359642	MW360132	MW360372	MW359892	MW360605
*Leistus ferruginosus* Mannerheim	13	MW359133	MW360845	MW359380	MW359643	MW360133	MW360373	MW359893	MW360606
*Leistus kryzhanovskii frateroides* Dudko	14	MW359134	MW360846	MW359381	MW359644	MW360134	MW360374	MW359894	MW360607
*Leistus kryzhanovskii kryzhanovskii* Dudko	14	MW359135	MW360847	MW359382	MW359645	MW360135	MW360375	MW359895	MW360608
*Leistus fulvibarbis* Dejean	8	MW359136	MW360848	MW359383	MW359646	MW360136	–	–	MW360609
*Leistus fulvus* Chaudoir	8	–	MW360849	MW359384	MW359647	–	–	–	–
*Leistus longipennis* Casey	13	MW359137	MW360850	MW359385	MW359648	MW360137	MW360376	MW359896	MW360610
*Leistus madmeridianus* Erwin	13	MW359138	MW360851	MW359386	MW359649	MW360138	MW360377	MW359897	MW360611
*Leistus niger* Gebler	15	MW359139	MW360852	MW359387	MW359650	MW360139	MW360378	MW359898	MW360612
*Leistus nitidus* (Duftschmid)	8	MW359140	MW360853	MW359388	MW359651	MW360140	MW360379	–	MW360613
*Leistus* sp JIL 2	NID	MW359141	MW360854	MW359389	MW359652	MW360141	MW360380	MW359899	MW360614
*Leistus* sp SCH	NID	MW359142	MW360855	MW359390	MW359653	MW360142	MW360381	MW359900	MW360615
*Leistus* sp YUN 2	NID	MW359143	MW360856	MW359391	MW359654	MW360143	MW360382	MW359901	MW360616
**Genus *Nebria* Latreille**									
***Oreonebria* Series**									
***Eonebria* Complex**									
**Subgenus Pseudepinebriola New Subgenus**									
*Nebria delicata* Huber & Schmidt^1^	16	MW359144	MW360857	MW359392	MW359655	MW360144	MW360383	MW359902	MW360617
*Nebria retingensis* Huber & Schmidt^1^	16*	MW359145	MW360858	MW359393	MW359656	MW360145	MW360384	MW359903	MW360618
*Nebria* sp YUN 4	NID	MW359146	MW360859	MW359394	MW359657	MW360146	MW360385	MW359904	MW360619
*Nebria* sp YUN 5	NID	MW359147	MW360860	MW359395	MW359658	MW360147	MW360386	MW359905	MW360620
*Nebria* sp XIZ 14	NID	MW359148	MW360861	MW359396	MW359659	MW360148	MW360387	MW359906	MW360621
**Subgenus Sadonebria Ledoux & Roux**									
*Nebria chinensis* Bates	17	MW359149	MW360862	MW359397	MW359660	MW360149	MW360388	MW359907	MW360622
*Nebria niitakana* Kano	17	MW359150	MW360863	MW359398	MW359661	MW360150	MW360389	MW359908	MW360623
*Nebria ohdaiensis* Nakane	17	MW359151	MW360864	MW359399	MW359662	MW360151	MW360390	MW359909	MW360624
*Nebria saeviens* Bates	17	MW359152	MW360865	MW359400	MW359663	MW360152	MW360391	MW359910	MW360625
**Subgenus Eonebria Semenov & Znojko**									
*Nebria komarovi* Semenov & Znojko	17	MW359153	MW360866	MW359401	MW359664	MW360153	–	MW359911	–
*Nebria* sp GAN	CRC	MW359154	MW360867	MW359402	MW359665	MW360154	–	MW359912	–
*Nebria* sp YUN 13	CRC	MW359155	MW360868	MW359403	MW359666	MW360155	MW360392	MW359913	MW360626
*Nebria* sp YUN 16	CRC	MW359156	MW360869	MW359404	MW359667	MW360156	MW360393	MW359914	MW360627
*Nebria* sp YUN *17*	CRC	MW359157	MW360870	MW359405	MW359668	MW360157	MW360394	MW359915	MW360628
*Nebria* sp YUN 18	CRC	MW359158	MW360871	MW359406	MW359669	MW360158	MW360395	MW359916	MW360629
***Oreonebria* Complex**									
**Subgenus*Epispadias* Ledoux & Roux**									
*Nebria* sp YUN 6	CRC	MW359159	MW360872	MW359407	MW359670	MW360159	MW360396	MW359917	MW360630
**Subgenus Falcinebria Ledoux & Roux**									
*Nebria formosana* Habu	17	MW359160	MW360873	MW359408	MW359671	MW360160	MW360397	MW359918	MW360631
*Nebria niohozana* Bates	23	MW359161	MW360874	MW359409	MW359672	MW360161	MW360398	MW359919	MW360632
**Subgenus Orientonebria Shilenkov**									
*Nebria coreica* Solsky	18	MW359162	MW360875	MW359410	MW359673	MW360162	MW360399	MW359920	MW360633
**Subgenus Archastes Jedlička**									
*Nebria yuae* (Ledoux & Roux)	19	MW359163	MW360876	MW359411	MW359674	MW360163	MW360400	MW359921	MW360634
**Subgenus Oreonebria Daniel**									
*Nebria angustata* Dejean	17	MW359164	MW360877	MW359412	MW359675	MW360164	MW360401	MW359922	MW360635
*Nebria angusticollis* Bonelli	17	MW359165	MW360878	MW359413	MW359676	MW360165	MW360402	MW359923	MW360636
*Nebria austriaca* Ganglbauer	17	MW359166	MW360879	MW359414	MW359677	MW360166	–	MW359924	MW360637
*Nebria boschi* Winkler	17	MW359167	MW360880	MW359415	MW359678	MW360167	MW360403	MW359925	MW360638
*Nebria bremii* Germar^2^	17	MW359168	MW360881	MW359416	MW359679	MW360168	MW360404	MW359926	MW360639
*Nebria castanea* Bonelli	17	MW359169	MW360882	MW359417	MW359680	MW360169	MW360405	MW359927	MW360640
*Nebria diaphana* Daniel & Daniel	17	MW359170	MW360883	MW359418	MW359681	MW360170	–	MW359928	MW360641
*Nebria gagates* (Bonelli)^3^	17	MW359171	MW360884	MW359419	MW359682	MW360171	–	MW359929	MW360642
*Nebria ligurica* Daniel	17	MW359172	MW360885	MW359420	MW359683	MW360172	–	MW359930	MW360643
*Nebria lombarda* Daniel & Daniel	17	MW359173	–	MW359421	MW359684	MW360173	–	MW359931	MW360644
*Nebria picea* Dejean	17	MW359174	MW360886	MW359422	MW359685	MW360174	MW360406	MW359932	MW360645
***Nebriola* Series**									
**Subgenus Nebriola Daniel**									
*Nebria fontinalis* Daniel & Daniel	17	MW359175	MW360887	MW359423	MW359686	MW360175	MW360407	MW359933	MW360646
*Nebria laticollis* Dejean	17	MW359176	MW360888	MW359424	MW359687	MW360176	MW360408	MW359934	MW360647
***Nebria* Series**									
***Boreonebria* Complex**									
**Subgenus Nakanebria Ledoux & Roux**									
*Nebria shiretokoana* Nakane	17	MW359177	MW360889	MW359425	MW359688	MW360177	MW360409	MW359935	MW360648
**Subgenus Boreonebria Jeannel**									
*Nebria baicalica* Motschulsky	18	JN847585	JN847610	MW359426	JN847535	JN847560	MW360410	JN847510	JN847635
*Nebria bellorum* Kavanaugh	T	JN847603	JN847628	MW359427	JN847553	JN847578	MW360411	JN847528	JN847653
*Nebria castanipes* Kirby	T	MW359178	MW360890	MW359428	MW359689	MW360178	MW360412	MW359936	MW360649
*Nebria changaica* Horvatovich	17	MW359179	MW360891	MW359429	MW359690	MW360179	–	MW359937	MW360650
*Nebria crassicornis* Van Dyke	1	JN847581	JN847606	MW359430	JN847531	JN847556	–	JN847506	JN847631
*Nebria dabanensis* Shilenkov^4^	17	MW359180	MW360892	MW359431	MW359691	MW360180	–	MW359938	MW360651
*Nebria frigida* Sahlberg	1	MW359181	MW360893	MW359432	MW359692	MW360181	MW360413	MW359939	MW360652
*Nebria gouleti* Kavanaugh	T	JN847587	JN847612	MW359433	JN847537	JN847562	MW360414	JN847512	JN847637
*Nebria gyllenhali* (Schönherr)	T	JN847582	JN847607	MW359434	JN847532	JN847557	–	JN847507	JN847632
*Nebria hudsonica* LeConte	T	JN847589	JN847614	MW359435	JN847539	JN847564	MW360415	JN847514	JN847639
*Nebria intermedia* Van Dyke OR	1	MW359182	MW360894	MW359436	MW359693	MW360182	–	MW359940	MW360653
*Nebria intermedia* Van Dyke UT	1	MW359183	MW360895	MW359437	MW359694	MW360183	–	MW359941	MW360654
*Nebria intermedia* Van Dyke WY	1	MW359184	MW360896	MW359438	MW359695	MW360184	–	MW359942	MW360655
*Nebria kaszabi* Shilenkov^4^	17	MW359185	MW360897	MW359439	MW359696	MW360185	–	MW359943	MW360656
*Nebria lacustris* Casey	T	JN847590	JN847615	MW359440	JN847540	JN847565	MW360416	JN847515	JN847640
*Nebria lassenensis* Kavanaugh	T	MW359186	MW360898	MW359441	MW359697	MW360186	MW360417	MW359944	MW360657
*Nebria lindrothi* Kavanaugh	T	MW359187	MW360899	MW359442	MW359698	MW360187	MW360418	MW359945	MW360658
*Nebria murzini* Ledoux & Roux^4^	17	MW359188	MW360900	MW359443	MW359699	MW360188	–	MW359946	MW360659
*Nebria nivalis* (Paykull) AK	1	JN847584	JN847609	MW359444	JN847534	JN847559	MW360419	JN847509	JN847634
*Nebria nivalis* (Paykull) NU	1	JN847583	JN847608	MW359445	JN847533	JN847558	MW360420	JN847508	JN847633
*Nebria nivalis* gr sp	NID	MW359189	MW360901	MW359446	MW359700	MW360189	MW360421	MW359947	MW360660
*Nebria rubrofemorata* Shilenkov	18	MW359190	MW360902	MW359447	MW359701	MW360190	MW360422	MW359948	MW360661
*Nebria sajanica* Bänninger^4^	17	MW359191	MW360903	MW359448	MW359702	MW360191	–	MW359949	MW360662
*Nebria sajanica* gr sp 1^4^	NID	MW359192	MW360904	MW359449	MW359703	–	–	–	MW360663
*Nebria sajanica* gr sp 2^4^	NID	MW359193	MW360905	MW359450	MW359704	MW360192	MW360423	MW359950	MW360664
*Nebria subdilatata* Motschulsky	18	JN847586	JN847611	MW359451	JN847536	JN847561	MW360424	JN847511	JN847636
*Nebria tekesensis* Ledoux & Roux^4^	17	MW359194	MW360906	MW359452	MW359705	MW360193	–	MW359951	MW360665
*Nebria* sp MG 1	NID	MW359195	MW360907	MW359453	MW359706	MW360194	MW360425	MW359952	MW360666
*Nebria* sp MG 2	NID	MW359196	MW360908	MW359454	MW359707	MW360195	MW360426	MW359953	MW360667
***Nebria* Complex**									
***Nebria* Subcomplex**									
**Subgenus Tyrrhenia Ledoux & Roux**									
*Nebria fulviventris* Bassi	17	MW359197	MW360909	MW359455	MW359708	MW360196	MW360427	MW359954	MW360668
*Nebria rubicunda maroccana* Antoine	17	MW359198	MW360910	MW359456	MW359709	MW360197	–	MW359955	MW360669
**Subgenus Nebria Latreille**									
*Nebria brevicollis* (Fabricius) OR	20	JN847580	MW360911	MW359457	JN847530	JN847555	MW360428	JN847505	JN847630
*Nebria brevicollis* (Fabricius) SP	20	MW359199	MW360912	MW359458	MW359710	MW360198	MW360429	MW359956	MW360670
*Nebria lafresnayi* Audinet-Serville	8	MW359200	MW360913	MW359459	MW359711	MW360199	–	MW359957	MW360671
*Nebria olivieri* Dejean	8	MW359201	MW360914	MW359460	MW359712	MW360200	MW360430	MW359958	MW360672
*Nebria rubripes* Audinet-Serville	8	MW359202	MW360915	MW359461	MW359713	MW360201	MW360431	MW359959	MW360673
*Nebria tibialis* Bonelli	17	MW359203	MW360916	MW359462	MW359714	MW360202	MW360432	MW359960	MW360674
*Nebria turcica* Chaudoir	17	MW359204	MW360917	MW359463	MW359715	MW360203	–	–	MW360675
**Subgenus Alpaeonebria Csiki**									
*Nebria germari* Heer AU	17	MW359205	MW360918	MW359464	MW359716	MW360204	MW360433	MW359961	MW360676
*Nebria germari* Heer GE	17	MW359206	MW360919	MW359465	MW359717	MW360205	MW360434	MW359962	MW360677
**Subgenus Spelaeonebria Peyerimhoff**									
*Nebria nudicollis* Peyerimhoff	AF	MW359207	MW360920	MW359466	MW359718	MW360206	–	MW359963	MW360678
***Epinebriola* Subcomplex**									
**Subgenus Epinebriola Daniel**									
*Nebria businskyorum* Ledoux & Roux	16	MW359208	MW360921	MW359467	MW359719	MW360207	MW360435	MW359964	MW360679
*Nebria capillosa* Ledoux & Roux^6^	JS	MW359209	MW360922	MW359468	MW359720	MW360208	MW360436	MW359965	–
*Nebria cf. desgodinsi* Oberthür^6^	JS	MW359210	MW360923	MW359469	MW359721	MW360209	–	MW359966	–
*Nebria kagmara* Huber & Schmidt^5^	16*	MW359211	MW360924	MW359470	MW359722	MW360210	MW360437	–	MW360680
*Nebria cf. laevistriata* Ledoux & Roux	NID	MW359212	MW360925	MW359471	MW359723	MW360211	MW360438	MW359967	MW360681
*Nebria livida* (Linnaeus)^7^	17	MW359213	MW360926	MW359472	MW359724	MW360212	MW360439	MW359968	MW360682
*Nebria macrogona* Bates^7^	17	MW359214	–	MW359473	MW359725	MW360213	MW360440	MW359969	MW360683
*Nebria martensi* Huber & Schmidt	16	MW359215	MW360927	MW359474	MW359726	MW360214	–	–	MW360684
*Nebria numburica* Huber & Schmidt	16*	MW359216	MW360928	MW359475	MW359727	MW360215	MW360441	MW359970	MW360685
*Nebria oxyptera* Daniel & Daniel	16	MW359217	MW360929	MW359476	MW359728	–	–	–	–
*Nebria pertinax* Huber & Schmidt^6^	JS	MW359218	MW360930	MW359477	MW359729	MW360216	MW360442	MW359971	MW360686
*Nebria pseudorestias* Huber & Schmidt	16	MW359219	MW360931	MW359478	MW359730	MW360217	MW360443	MW359972	MW360687
*Nebria* sp YUN 1^6^	NID	MW359220	MW360932	MW359479	MW359731	MW360218	MW360444	MW359973	MW360688
**Subgenus Psilonebria Andrewes**									
*Nebria composita macra* Ledoux & Roux^9^	17	MW359221	MW360933	MW359480	MW359732	MW360219	MW360445	MW359974	MW360689
*Nebria mentoincisa* Huber & Schmidt^8^	21*	MW359222	MW360934	MW359481	MW359733	MW360220	MW360446	MW359975	MW360690
*Nebria nana* Ledoux & Roux^9^	17	MW359223	MW360935	MW359482	MW359734	MW360221	MW360447	MW359976	MW360691
*Nebria pharina walteriana* Ledoux & Roux^8^	17	MW359224	MW360936	MW359483	MW359735	MW360222	MW360448	MW359977	MW360692
*Nebria przewalskii* Semenov^9^	17	MW359225	MW360937	MW359484	MW359736	MW360223	MW360449	MW359978	–
*Nebria roborowskii* Semenov^8^	17	MW359226	MW360938	MW359485	MW359737	MW360224	MW360450	MW359979	MW360693
*Nebria cf. superna* Andrewes	JS	MW359227	MW360939	MW359486	MW359738	MW360225	MW360451	MW359980	MW360694
*Nebria* sp XIZ 20	NID	MW359228	MW360940	MW359487	MW359739	MW360226	MW360452	MW359981	MW360695
**Subgenus Eurynebria Ganglbauer**									
*Nebria complanata* (Linnaeus)	8	MW359229	MW360941	MW359488	MW359740	MW360227	–	MW359982	MW360696
**Subgenus Eunebria Jeannel**									
*Nebria jockischii* Sturm	17	MW359230	MW360942	MW359489	MW359741	MW360228	MW360453	MW359983	MW360697
*Nebria lewisi* Bates	17	MW359231	MW360943	MW359490	MW359742	MW360229	MW360454	MW359984	MW360698
*Nebria limbigera* Solsky	17	MW359232	MW360944	MW359491	MW359743	MW360230	MW360455	MW359985	–
*Nebria morvani* Ledoux & Roux	JS	MW359233	MW360945	MW359492	MW359744	MW360231	MW360456	MW359986	MW360699
*Nebria perlonga* Heyden	17	MW359234	MW360946	MW359493	MW359745	MW360232	MW360457	MW359987	MW360700
*Nebria picicornis* (Fabricius)	17	MW359235	MW360947	MW359494	MW359746	MW360233	MW360458	MW359988	–
*Nebria psammodes* (Rossi)	17	MW359236	MW360948	MW359495	MW359747	MW360234	MW360459	MW359989	–
*Nebria uenoiana* Habu	17	MW359237	MW360949	MW359496	MW359748	MW360235	MW360460	MW359990	MW360701
*Nebria yunnana* Bänninger	17	MW359238	MW360950	MW359497	MW359749	MW360236	MW360461	MW359991	MW360702
*Nebria* sp YUN 9	CRC	MW359239	MW360951	MW359498	MW359750	MW360237	MW360462	MW359992	MW360703
*Nebria*sp YUN 10-dark	CRC	MW359240	MW360952	MW359499	MW359751	MW360238	MW360463	MW359993	MW360704
*Nebria* sp YUN 10-pale	CRC	MW359241	MW360953	MW359500	MW359752	MW360239	MW360464	MW359994	MW360705
*Nebria* sp YUN 11	CRC	MW359242	MW360954	MW359501	MW359753	MW360240	MW360465	MW359995	MW360706
***Catonebria* Series**									
***Reductonebria* Complex**									
**Subgenus Insulanebria New Subgenus**									
*Nebria carbonaria* Eschscholtz^10^	18	MW359243	MW360955	MW359502	MW359754	MW360241	MW360466	MW359996	MW360707
*Nebria snowi* Bates^10^	18	MW359244	MW360956	MW359503	MW359755	MW360242	MW360467	MW359997	MW360708
**Subgenus Reductonebria Shilenkov**									
*Nebria altaica* Gebler	18	MW359245	MW360957	MW359504	MW359756	MW360243	MW360468	MW359998	MW360709
*Nebria appalachia* Darlington	T	MW359246	MW360958	MW359505	MW359757	MW360244	MW360469	MW359999	MW360710
*Nebria chuskae* Kavanaugh	T	MW359247	MW360959	MW359506	MW359758	MW360245	MW360470	MW360000	MW360711
*Nebria darlingtoni* Kavanaugh	T	MW359248	MW360960	MW359507	MW359759	MW360246	MW360471	MW360001	MW360712
*Nebria desolata* Kavanaugh	T	MW359249	MW360961	MW359508	MW359760	MW360247	MW360472	MW360002	MW360713
*Nebria diversa* LeConte	T	MW359250	MW360962	MW359509	MW359761	MW360248	MW360473	MW360003	MW360714
*Nebria eschscholtzii* Ménétriés	1	MW359251	MW360963	MW359510	MW359762	MW360249	MW360474	MW360004	MW360715
*Nebria georgei* Kavanaugh	T*	MW359252	MW360964	MW359511	MW359763	MW360250	MW360475	MW360005	MW360716
*Nebria japonica* Bates	17	MW359253	MW360965	MW359512	MW359764	MW360251	MW360476	MW360006	MW360717
*Nebria mannerheimii* Fischer von Waldheim	1	MW359254	MW360966	MW359513	MW359765	MW360252	MW360477	MW360007	MW360718
*Nebria navajo* Kavanaugh	T	MW359255	MW360967	MW359514	MW359766	MW360253	MW360478	MW360008	MW360719
*Nebria obliqua* LeConte CO	T	MW359256	MW360968	MW359515	MW359767	MW360254	MW360479	MW360009	MW360720
*Nebria obliqua* LeConte UT	T	MW359257	MW360969	MW359516	MW359768	MW360255	MW360480	MW360010	MW360721
*Nebria ochotica* Sahlberg	18	MW359258	MW360970	MW359517	MW359769	MW360256	MW360481	MW360011	MW360722
*Nebria pallipes* Say	1	MW359259	MW360971	MW359518	MW359770	MW360257	MW360482	MW360012	MW360723
*Nebria suturalis* LeConte AB	T	MW359260	MW360972	MW359519	MW359771	MW360258	MW360483	MW360013	MW360724
*Nebria suturalis* LeConte CO	T	MW359261	MW360973	MW359520	MW359772	MW360259	MW360484	MW360014	MW360725
*Nebria suturalis* LeConte NH	T	MW359262	MW360974	MW359521	MW359773	MW360260	MW360485	MW360015	MW360726
**Subgenus Erwinebria New Subgenus**									
*Nebria acuta* Lindroth^10^	T	MW359263	MW360975	MW359522	MW359774	MW360261	MW360486	MW360016	MW360727
*Nebria arkansana* Casey^10^	T	MW359264	MW360976	MW359523	MW359775	MW360262	MW360487	MW360017	MW360728
*Nebria charlottae* Lindroth^10^	T	MW359265	MW360977	MW359524	MW359776	MW360263	MW360488	MW360018	MW360729
*Nebria danmanni* Kavanaugh^10^	T	MW359266	MW360978	MW359525	MW359777	MW360264	MW360489	MW360019	MW360730
*Nebria edwardsi* Kavanaugh^10^	T	MW359267	MW360979	MW359526	MW359778	MW360265	MW360490	MW360020	MW360731
*Nebria fragilis* Casey^10^	T	MW359268	MW360980	MW359527	MW359779	MW360266	MW360491	MW360021	MW360732
*Nebria gregaria* Fischer von Waldeim^10^	1	MW359269	MW360981	MW359528	MW359780	MW360267	MW360492	MW360022	MW360733
*Nebria haida* Kavanaugh^10^	T	MW359270	MW360982	MW359529	MW359781	MW360268	MW360493	MW360023	MW360734
*Nebria jeffreyi* Kavanaugh^10^	T	MW359271	MW360983	MW359530	MW359782	MW360269	MW360494	MW360024	MW360735
*Nebria lituyae* Kavanaugh^10^	T	MW359272	MW360984	MW359531	MW359783	MW360270	MW360495	MW360025	MW360736
*Nebria louiseae* Kavanaugh^10^	T	MW359273	MW360985	MW359532	MW359784	MW360271	MW360496	MW360026	MW360737
*Nebria lyelli* Van Dyke^10^	T	MW359274	MW360986	MW359533	MW359785	MW360272	MW360497	MW360027	MW360738
*Nebria modoc* Kavanaugh^10^	T	MW359275	MW360987	MW359534	MW359786	MW360273	MW360498	MW360028	MW360739
*Nebria oowah* Kavanaugh^10^	T	MW359276	MW360988	MW359535	MW359787	MW360274	MW360499	MW360029	MW360740
*Nebria quileute* Kavanaugh^10^	T	MW359277	MW360989	MW359536	MW359788	MW360275	MW360500	MW360030	MW360741
*Nebria sahlbergii* Fischer von Waldheim^10^	T	MW359278	MW360990	MW359537	MW359789	MW360276	MW360501	MW360031	MW360742
*Nebria sonorae* Kavanaugh^10^	T	MW359279	MW360991	MW359538	MW359790	MW360277	MW360502	MW360032	MW360743
*Nebria triad* Kavanaugh^10^	T	MW359280	MW360992	MW359539	MW359791	MW360278	MW360503	MW360033	MW360744
*Nebria wallowae* Kavanaugh^10^	T	MW359281	MW360993	MW359540	MW359792	MW360279	MW360504	MW360034	MW360745
*Nebria zioni* Van Dyke^10^	T	MW359282	MW360994	MW359541	MW359793	MW360280	MW360505	MW360035	MW360746
***Catonebria* Complex**									
**Subgenus Nivalonebria New Subgenus**									
*Nebria paradisi* Darlington OR^11^	T	MW359283	MW360995	MW359542	MW359794	MW360281	MW360506	MW360036	MW360747
*Nebria paradisi* Darlington WA^11^	T	MW359284	MW360996	MW359543	MW359795	MW360282	MW360507	MW360037	MW360748
*Nebria turmaduodecima* Kavanaugh^11^	T	MW359285	MW360997	MW359544	MW359796	MW360283	MW360508	MW360038	MW360749
**Subgenus Neaptenonebria New Subgenus**									
*Nebria balli* Kavanaugh^12^	T	MW359286	MW360998	MW359545	MW359797	MW360284	MW360509	MW360039	MW360750
*Nebria carri* Kavanaugh^12^	T	MW359287	MW360999	MW359546	MW359798	MW360285	MW360510	MW360040	MW360751
*Nebria kincaidi* Schwarz^12^	T	MW359288	MW361000	MW359547	MW359799	MW360286	MW360511	MW360041	MW360752
*Nebria ovipennis* LeConte^12^	T	MW359289	MW361001	MW359548	MW359800	MW360287	MW360512	MW360042	MW360753
*Nebria sierrae* Kavanaugh^12^	T	MW359290	MW361002	MW359549	MW359801	MW360288	MW360513	MW360043	MW360754
*Nebria spatulata* Van Dyke^12^	T	MW359291	MW361003	MW359550	MW359802	MW360289	MW360514	MW360044	MW360755
**Subgenus Palaptenonebria New Subgenus**									
*Nebria arinae* Dudko & Shilenkov^12^	RD	MW359292	MW361004	MW359551	MW359803	MW360290	MW360515	MW360045	MW360756
*Nebria baenningeri baenningeri* Dudko & Shilenkov^12^	RD	MW359293	MW361005	MW359552	MW359804	MW360291	MW360516	MW360046	MW360757
*Nebria baenningeri korgonica* Dudko & Shilenkov^12^	RD	MW359294	MW361006	MW359553	MW359805	MW360292	MW360517	MW360047	MW360758
*Nebria lyubechanskii* Dudko^12^	RD	MW359295	MW361007	MW359554	MW359806	MW360293	MW360518	MW360048	MW360759
*Nebria mellyi mellyi* Gebler^12^	RD	MW359296	MW361008	MW359555	MW359807	MW360294	MW360519	MW360049	MW360760
*Nebria mellyi teletskiana* Dudko & Shilenkov^12^	RD	MW359298	MW361010	MW359557	MW359809	MW360296	MW360521	MW360051	MW360762
*Nebria roddi* Dudko & Shilenkov^12^	RD	MW359297	MW361009	MW359556	MW359808	MW360295	MW360520	MW360050	MW360761
*Nebria sajana dubatolovi* Dudko & Shilenkov^12^	RD	MW359299	MW361011	MW359558	MW359810	MW360297	MW360522	MW360052	MW360763
*Nebria sajana sajana* Dudko & Shilenkov^12^	RD	MW359300	MW361012	MW359559	MW359811	MW360298	MW360523	MW360053	MW360764
*Nebria sajana sarlyk* Dudko & Shilenkov^12^	RD	MW359301	MW361013	MW359560	MW359812	MW360299	MW360524	MW360054	MW360765
*Nebria sajana sitnikovi* Dudko & Shilenkov^12^	RD	MW359302	MW361014	MW359561	MW359813	MW360300	MW360525	MW360055	MW360766
**Subgenus Catonebria Shilenkov**									
*Nebria aenea* Gebler	RD	MW359303	MW361015	MW359562	MW359814	MW360301	MW360526	MW360056	MW360767
*Nebria albimontis* Kavanaugh	T	MW359304	MW361016	MW359563	MW359815	MW360302	MW360527	MW360057	MW360768
*Nebria banksii* Crotch	22	MW359305	MW361017	MW359564	MW359816	MW360303	MW360528	MW360058	MW360769
*Nebria baumanni* Kavanaugh	T	MW359306	MW361018	MW359565	MW359817	MW360304	MW360529	MW360059	MW360770
*Nebria beverlianna* Kavanaugh	T	MW359307	MW361019	MW359566	MW359818	MW360305	MW360530	MW360060	MW360771
*Nebria calva* Kavanaugh	T	MW359308	MW361020	MW359567	MW359819	MW360306	MW360531	MW360061	MW360772
*Nebria cascadensi*s Kavanaugh	T	MW359309	MW361021	MW359568	MW359820	MW360307	MW360532	MW360062	MW360773
*Nebria catenata* Casey	T	MW359310	MW360812	MW359569	MW359821	MW360308	MW360533	MW360063	MW360774
*Nebria catenulata* Fischer von Waldheim	22	MW359311	MW361022	MW359570	MW359822	MW360309	MW360534	MW360064	MW360775
*Nebria coloradensis* Van Dyke	T	MW359312	MW361023	MW359571	MW359823	MW360310	MW360535	MW360065	MW360776
*Nebria fragariae* Kavanaugh	T	MW359313	MW361024	MW359572	MW359824	MW360311	MW360536	MW360066	MW360777
*Nebria fulgida* Gebler	22	MW359314	MW361025	MW359573	MW359825	MW360312	MW360537	MW360067	MW360778
*Nebria gebleri* Dejean	T	MW359315	MW361026	MW359574	MW359826	MW360313	MW360538	MW360068	MW360779
*Nebria giulianii* Kavanaugh	T	MW359316	MW361027	MW359575	MW359827	MW360314	MW360539	MW360069	MW360780
*Nebria holzunensis* Dudko & Shilenkov	22	MW359317	MW361028	MW359576	MW359828	MW360315	MW360540	MW360070	MW360781
*Nebria ingens* Horn	T	MW359318	MW361029	MW359577	MW359829	MW360316	MW360541	MW360071	MW360782
*Nebria labontei* Kavanaugh	T	MW359319	MW361030	MW359578	MW359830	MW360317	MW360542	MW360072	MW360783
*Nebria lamarckensis* Kavanaugh	T	MW359320	MW361031	MW359579	MW359831	MW360318	MW360543	MW360073	MW360784
*Nebria meanyi* Van Dyke CA	T	MW359321	MW361032	MW359580	MW359832	MW360319	MW360544	MW360074	MW360785
*Nebria meanyi* Van Dyke WA	T	MW359322	MW361033	MW359581	MW359833	MW360320	MW360545	MW360075	MW360786
*Nebria metallica* Fischer von Waldheim	1	MW359323	MW361034	MW359582	MW359834	MW360321	MW360546	MW360076	MW360787
*Nebria pasquineli* Kavanaugh	T	MW359324	MW361035	MW359583	MW359835	MW360322	MW360547	MW360077	MW360788
*Nebria pektusanica* Horvatovich	22	MW359325	MW361036	MW359584	MW359836	MW360323	MW360548	MW360078	MW360789
*Nebria piperi* Van Dyke	T	MW359326	MW361037	MW359585	MW359837	MW360324	MW360549	MW360079	MW360790
*Nebria piute* Erwin & Ball	T	MW359327	MW360813	MW359586	MW359838	MW360325	MW360550	MW360080	MW360791
*Nebria praedicta* Kavanaugh & Schoville	T	MW359328	MW361038	MW359587	MW359839	MW360326	MW360551	MW360081	MW360792
*Nebria purpurata* LeConte CO	T	MW359329	MW361039	MW359588	MW359840	MW360327	MW360552	MW360082	MW360793
*Nebria purpurata* LeConte NM	T	MW359330	MW361040	MW359589	MW359841	MW360328	MW360553	MW360083	MW360794
*Nebria rathvoni* LeConte	T	MW359331	MW361041	MW359590	MW359842	MW360329	MW360554	MW360084	MW360795
*Nebria riversi* Van Dyke	T	MW359332	MW361042	MW359591	MW359843	MW360330	MW360555	MW360085	MW360796
*Nebria schwarzi* Van Dyke	T	MW359333	MW361043	MW359592	MW359844	MW360331	MW360556	MW360086	MW360797
*Nebria sevieri* Kavanaugh	T	MW359334	MW361044	MW359593	MW359845	MW360332	MW360557	MW360087	MW360798
*Nebria sierrablancae* Kavanaugh	T	MW359335	MW361045	MW359594	MW359846	MW360333	MW360558	MW360088	MW360799
*Nebria siskiyouensis* Kavanaugh	T	MW359336	MW361046	MW359595	MW359847	MW360334	MW360559	MW360089	MW360800
*Nebria splendida* Fischer von Waldheim	22	MW359337	MW361047	MW359596	MW359848	MW360335	MW360560	MW360090	MW360801
*Nebria steensensis* Kavanaugh	T	MW359338	MW361048	MW359597	MW359849	MW360336	MW360561	MW360091	MW360802
*Nebria sylvatica* Kavanaugh	T	MW359339	MW361049	MW359598	MW359850	MW360337	MW360562	MW360092	MW360803
*Nebria trifaria* LeConte	T	MW359340	MW361050	MW359599	MW359851	MW360338	MW360563	MW360093	MW360804
*Nebria utahensis* Kavanaugh	T	MW359341	MW361051	MW359600	MW359852	MW360339	MW360564	MW360094	MW360805
*Nebria vandykei* Bänninger	1	MW359342	MW361052	MW359601	MW359853	MW360340	MW360565	MW360095	MW360806
*Nebria wyeast* Kavanaugh	T	MW359343	MW361053	MW359602	MW359854	MW360341	MW360566	MW360096	MW360807
**Outgroup taxa**									
** Trachypachidae **									
*Trachypachus inermis* Motschulsky	1	MW359344	MW361054	MW359603	MW359855	MW360342	–	–	MW360808
*Trachypachus slevini* Van Dyke	T	MW359345	MW361055	MW359604	MW359856	DHK0513a	–	–	MW360809
** Carabidae **									
** Carabini **									
*Calosoma scrutator* (Fabricius)	1	EU677684	MW361056	MW359605	MW359857	EU677530	MW360567	EU677634	EU677661
** Cychrini **									
*Scaphinotus petersi* Roeschke	DR	EU658920	MW361058	MW359607	MW359859	EU677531	MW360569	EU677635	EU658922
** Metriini **									
*Metrius contractus* Eschscholtz	1	MW359346	MW361059	MW359608	MW359860	MW360343	MW360570	MW360097	MW360810
** Loricerini **									
*Loricera pilicornis* (Fabricius)	1	MW359347	MW361060	MW359609	MW359861	MW360344	MW360571	MW360098	MW360811
** Bembidiini **									
*Asaphidion yukonense* Wickham	DM	EU677686	–	–	–	EU677540	–	EU677638	EU677666
*Bembidion antiquum* Dejean	DM	EF648850	MW361061	EF649127.1	EF649127.1	EF649402	MW360572	MW360099	EF649489
** Pterostichini **									
*Pterostichus melanarius* (Illiger)	DM	AF398707	MW361062	MW359610	MW359862	EU677533	MW360573	MW360100	AF398623

^1^ Formerly assigned to subgenus Epinebriola. ^2^ Formerly assigned to subgenus Marggia. ^3^ Formerly assigned to subgenus Nebriorites. ^4^ Formerly assigned to subgenus Pseudonebriola. ^5^ Formerly assigned to subgenus Barbonebriola. ^6^ Formerly assigned to subgenus Patrobonebria. ^7^ Formerly assigned to subgenus Paranebria. ^8^ Formerly assigned to subgenus Asionebria. ^9^ Formerly assigned to subgenus Eunebria. ^10^ Formerly assigned to subgenus Reductonebria. ^11^ Formerly assigned to subgenus Nakanebria. ^12^ Formerly assigned to subgenus Catonebria.

All but nine of the specimens used in this study were collected directly into 95–100% ethanol, which was subsequently replaced with fresh ethanol at least once. Six specimens were collected directly into silica gel and allowed to fully desiccate. All specimens were stored as soon as possible in a freezer at -20 °C awaiting DNA extraction. The remaining three specimens were pinned museum specimens killed by unknown means. Depositories for all voucher specimens are listed in Appendix A.

### Identification

Nearctic *Nebria* and *Nippononebria* specimens were identified by DHK, based on his revision (Kavanaugh 1978) of that fauna with subsequent additions (Kavanaugh 1979a, 1981, 1984, 2008, 2015; Kavanaugh and Schoville 2009). Most Himalayan nebriite specimens were identified by JS and European and North African specimens by AF. Identifications of all Palearctic specimens were confirmed as far as possible by DHK using the worldwide revisions of *Nebria* and *Archastes* by Ledoux and Roux (2005, 2009), taxonomic keys and descriptions in papers listed in Table 2, or comparisons with holotypes or reliably determined museum specimens. Philippe Roux generously provided DHK access to his personal collection, which greatly facilitated identifications of specimens from China. Those specimens in our taxon sample that are without formal scientific names could not be identified by any of these means and are likely undescribed. The method used to identify each specimen is listed in Table 2.

### DNA extraction and sequencing

#### Specimens preserved for DNA

DNA was extracted from specimens preserved in ethanol or desiccated in silica gel using Qiagen DNeasy (Qiagen) Blood & Tissue kits and the manufacturer’s recommended protocol. The first 25 extractions (in 2004) were made using thoracic muscle tissue; but all subsequent extractions were made from one or more legs, depending on the size of the specimen. The eight gene fragments used in this study and their abbreviations are: **28S**: ca. 955 bases of the nuclear ribosomal DNA subunit 28S (D1–D3 regions); **16S-ND1**: ca. 812 bases of the mitochondrial gene fragment including 16S ribosomal RNA gene (partial sequence), tRNA-leucine gene (complete sequence), and NADH dehydrogenase subunit 1 (ND1) (partial sequence); **COIBC**: 658 bases of the “barcode” region of mitochondrial protein-coding cytochrome *c* oxidase subunit I; **COIPJ**: 829 bases of the ”Pat/Jer” region of mitochondrial protein-coding cytochrome *c* oxidase subunit I (no overlap with COIBC); **CAD2**: 804 bases of the nuclear protein-coding carbamoyl-phosphate synthetase domain of the “rudimentary” gene, corresponding to region 2 of Moulton and Wiegmann (2004); **PEPCK**: ca. 550 bases of the nuclear protein-coding phosphoenolpyruvate carboxykinase gene; **Topo**: ca. 550 bases of the nuclear protein-coding triosephosphate isomerase I gene; and ***wg***: 471 bases of the nuclear protein-coding *wingless* gene. For sake of simplicity of the text, these eight fragments will be referred to as “genes”, even though each corresponds to a portion of a gene or a portion of multiple genes.

Gene fragments were amplified successfully using polymerase chain reaction (PCR) on several different thermal cyclers, including an Eppendorf Mastercycler 5333, an MJ Research PTC-100 Thermal Cycler, or a BioRad MyCycler Thermal Cycler, and using different Taq polymerases, including Eppendorf Hotmaster Taq, Invitrogen Taq, or TaKaRa Ex Taq, each with the basic protocols recommended by the manufacturer. Cycling conditions and primers used in this study are presented in Appendix B. PCR amplification of PEPCK was unsuccessful for several taxa, in spite of as many as 10 attempts for some samples. PCR products were then cleaned, quantified, and sequenced at either the California Academy of Sciences’ Center for Comparative Genomics, using an Applied Biosystems 3130xl DNA Analyzer, or the University of Arizona’s Genomic and Technology Core Facility using either a 3730 or 3730 XL Applied Biosystems DNA Analyzer.

Multiple chromatograms for each gene fragment were assembled using SEQUENCHER (several versions, concluding with v 5.2.2) (Gene Codes Corporation) or PHRED (Green and Ewing 2002) and PHRAP (Green 1999) and the CHROMASEQ package in MESQUITE v3.2 (Maddison and Maddison 2016, 2017). Initial base calls were made using these programs. Early in the project, these base calls were checked using MACCLADE v4.08 (Maddison and Maddison 2005), and final modifications were made using CHROMASEQ and manual inspection. Multiple base reads at a single position were coded using the IUPAC ambiguity codes.

#### Pinned specimens

Three of the specimens from which DNA was extracted were pinned specimens whose killing and preservation history was unknown: *Nebria coreica* DRM4721, *Nebria komarovi* DRM5171, and *Nebria oxyptera* DRM5172. We prepared specimens following Kanda et al. (2015) and extracted DNA from whole or part of the body using a QIAamp DNA Micro kit (Qiagen) using the optional carrier RNA. Extraction was conducted in a laminar flow hood, sterilized with UV light before each use, in a clean room designed to minimize contamination from non-target DNA.

The extracted DNA was quantified using a Qubit Fluorometer (Life Technologies) with a Quant-iT dsDNA HS Assay Kit. The fragment length distribution was measured using a 2100 Bioanalyzer (Agilent Technologies) using the High Sensitivity DNA Analysis Kit and 1 μl of sample. The DNA of *Nebria oxyptera* was treated with NEBNext FFPE DNA Repair Mix (New England BioLabs) to repair damaged bases prior to library preparation. Libraries were prepared using NEBNext DNA Ultra II (New England BioLabs) kits and dual-indexed using NEBNext Multiplex Oligos for Illumina. Sequencing (150 base paired-end) was performed at the Oregon State University Center for Genome Research and Biocomputing on an Illumina HiSeq 3000. The *Nebria* libraries were sequenced on two separate lanes with libraries of other carabids (including bembidiines, chlaeniines, oodines, licinines, and scaritines). *Nebria coreica* DRM4721 was the only Nebriitae on its lane, while *Nebria komarovi* DRM5171 and *Nebria oxyptera* DRM5172 were sequenced on the same lane. In the latter case, the two *Nebria* libraries were given unique multiplex indices to minimize the possibility of adapter cross-talk.

Demultiplexing was performed using bcl2fastq v2 Conversion Software (Illumina). Paired-end reads were imported into CLC GENOMICS WORKBENCH version 9.5.3 (QIAGEN Aarhus A/S), using default options except for the minimum and maximum paired-read distances, which we determined by analyzing a dilution of the enriched library on a Bioanalyzer 2100 (Agilent Technologies). Failed reads were removed during import. We used the “Trim Sequences” tool in CLC (with default parameters) to remove read ends with low quality, ambiguous base calls, and adapter contamination.

De novo assemblies were generated using CLC GENOMICS WORKBENCH from paired, trimmed reads using an automatic word and bubble size, with the minimum contig length set to 200. The assemblies were converted to local, BLASTable databases using NCBI’s makeblastdb tool, and these databases were queried using the local BLAST tool in MESQUITE. The query sequences were those from Nebria (Boreonebria) gouleti DHK0027, chosen because it is expected to be equally distant from all expected placements for the pinned specimens that were sequenced. For each BLAST conducted, up to ten top matches from each database with eValues less than 1 E -80 were imported into the matrix in MESQUITE for analysis.

Two reference-based assemblies were conducted for each of the three pinned specimens. The first reference-based assembly for each used sequences of Nebria (Catonebria) piperi DHK0965 for the reference; the second used sequences from *Nippononebria virescens* DHK0348. Reference based assembly was performed using the “Map Reads to Reference” tool in CLC GENOMICS WORKBENCH. Default settings were used except for the “Length Fraction” and Similarity Fraction” parameters, which were both increased to 0.8 to decrease the possibility of spurious read mappings.

Some contigs produced by de novo and reference-based assemblies were discarded. For the de novo assembly, several samples yielded multiple contigs; in these cases, one of the contigs was much larger than the remainder (e.g., two contigs were returned when 16S-ND1 was BLASTed to the *Nebria komarovi* de novo assembly, one of 4677 bases, the other of 528 bases). In such cases, the shorter contig always appeared to be a contaminant, with one exception. The apparent contaminants were identical to a non-nebriine that was included in the multiplex pool on the same Illumina lane [e.g., *Clivina bipustulata* (Fabricius), *Chlaenius tomentosus* (Say)], suggesting that cross-talk occurred (Wright and Vetsigian 2016; Wang et al. 2017), even though the libraries were dual-indexed. The contaminant sequences were discarded. The one exception was a smaller fragment for *Nebria coreica*16S-ND1, which partly overlapped with the longer contig; in this case, the union of the contigs was created after alignment, and that union was the sequence analyzed. For the reference-based assemblies, some small contigs less than 250 bases were produced in addition to the longer ones; similar examination of the small ones also suggested that they represented contamination, or that they were for a conserved region of the gene and contained no signal for placement; these were also discarded. For each gene for each of the three pinned specimens, there are thus up to three sequences (one de novo sequence, and two reference-based sequences).

In order to judge the consistency of the two or three sequences (de novo assembly, reference-based to *Nippononebria*, and reference-based to *Catonebria*) from each pinned specimen, preliminary analyses were conducted on each of the seven genes and on the concatenated data. After model choice with JMODELTEST version 2.1.10 (Guindon and Gascuel 2003; Darriba et al. 2012) and PARTITIONFINDER 2.1.1 (Lanfear et al. 2012) (resulting in the same models as chosen for the finalized matrix, as described under Results), a search for ML trees was conducted with RAXML version 8.1.4 (Stamatakis 2014) using five search replicates. For all eight matrices, if there was more than one sequence for each gene for a specimen, then these formed a clade on the gene tree, with short branches, indicating that the data were at least consistent. The final gene sequence or a pinned specimen was formed from the union of the two or three sequences from different assembly techniques, with sites thereby having multiple states treated as ambiguities and assigned IUPAC codes.

#### Alignment and data exclusion

The gene fragments containing ribosomal DNA were aligned differently from the protein-coding genes. An alignment of 28S was conducted in MAFFT version 7.130b (Katoh and Standley 2013), using the L-INS-i search option and otherwise default parameter values. Boundaries of 16S, tRNA leu and ND1 were determined by aligning our sequences to the annotated complete mitochondrial genome of *Trachypachus inermis* (GenBank Accession NC_011329; Sheffield et al. 2008). However, the boundary between 16S and tRNA Leu was not completely clear; there were between zero and seven bases whose assignment was unclear in some taxa; these sites were excluded from further analyses. Once the boundaries were determined, the 16S region was aligned using MAFFT from within MESQUITE; the tRNA was separately aligned, again using MAFFT from within MESQUITE.

The methods used to align the protein-coding genes varied from gene to gene. COIBC, COIPJ, and Topo showed no evident insertion or deletion events in the history of the taxa studied, and so they could easily be aligned by inspection. Variation in *wg* suggested several insertion or deletion events present in the history of that taxa. This gene was aligned by first translating the sequence to amino acids in MESQUITE, aligning amino acids in MAFFT, and then forcing that amino acid alignment onto the original nucleotide alignment using MESQUITE. CAD2 and PEPCK contained introns, but few other insertions and deletions, so they were most easily aligned by inspection within MESQUITE’s data editors. CAD2 contained one intron present in *Trachypachus*, as well as one three-base insertion in *Metrius*; there were no insertions or deletions evident within nebriites. PEPCK contained one variable-length intron present in most nebriites as well as *Pterostichus* and *Scaphinotus*. In addition, there were two three-base deletions in two lineages of *Nebria*.

Site exclusion also varied from gene to gene. For the protein-coding genes, introns were excluded as well as starting region of PEPCK and Topo, as that region was sequenced in only a few taxa. For 28S and 16S-ND1, sites were excluded from consideration using the modified GBLOCKS (Talavera and Castresana 2007) analysis present in MESQUITE with the following options: minimum fraction of identical residues for a conserved position = 0.2, minimum fraction of identical residues for a highly-conserved position = 0.4, counting fraction within only those taxa that have non-gaps at that position, maximum length of contiguous non-conserved blocks = 4, minimum length of a block = 4, and allowed fraction of gaps within a position = 0.5. In addition, seven bases at the start and three bases at the end of 28S were excluded to make the starting and ending points more even across sequences.

#### Phylogenetic inference

Models of nucleotide evolution for single genes (except for the 16S-ND1 fragment) were chosen with the aid of JMODELTEST. For concatenated analyses and for the 16S-ND1 fragment, PARTITIONFINDER was used to determine the optimal partitioning, using the greedy algorithm and Bayesian Information Criterion (BIC). The beginning partition for the PARTITIONFINDER analysis had each codon position in each protein-coding gene in a separate part, and the components of the 16S-ND1 fragment in separate parts; thus, for the full concatenated matrix, the initial partition had 25 parts (three parts for each of COIBC, COIPJ, CAD2, ND1, PEPCK, Topo, and *wg*, as well as one part for each of 28S, 16S, tRNA-Leu, and the non-coding section of the 16S-ND1 fragment). For the final, concatenated matrix the “--raxml” option was used in PARTITIONFINDER, as otherwise program failure occurred during optimization.

Maximum likelihood estimates of phylogenetic relationships were reconstructed using RAXML version 8.2.12 (Stamatakis 2014) under the General Time Reversible (GTR) model of substitution rates, the gamma model of rate heterogeneity, an estimated proportion of invariable sites (GTRGAMMAI), and 100 independent heuristic searches. Non-parametric bootstrap support values were generated from 1,000 replicates employing the standard or “slow” bootstrap algorithm. Bootstrap (MLB) values were mapped onto the highest scoring likelihood tree generated from the 100 heuristic searches using MESQUITE’s Clade Frequencies in Trees feature. Separate RAXML analyses were performed on each of the eight genes. We also ran RAXML analyses on concatenated matrices of all eight genes (8GML), as well as of nuclear genes (Nuc G), nuclear protein-coding genes (NPC G), and mitochondrial gene fragments (Mito G).

Bayesian analyses were conducted on the eight-fragment concatenated matrix (8G B) with MRBAYES v 3.2.7 (Ronquist and Huelsenbeck 2003; Ronquist et al. 2012), using two runs with four chains each, with 100,000,000 generations run, sampling every 1,000 generations, and with a burn-in period of 25%. The average standard deviation of split frequencies was 0.00092 at the end of the run, with the effective sample size for all parameters being at least 550, as determined by Tracer v 1.7.1 (Rambaut et al. 2018). The final sample consisted of a total of 150,000 trees. Bayesian Posterior Probability (BPP) values are reported as percentages.

#### Support values

Bootstrap support for and against various clades was calculated using MESQUITE’s “Clade Frequencies in Trees” feature and the bootstrap trees produced by RAXML. Alternative trees with specific contradictory nodes were also included among the bootstrap support trees, and support values for or against those specific nodes were calculated by the same method. Bayesian posterior probability (BPP) values for and against clades were calculated in similar fashion, but using the trees produced by the Bayesian sampling.

#### Unique molecular synapotypies

To find unique molecular synapotypies for clades, MESQUITE’s “With State Distinguishing Selected Taxa” feature was used. In brief, the “Select Taxa in Clade” tool was used in the tree window, and then the “With State Distinguishing Selected Taxa” feature selected all characters with a unique state in the selected taxa. The unique state was then determined by visually examining those characters in the data matrix. Unique amino acids characterizing clades were found using the same procedure after converting each protein-coding gene to the implied amino acids using MESQUITE’s “Translate DNA to Protein” feature. To find unique base insertions and deletions in 28S sequences, the aligned matrix, including well-aligned internal portions of sequences excluded from ML and Bayesian analyses, was examined visually. Unique amino acid insertions and deletions in protein-coding gene sequences were identified by visually examining the matrix for each fragment converted to a protein matrix.

#### Data resources

New sequences generated in this study have been deposited in GenBank with accession numbers MW359101 through MW361062. Two files have been deposited in the Dryad Digital Repository, at https://doi.org/10.5061/dryad.6wwpzgmxn: (1) a NEXUS file containing aligned sequence data as well as inferred maximum likelihood trees, and (2) a NEXUS file containing data about PEPCK intron lengths and the ancestral state reconstruction shown in Suppl. material 2: Fig. S13.

## Results

### Sequencing

We were able to acquire sequence data for 97% (2041) of the 2112 possible sequences for the eight gene fragments for our 264 exemplar specimens (see Table 2). These included 1956 newly generated sequences and 85 sequences from GenBank (including 65 from Kavanaugh et al. 2011, three from Maddison 2008, two from Maddison et al. 2009, two from Ober 2002, two from Weng et al. 2020 and 11 from Wild and Maddison 2008). The numbers and percentages of sequences obtained are listed by gene fragment in Table 3.

**Table 3.** Numbers and percentages of sequences used in analyses by gene.

	28S	16S-ND1	COI PJ	COI BC	CAD	PEPCK	TOPO	*wg*	totals
specimens sampled	264	264	264	264	264	264	264	264	2112
sequences obtained	262	260	264	264	259	228	251	253	2041
% success	99	98	100	100	98	86	95	96	97

The 16S-ND1 sequences for *Nebria piute* DHK1098 and *N. catenata* DHK1067 each contain a stop codon in the ND1 gene, providing evidence that those particular sequences might be nuclear copies of the mitochondrial genes (“numts”, Thalmann et al. 2004) rather than the true mitochondrial copies, although we have no additional evidence of this (e.g., in the form of double peaks in the chromatograms). As these sequences appear in the phylogenies exactly where expected (see below), we have included these sequences in the analyses.

The only gene fragment for which we obtained less than 95% of the possible number was PEPCK; we speculate that failures in this gene may be the result of the variable-length intron within the gene, which may be long enough in some specimens to prevent amplification under the protocols we used. Of the 228 taxa from which we obtained sequences of PEPCK, 208 shared an intron, although the length of the intron varied between 27 and 830 bases. The intron was lacking from all outgroups except for *Pterostichus* and *Scaphinotus*; within nebriites it was absent from *Notiophilus*, Nebria (Parepinebriola), and the *Orientonebria*-*Archastes*-*Oreonebria* clade, but otherwise present. The only large group in which PEPCK was sequenced in all species was the *Catonebria* Series, a group in which the intron is relatively short.

### Models of evolution and partitions

GTR+I+G was chosen by the BIC for all single-gene analyses. This includes the 16S-ND1 fragment, for which PARTITIONFINDER found that the optimal partition had all sites in one part.

For the concatenated matrices, the best model scheme chosen by PARTITIONFINDER for all parts was GTR+I+G. The optimal partition scheme varied among concatenated matrices. For the eight-gene concatenated matrices (with or without the merging of the pinned specimens’ sequences), third positions of the mitochondrial genes were in one part, and all of the remaining sites in a second part. For the concatenated nuclear protein-coding gene matrix and concatenated nuclear gene matrix, all sites were in one part. For the concatenated mtDNA matrix, all third positions were in one part, with all remaining sites in a second part.

### Inferred phylogeny

From both ML and Bayesian analyses, we infer a well-resolved tree (Fig. 4 and Suppl. material 2: Fig. S1) with strong support in multigene analyses for most of the clades (Fig. 5, Chart 1). A fully resolved tree for the 256 nebriite taxon samples in our matrix would have had 255 internal nodes, including the basal node for nebriites. Our ML bootstrap tree for the concatenated eight-gene matrix (8GML) (Fig. 5) had 229 internal nodes, of which 167 (65% of the possible 255 internal nodes) had MLB support of 90% or more, 194 (76%) had support of 75% or more, and 211 (83%) had support of 60% or more. The majority rule consensus tree from our Bayesian analysis (8G B) of the same matrix (Suppl. material 2: Fig. S1) had 253 internal nodes and thus was 99% resolved. Of these nodes, 228 (89%) had BPP values of 90 or more, 236 (93%) had values of 75 or more, and 244 (96%) had values of 60 or more. The 8GML and 8G B trees were very similar in structure, with all but 18 of the 229 clades (92%) in the 8GML tree shared with the 8G B tree. Those not shared are identified in the ML tree (Fig. 4) by an open circle on basally pale branches. Thirteen of the 18 differing clades were found within the *Catonebria* Series, the most densely sampled part of the tree. Instances of ambiguity are addressed where appropriate in the Discussion.

**Chart 1.**
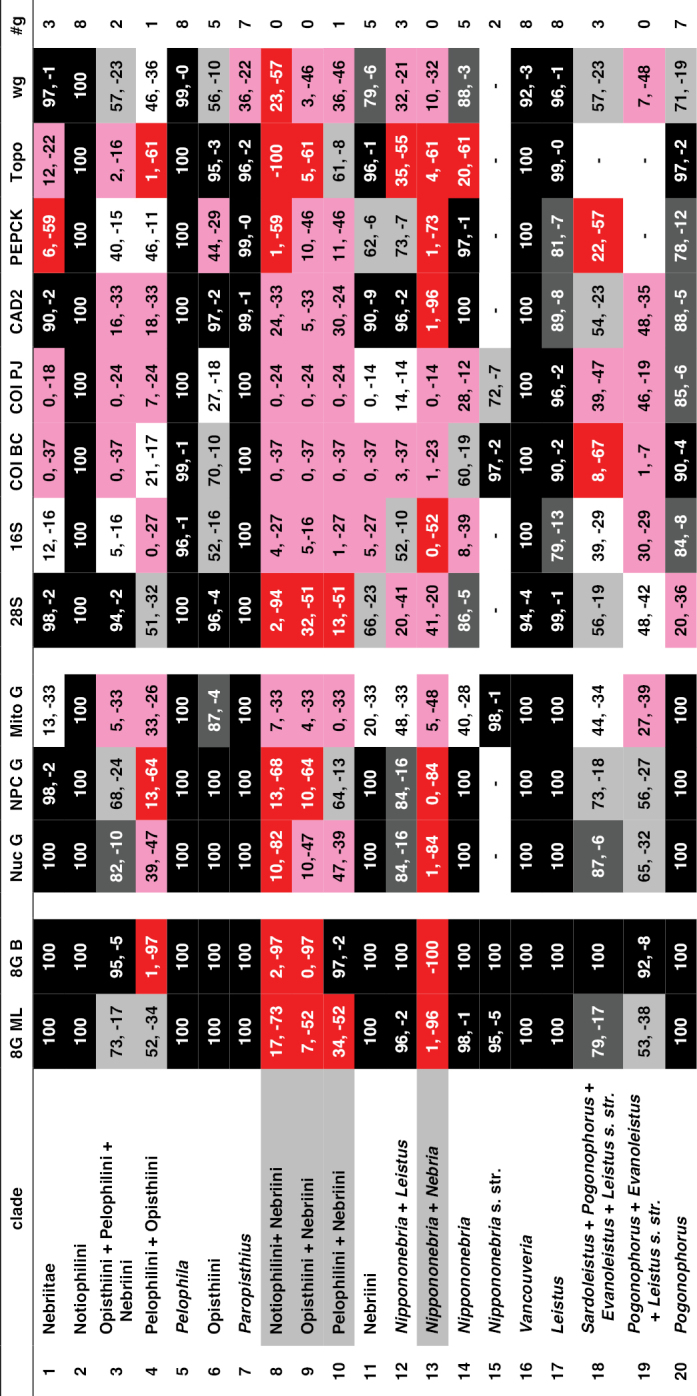
Support for or against various clades. All columns provide maximum likelihood bootstrap values for or against a particular clade, except for column “8G B,” which shows the Bayesian posterior probability estimates for the eight-gene matrix. “8GML” shows the bootstrap values for the eight-gene concatenated matrix, “Nuc G” for the concatenated nuclear genes, “NPC G” for the concatenated nuclear protein-coding genes, and “Mito G” for the concatenated mitochondrial genes. The remaining eight columns provide values for the single gene analyses. All values are expressed as percentages, with positive numbers indicating support for a clade and negative numbers indicating support for a contradictory clade having the highest support. Specific contradictory clades from alternative trees are highlighted in medium grey. Cells with bootstrap values ≥ 90 are shown in black, with values between 75 and 89 in dark grey, and values from 50 to 74 in light grey. Cells in white indicate clades present in the ML tree, but with bootstrap values < 50. Cells in red have bootstrap values for a contradictory clade ≥ 50. Cells in pink have bootstrap values for or against a clade < 50, and the clade is not present in the ML tree. A “-“ in a cell indicates that taxon sampling for that gene was not sufficient to assess monophyly of that clade. “#g” shows the number of single-gene analyses (maximum of eight) that support a clade with bootstrap values of 50 or more.

**Chart 1.**
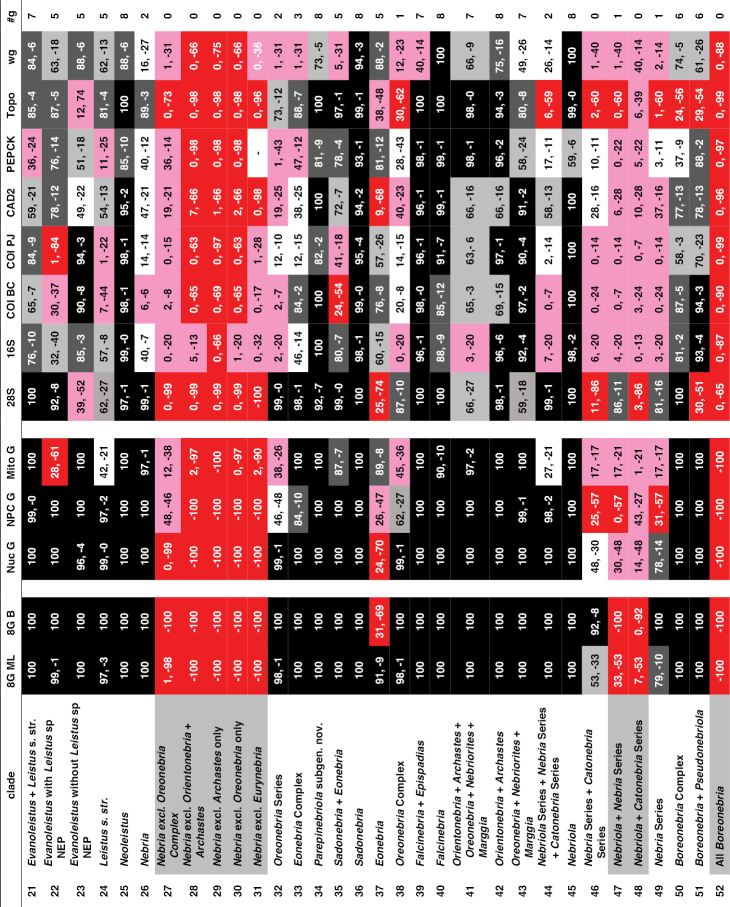
Continued.

**Chart 1.**
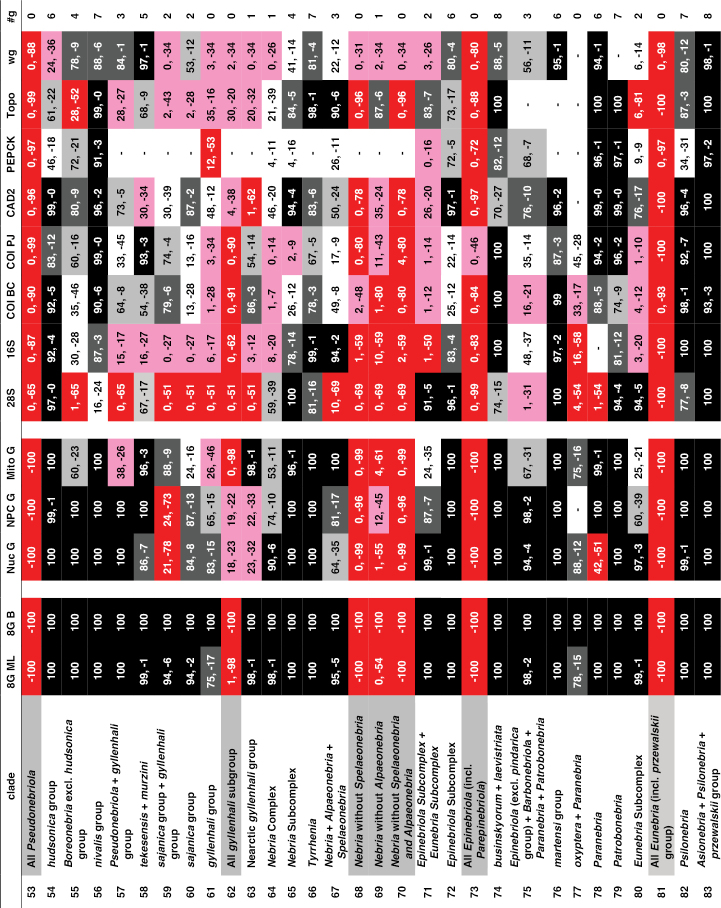
Continued.

**Chart 1.**
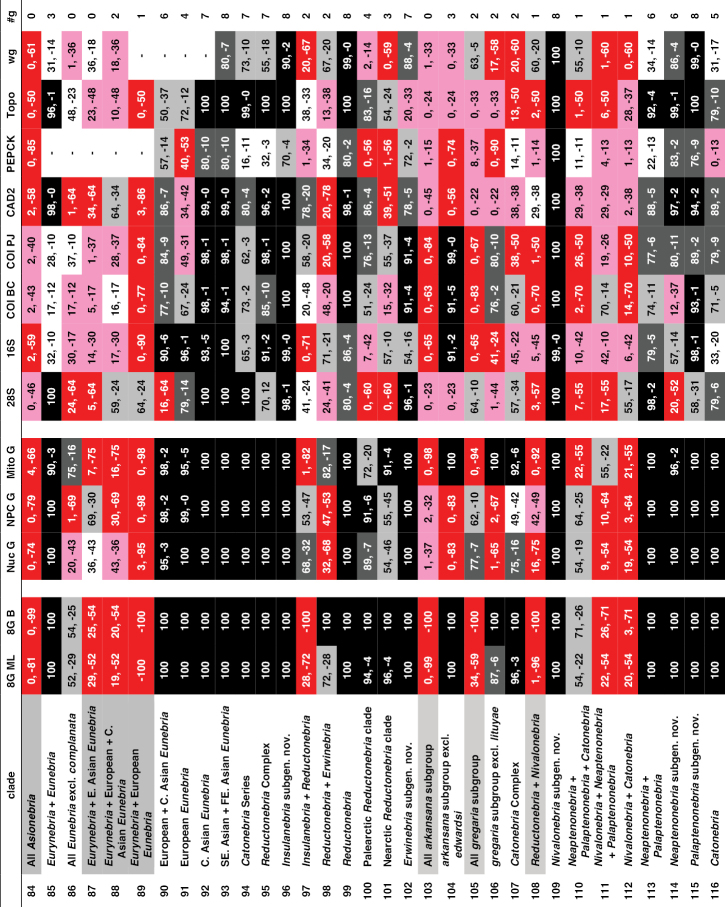
Continued.

**Figure 4.**
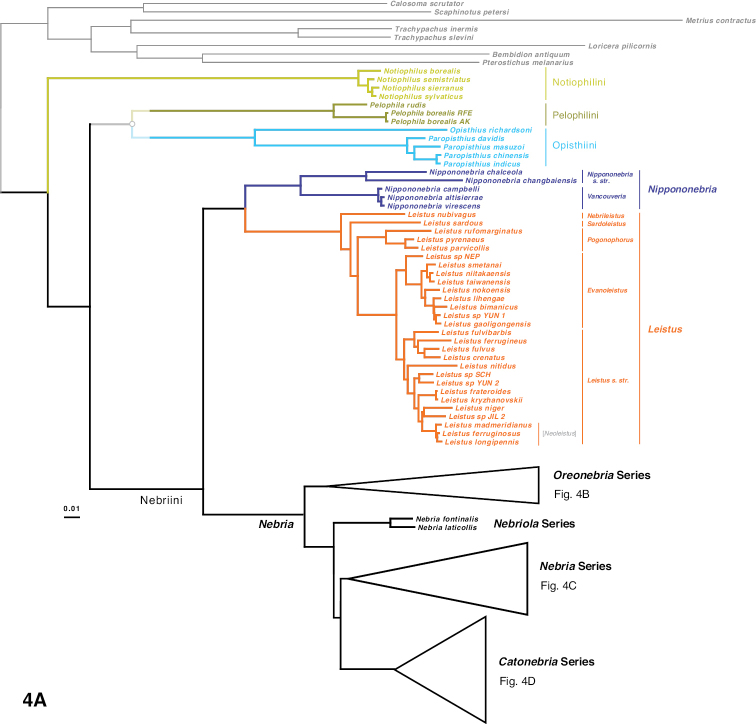
Maximum likelihood tree for concatenated matrix of all genes. Scale bar: 0.1 units, as estimated by RAXML.

**Figure 4.**
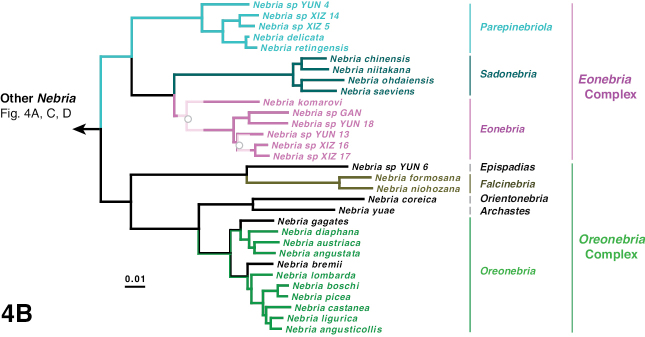
Continued.

**Figure 4.**
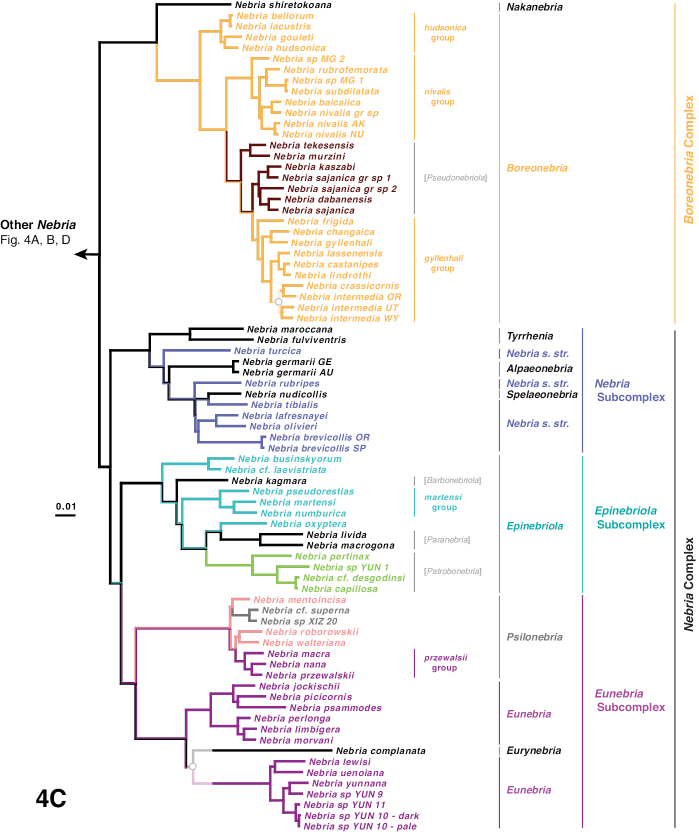
Continued.

**Figure 4.**
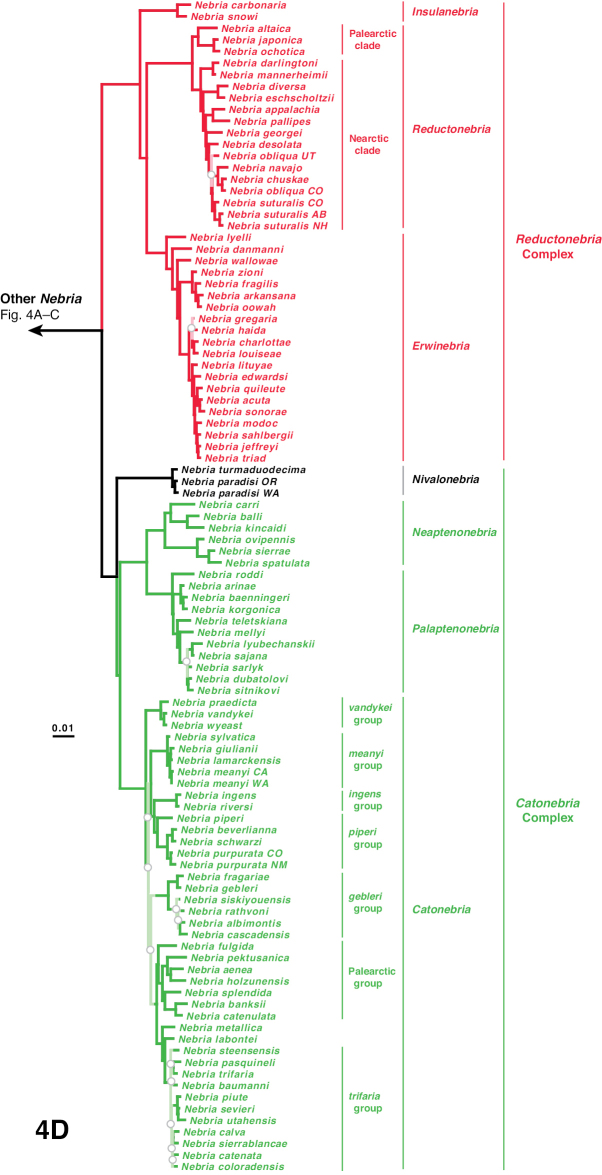
Continued.

**Figure 5.**
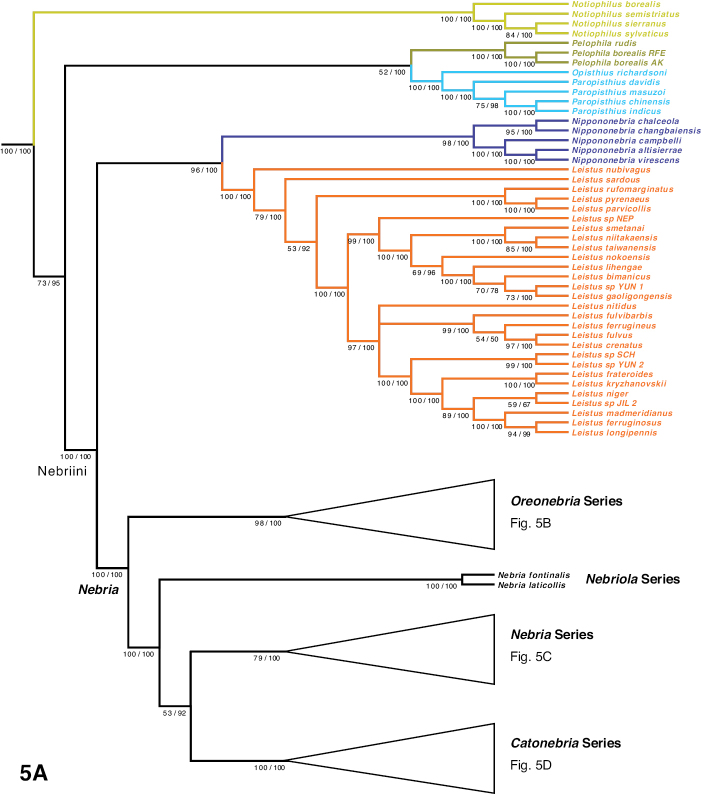
Majority rule consensus tree of trees from bootstrap replicates. The first number under a branch is the percentage of bootstrap replicates with that clade, the second number is the estimate of the Bayesian posterior probability of that clade expressed as a percentage.

**Figure 5.**
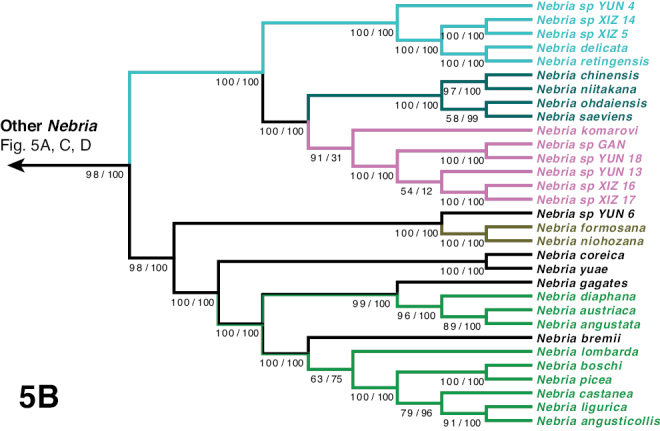
Continued.

**Figure 5.**
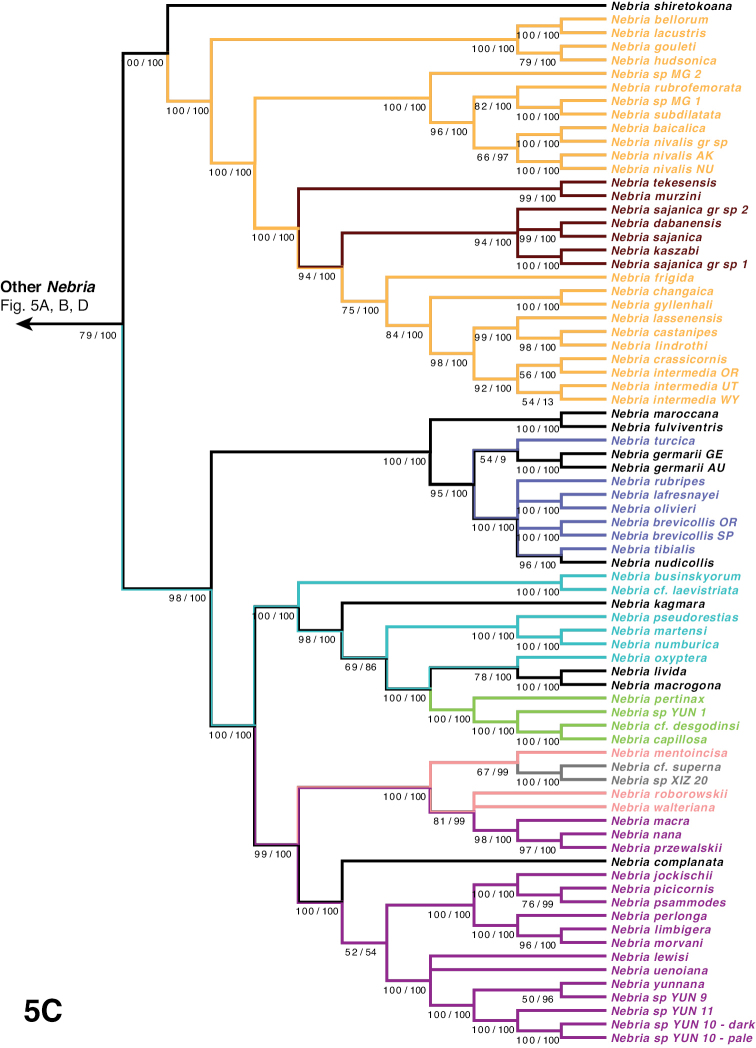
Continued.

**Figure 5.**
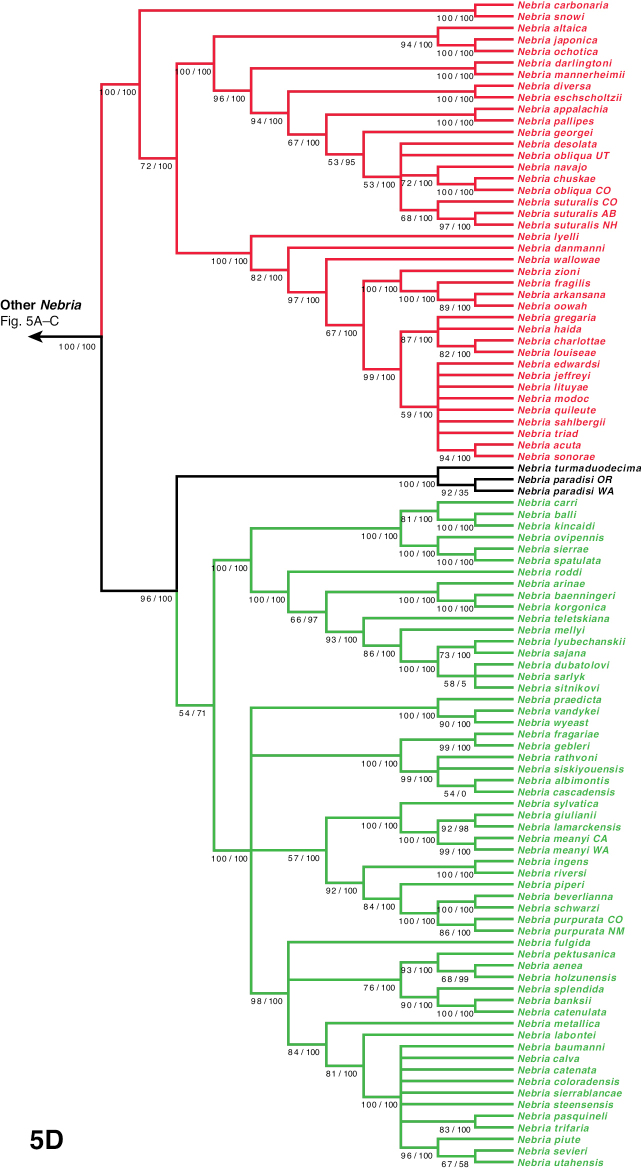
Continued.

ML trees and majority rule consensus trees of bootstrap trees resulting from analyses of concatenated matrices for Nuc G, NPC G, and Mito G and for each of the single-gene matrices are presented in Suppl. material 2: Figs S2–S12. Support for and against particular clades from each of the concatenated and single-gene analyses is summarized in Chart 1. Phylogenetic results of interest are discussed in detail in the Discussion.

Unique bases found to support particular clades are listed in Suppl. material 1: Table S1, unique amino acids in Suppl. material 1: Table S2, and unique insertions and deletions in Suppl. material 1: Table S3.

The intron length of PEPCK varies across the phylogeny, with some clades having small or no introns, and with a few clades with longer introns (Suppl. material 2: Fig. S13).

## Discussion

### Monophyly of Nebriitae

Morphology has provided only weak support for the monophyly of a supertribe Nebriitae because most features that have been used to characterize it are both plesiomorphic within carabids and not unique to this group. Kavanaugh and Nègre (1983) suggested that the only known synapomorphy for the entire group is the asetose parameres of the male genitalia. Arndt (1993) suggested three additional larval morphological synapomorphies. In fact, the most parsimonious tree for a matrix of 153 morphological characters suggested that the Nebriitae represent a grade rather than a clade (Kavanaugh 1998). Other “basal grade” carabid groups, including cicindines (Erwin 1991; Lorenz 2005), gehringiines, omophronines, elaphrines (Liebherr and Will 1998) and a few other lineages, have been suggested as members of the Nebriitae, so both the monophyly and the inclusiveness of the supertribe have remained uncertain.

Based on results from analyses of our molecular data, we find very strong evidence for a monophyletic Nebriitae (Fig. 4; Chart 1, line 1), of course with *Notiokasis* unsampled for the present. Both our ML (Fig. 5) and Bayesian analyses (Suppl. material 2: Fig. S1) of the eight-gene matrix show support (MLB and BPP) values of 100 for this clade. ML analyses of concatenated nuclear gene (Suppl. material 2: Fig. S2) and nuclear protein-coding gene (Suppl. material 2: Fig. S3) matrices, as well as of single-gene matrices for 28S, CAD2 and *wg* (Suppl. material 2: Figs S5, S9 and S12, respectively) show MLB values ≥ 90. This clade also appears in the tree for concatenated mitochondrial genes (Suppl. material 2: Fig. S4), albeit with weak support. Monophyly of the supertribe is also supported by three unique amino acids (Suppl. material 1: Table S2). Evidence contradictory to this clade is provided by ML analyses of single-gene matrices for PEPCK (Suppl. material 2: Fig. S10), Topo (Suppl. material 2: Fig. S11), COIBC (Suppl. material 2: Fig. S7) and COIPJ (Suppl. material 2: Fig. S8), each of which shows one or more of notiophilines, opisthiines, or pelophilines more closely related to one or more of the outgroup taxa than to the other nebriite taxa but with only weak support and no consistent pattern of relationship. Because we were unable to include *Notiokasis* in our sample, our conclusions about nebriite monophyly cannot yet include the Notiokasiini. A more thorough test of the monophyly of nebriites would not only include *Notiokasis*, but also a more thorough sampling of outgroups.

### Monophyly of the nebriite tribes and relationships among them

Monophyly of the tribes Notiophilini, Pelophilini, and Opisthiini are well supported morphologically (Ball and Bousquet 2001), and our results strongly support their being clades. Analyses of all concatenated and single-gene matrices produced MLB and BPP values of 100 for both the Notiophilini and Pelophilini (Chart 1, lines 2 and 5, respectively). Monophyly of each of these two tribes is supported also by unique amino acids (19 and two, respectively; Suppl. material 1: Table S2). The notiophiline clade is also supported by one unique base insertion and one unique deletion in 28S and by one amino acid insertion and three amino acid deletions in *wg* (Suppl. material 1: Tables S3). The pelophiline clade is also supported by 18 unique bases (Suppl. material 1: Tables S1). Our ML and Bayesian analyses of the eight-gene matrix and ML analyses of the concatenated nuclear gene and nuclear protein-coding gene matrices produced support values of 100 for Opisthiini (Chart 1, line 6). Opisthiine monophyly was also strongly supported in single-gene analyses for 28S, CAD2 and Topo, and moderately supported in the concatenated mitochondrial gene analysis (Chart 1, line 6), as well as by one unique amino acid and seven unique bases (Suppl. material 1: Tables S1, S2). *Paropisthius* is also strongly supported as monophyletic in all analyses (MLB and BPP values > 95) except for *wg* (Chart 1, line 7), where it does not appear in that resulting tree (Suppl. material 2: Fig. S12). It is also supported by five unique amino acids and 17 unique bases (Suppl. material 1: Tables S1, S2) and by a unique three-base insertion in 28S (Suppl. material 1: Table S3).

In spite of the fact that the diverse Nebriini is not well characterized morphologically, being recognized mainly on the basis of symplesiomorphic features, monophyly of the tribe is strongly supported by our molecular data. Both ML and Bayesian analyses of the eight-gene matrix and ML analyses of the concatenated nuclear gene and nuclear protein-coding gene matrices produced support values of 100 for the tribe (Chart 1, line 11). Nebriini is also strongly supported in analyses for CAD2 and Topo, moderately supported in the *wg* analysis and weakly supported in the 28S and PEPCK analyses (Chart 1, line 11), as well as by one unique amino acid (Suppl. material 1: Table S2). The nebriine clade appears in six of the eight single-gene trees (Suppl. material 2: Figs S5–S12).

Phylogenetic relationships among the nebriite tribes remain somewhat less clearly defined than their monophyly. Our results show moderate to strong support for a clade including Opisthiini, Pelophilini and Nebriini as sister to Notiophilini (Fig. 5; Chart 1, line 3). Strongest support (≥ 94) for this clade comes from the eight-gene Bayesian analysis (BPP = 95) and the single-gene 28S analysis (MLB = 95) with moderate additional support from the concatenated nuclear gene analysis (MLB = 82) and from one unique amino acid (Suppl. material 1: Tables S1) and one unique amino acid insertion in the *wg* gene fragment (Suppl. material 1: Table S3) not seen in notiophilines or any of the outgroups. We found no support for clades including Nebriini + Notiophilini (Chart 1, line 8) or Nebriini + Opisthiini (Chart 1, line 9). Our results about tribal relationships within nebriites should be considered with some caution, as they are an old enough group with distant enough relatives that determining the placement of the root within Nebriitae may not be easy. For example, it is possible that notiophilines are reconstructed as the sister to other nebriites as they are on a long branch, which might then be the place of attachment to the relatively minimally sampled and thus long-branched outgroups through long-branch attraction (Felsenstein 1978).

**Figure 6.**
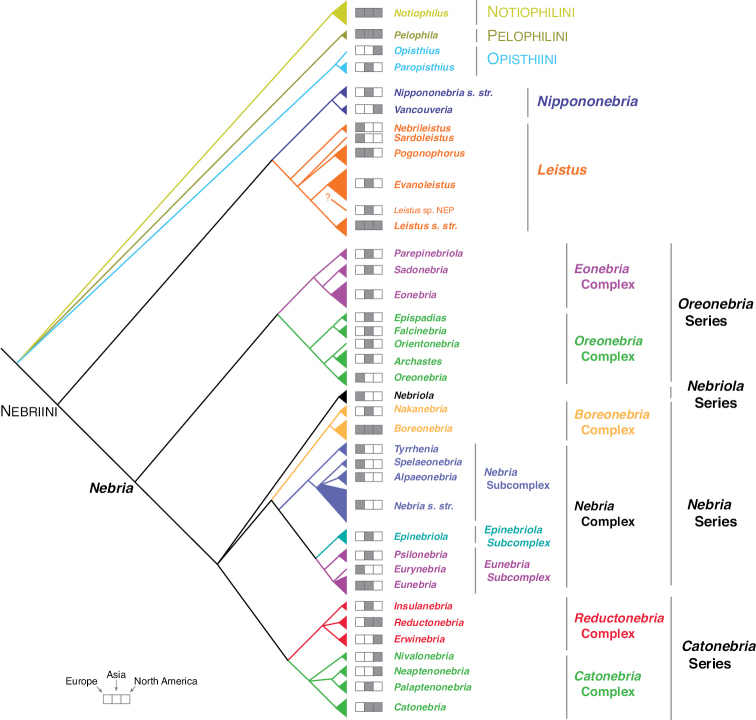
Summary tree of nebriite phylogeny illustrating the revised classification; clade representation in Europe (including North Africa and the Middle East), Asia, and North America is indicated in the three-box bar.

Our results do not strongly speak to the treatment of *Pelophila* as belonging to a separate monogeneric tribe. *Pelophila* had been included among the Nebriini (see Thomson 1859; Ganglbauer 1891a; Reitter 1908; Ball 1960; Lindroth 1961) until Kavanaugh (1996) proposed separate tribal status for it based a parsimony analysis of morphological data that found a clade including Notiophilini + Notiokasiini + Opisthiini more closely related to other nebriines than was *Pelophila*. In a second parsimony analysis using a slightly different morphological dataset, Kavanaugh (1998) found *Pelophila* more closely related to other nebriines than was a clade including Notiokasiini + Notiophilini + Opisthiini; but in this reconstruction, the Nebriitae represented a grade rather than a clade. Ambiguity in the relationships of *Pelophila* to the other nebriites is reflected in our molecular results as well. A clade including *Pelophila* and the opisthiines (Chart 1, line 4) is supported weakly (MLB = 51-52) in the ML eight-gene concatenated and 28S bootstrap analyses and it appears in only half of the single-gene ML trees (28S, COIBC, PEPCK, and *wg*) (Suppl. material 2: Figs S5, S7, S10, S12). It is supported also by one amino acid unique for the entire taxon sample and three others unique among nebriites and by a single unique base (Suppl. material 1: Tables S1, S2). Conversely, a clade including *Pelophila* with the other nebriine genera (Suppl. material 2: Fig. S1) has strong support from the Bayesian eight-gene concatenated analysis (BPP = 97) and weak support from the ML concatenated nuclear protein-coding gene bootstrap analysis (MLB = 64) and the Topo bootstrap analysis (MLB = 61) (Chart 1, line 10); but this clade appears only in the single-gene ML tree for Topo (Suppl. material 2: Fig. S12). This difference in results from the ML and Bayesian eight-gene concatenated analyses is the single most important disagreement between them, so at least for the present, we continue to treat Pelophilini as a distinct nebriite tribe but part of an unresolved polytomy with notiophilines, opisthiines, and nebriines (see overview tree of nebriite phylogeny, Fig. 6).

### Relationships among the Nebriini

As noted in the Introduction, there have been differences of opinion among taxonomists concerning the number of distinct genera that should be recognized among the Nebriini as defined here (i.e., excluding *Pelophila*). Nevertheless, *Leistus* has been universally accepted as a distinct genus, based on its wonderful suite of mouthpart co-adaptations for small prey capture. *Nebria* also has been universally regarded as a distinct genus, but its inclusiveness has differed among authors. In the following sections, we discuss how our results provide new evidence to support phylogenetic relationships among *Leistus* subgenera and species and better determine which groups are part of a monophyletic genus *Nebria* and which ones are not.

### Phylogeny of *Leistus*

Perrault (1980) introduced and later modified (Perrault 1991) a classification of *Leistus* that is currently in wide use, recognizing six subgenera. His conclusions were based mainly on detailed studies of adult mouthparts and male genitalia. Particularly striking are the differing degrees of development and fusion of digitiform processes on the maxillary stipes and submentum on which stout, spiniform setae insert, as well as differences in the form and development of the trident-like anterior extension of the ligular sclerite seen in members of this genus. Our results based on analyses of molecular data provide support for his classification and the phylogenetic relationships he proposed within the genus.

The monophyly of genus *Leistus* is strongly supported in all of our concatenated and single-gene analyses (see Fig. 5A; Chart 1, line 17) as well as by one unique amino acid, one unique base and three unique base deletions in 28S (Suppl. material 1: Tables S1–S3).

We had only one of the two known species of subgenus Nebrileistus Bänninger, 1925 in our sample, so we cannot comment further on its monophyly. In our analyses, *Nebrileistus* is shown as sister to a clade including the other *Leistus* subgenera (Figs 4A, 5A; Chart 1, line 18) that is very strongly supported as monophyletic in the 8G B analysis (BPP = 100) (Suppl. material 2: Fig. S1), slightly less strongly supported by the 8GML (Fig. 5A), Nuc G (Suppl. material 2: Fig. S2) and NPC G (Suppl. material 2: Fig. S3) and moderately supported by analyses for 28S (Suppl. material 2: Fig. S5), CAD2 (Suppl. material 2: Fig. S9) and *wg* (Suppl. material 2: Fig. S12). That clade is also supported by one unique amino acid (Suppl. material 1: Table S2). This relationship is not supported by any of the mitochondrial genes in single-gene or concatenated bootstrap analyses, but still is shown in the ML trees from both the Mito G and 16S-ND1 analyses (Suppl. material 2: Figs S4, S6–S8). A sister group relationship of *Nebrileistus* to a clade including all other *Leistus* is consistent with the absence of several relatively apomorphic morphological features present in their members.

Subgenus Sardoleistus Perrault, 1980 is monobasic, as it includes only *Leistus sardous* Baudi di Selve, 1883. Results from our analyses suggest that this subgenus is sister to a clade including *Pogonophorus* Latreille, 1802, *Evanoleistus* Jedlička, 1965 and *Leistus* s. str. (Fig. 4A), but support for the latter clade is mixed (Chart 1, line 19). It is strongly supported in the 8G B analysis (BPP = 92) (Suppl. material 2: Fig. S1) but only weakly supported in the 8GML (Fig. 5A), Nuc G (Suppl. material 2: Fig. S2) and NPC G (Suppl. material 2: Fig. S3) analyses. The clade does not appear in ML trees from the Mito G analysis (Suppl. material 2: Fig. S4) or from any of the single-gene analysis except for 28S (Suppl. material 2: Fig. S5), where it has low MLB support. Other possible relationships suggested in individual analyses include (1) a sister-group relationship to all other *Leistus* subgenera (Suppl. material 2: Figs S7–S9), or (2) sister to a clade including only *Evanoleistus* and *Leistus* s. str. (Suppl. material 2: Fig. S4), or (3) as part of an unresolved trichotomy with *Pogonophorus* and *Evanoleistus* + *Leistus* s. str. (Suppl. material 2: Figs S6, S12).

The subgenus Pogonophorus is strongly supported as monophyletic in all concatenated gene analyses and all single-gene analyses except for 28S (Chart 1, line 20). In the tree from the 28S analysis (Suppl. material 2: Fig. S5), *Pogonophorus* is paraphyletic in an unresolved trichotomy with a clade including subgenera *Evanoleistus* + *Leistus* s. str. Because our sample included only ca. 5% of the known diversity currently included in this subgenus (there are 40 species and more than 20 subspecies beyond the three species we sampled) as well as only a limited geographical sample (European species only) of the range of this taxon, monophyly for the subgenus should be confirmed with additional sampling.

A clade including *Evanoleistus* and *Leistus* s. str. is strongly supported as monophyletic in all concatenated gene analyses and strongly to moderately supported in all single-gene ML analyses except for PEPCK (Chart 1, line 21). It is also supported by one base unique among nebriites (Suppl. material 1: Table S1) and by two unique base deletions in 28S (Suppl. material 1: Table S3). In the ML tree from the PEPCK single-gene analysis (Suppl. material 2: Fig. S10), groups of species from both subgenera are shown in an unresolved polytomy that also includes *Leistus nubivagus* Wollaston, 1864 (i.e., subgenus Nebrileistus), a pattern of relationships not seen in any of the other single-gene or concatenated gene trees. We therefore suggest that *Evanoleistus* and *Leistus* s. str. together form a clade. As noted below, each of them is strongly supported as monophyletic in most of our concatenated gene analyses. However, the phylogenetic distinction between them is not yet completely clear.

Evanoleistus is the most diverse subgenus of Leistus with more than 150 species described. Our sample included only nine species, two of which are undescribed, so our sampling was small (ca. 5%) relative to that diversity and our results must be judged in that light. Nonetheless, the species in our sample form a monophyletic group (Figs 4A and 5A) that is strongly supported in all the concatenated gene analyses except the Mito G analysis (Chart 1, line 22) and also in the 28S and Topo analyses (Suppl. material 2: Figs S5 and S11, respectively). This clade also appears in ML trees from CAD2, PEPCK, and *wg* analyses (Suppl. material 2: Figs S9, S10, and S12, respectively) but with only moderate support. Evidence against a monophyletic *Evanoleistus* clade for the species in our sample involves only the mitochondrial genes and mainly the relationships of *Leistus* sp NEP to the other species of *Evanoleistus* and to *Leistus* s. str. In the ML tree from the Mito G analysis (Suppl. material 2: Fig. S4), *L.* sp NEP is part of a clade with the species of *Leistus* s. str. rather than with *Evanoleistus* species, but that clade has only moderate support (MLB = 61). Stronger support (MLB = 84) for this same clade is provided by the COIPJ analysis (Suppl. material 2: Fig. S8). In the tree from the 16S-ND1 analysis (Suppl. material 2: Fig. S6), *L.* sp NEP is shown as sister to a clade including the remaining *Evanoleistus* species plus all the *Leistus* s. str. species, but with only weak support (MLB = 40); and in the tree from the COIBC analysis (Suppl. material 2: Fig. S7), it is part of a weakly supported clade with the other *Evanoleistus* species that also includes *Leistus nitidus* (Duftschmidt,1812) from among our *Leistus* s. str. species. A clade that includes all the *Evanoleistus* species excluding *L.* sp NEP (Chart 1, line 23) is strongly supported in all concatenated gene analyses, including the Mito G analysis, in all three mitochondrial and in the *wg* single-gene analysis, but it is only moderately supported in CAD2 and PEPCK analysis and contradicted in the 28S and Topo analyses. The above results suggest that *Leistus* sp NEP is unique among the species in our sample, representing either a sister taxon to all our other *Evanoleistus* or an outlier related more closely to one or more species of *Leistus* s. str. As the only Himalayan species in our sample out of the 18 species described in *Evanoleistus* from that region, it may represent a more diverse lineage with relationships intermediate between *Evanoleistus* and *Leistus* s. str. We therefore include *L.* sp NEP in an unresolved trichotomy in our summary tree (Fig. 6) to reflect this ambiguity. Molecular data from additional members of the Himalayan fauna may help clarify relationships.

*Leistus* s. str. currently includes ca. 50 species and our sample represents a little more than 20% of them. Our sample of this subgenus is strongly supported as monophyletic in all concatenated analyses except for the Mito G analysis (Chart 1, line 24), in which it is only weakly supported (MLB = 42). This clade is shown also in ML trees for all but three of the single-gene analyses (COIBC, COIPJ and PEPCK; Suppl. material 2: Figs S6–S8, respectively) but with lower support values. Contradictory clades involve the inclusion of *L.* sp NEP from *Evanoleistus* in *Leistus* s. str. (MLB = 84; COIPJ tree, Suppl. material 2: Fig. S8), the inclusion of *L. nitidus* instead in *Evanoleistus* (COIBC tree, Suppl. material 2: Fig. S7), and, in the PEPCK tree (Suppl. material 2: Fig. S10), *Leistus crenatus* Fairmaire, 1855 and *L. ferrugineus*, the type species of *Leistus*, in a clade with *L. nitidus* that is more closely related to the *Evanoleistus* species than to the remaining “*Leistus* s. str.” species.

Erwin (1970) described subgenus Neoleistus for the three endemic *Leistus* species in western North America. Perrault (1980) originally treated *Neoleistus* as a junior synonym of *Leistus* s. str.; but later (Perrault 1991) assigned species of his *niger* group of *Leistus* s. str. to *Neoleistus* and resurrected it as a valid subgenus. Lorenz (2005) followed Perrault’s reclassification, but Farkač (2016) retained the Palearctic species (*niger* group) in *Leistus* s. str. Our results confirm a monophyletic North American clade (Figs 4A, 5A) that is strongly supported in all concatenated and single-gene analyses (Chart 1, line 25). However, this clade is deeply nested within subgenus Leistus s. str., so recognizing it as a separate subgenus would render Leistus s. str. paraphyletic. We therefore consider *Neoleistus* as a junior synonym of *Leistus* s. str.

### Status of *Nippononebria*

Uéno (1955) described *Nippononebria* as a subgenus of *Nebria*. Ledoux and Roux (2005) treated *Nippononebria* and *Vancouveria* as separate subgenera in their “Nippononebrides” group of subgenera within the “Vetanebri”, one of their two primary divisions of *Nebria*. In contrast, Kavanaugh (1995, 1996), had proposed that *Nippononebria* should be considered as a genus distinct from *Nebria* and with *Vancouveria* (Fig. 2D) as its subgenus and, further, that this clade was sister to *Leistus* rather than to *Nebria*. This conclusion was based on phylogenetic analyses of morphological data, as was that of Ledoux and Roux. However, the latter do not appear to have included morphological data for *Leistus*, *Archastes*, or any genera from other nebriite tribes in their formal analysis of *Nebria*, and this limitation would have prevented them from recognizing a sister group relationship between *Nippononebria* and any other group except *Nebria*.

Results from our analyses of molecular data provide strong support for a clade including only *Nippononebria* and *Leistus* (Chart 1, line 12). Our 8GML, 8G B, Nuc G, and NPC G concatenated analyses and CAD2 single-gene analyses show strong support for this clade, PEPCK shows moderate support, and Mito G and 16S-ND1 show weak support. Conversely, a clade including only *Nippononebria* and *Nebria* (Chart 1, line 13) is unsupported, except weakly so by the 28S single gene analysis (MLB = 41). Monophyly of *Nippononebria* (including both *Nippononebria* s. str. and *Vancouveria*) (Chart 1, line 14) is also strongly supported in all concatenated analyses except Mito G and in 28S, CAD2, PEPCK and *wg* analyses. Two unique amino acids also support this clade (Suppl. material 1: Table S2). Subgenera *Nippononebria* s. str. (Chart 1, line 15) and *Vancouveria* (Chart 1, line 16) are both strongly supported as monophyletic by all available results, as well as by three and five unique amino acids, respectively (Suppl. material 1: Table S2). The fossil *Archaeonebria inexspectata*, recently described from Baltic amber and dated at 50–35 Mya (Schmidt et al. 2019), appears to be a stem *Nippononebria* and, as such, supports our molecular results in attesting to a deep split between this lineage and *Leistus*. We conclude that *Nippononebria* should be classified as a nebriine genus distinct from both *Nebria* and *Leistus* and more closely related to the latter (Figs 4A, 6). An alternative change would be to expand the scope of *Nebria* to include *Nippononebria*, *Archaeonebria* and *Leistus*, which we view as excessive consolidation obscuring rather than clarifying relationships.

### Status of *Archastes*

Unlike *Nippononebria*, *Archastes* (Fig. 2C) was described as a distinct nebriine genus (Jedlička 1935) and has always been treated as such (Ledoux and Roux 2009). All of the 37 described species share a similar general body form and habitus, all are flightless and together they occupy a relatively small area, mainly in central Sichuan Province, China. We had only one species represented in our sample, but for the purpose of exploring the phylogenetic relationships of *Archastes* to other nebriines and to *Nebria* in particular, this sample may be sufficiently representative, given the morphological and geographical cohesion of the group.

All our concatenated analyses, as well as 28S and Topo single-gene analyses, very strongly support a clade including *Archastes* within *Nebria* (Chart 1, line 26). Conversely, a *Nebria* clade excluding *Archastes* is strongly contra-indicated. This is shown whether *Archastes* is excluded alone (Chart 1, line 29) or along with the other *Nebria* subgenera to which our results show close its relationship (see further discussion below and Chart 1, lines 27 and 28). Consequently, we conclude that *Archastes* does not warrant status as a separate genus, but should be classified within genus *Nebria*.

### Status of *Oreonebria*

Daniel (1903) described *Oreonebria* (Fig. 2E) as a subgenus of *Nebria*. Jeannel (1937, 1941) recognized this taxon as a genus separate from *Nebria*, based mainly on differences between the two groups in male genitalic features. Huber (2004, 2017) and Lorenz (2005) followed Jeannel in giving *Oreonebria* generic status, but Ledoux and Roux (2005) included it as a subgenus in their treatment of *Nebria*.

Just as for *Archastes*, our analyses strongly support a monophyletic *Nebria* including *Oreonebria* (Chart 1, line 26), and a *Nebria* clade excluding *Oreonebria* is strongly contra-indicated. This is shown whether *Oreonebria* is excluded alone (Chart 1, line 30) or along with the other *Nebria* subgenera found from our analyses to be closely related (see further discussion below and Chart 1, lines 27). We conclude that *Oreonebria* does not warrant status as a separate genus and should be classified within genus *Nebria*.

### Status of *Eurynebria*

*Eurynebria* was described as a new genus by Ganglbauer (1891b) with *Nebria complanata* (*Carabus complanatus* Linnaeus, 1758) (Fig. 2F) as the type species. Jeannel (1937, 1941), followed by Lorenz (2005) and Huber (2017), treated this taxon as a distinct genus. Although *N. complanata* exhibits several autapomorphic features, such as more setae on the labrum and penultimate labial palpomere than in any other *Nebria* species, Ledoux and Roux (2005) included it as a subgenus in their treatment of *Nebria*.

Once again, our analyses strongly support genus *Nebria* including *Eurynebria* as monophyletic (Chart 1, line 26) and a *Nebria* clade excluding *Eurynebria* as strongly contra-indicated (see further discussion below and Chart 1, line 31). We conclude that this taxon does not warrant generic status and should be classified within genus *Nebria*.

### Phylogeny of *Nebria*

As noted above, the evidence for the monophyly of *Nebria*, including *Archastes*, *Oreonebria*, and *Eurynebria* while excluding *Nippononebria* and *Vancouveria*, is strong. All concatenated analyses and the 28S analysis yielded MLB support values ≥ 97 (Chart 1, line 26). The Topo analysis also provides solid support (MLB value = 89) for this clade, and it appears in ML trees from five of the remaining six single-gene analyses (Suppl. material 2: Figs S6–S12) but with only weak support. Results from the COIBC analysis, which generated the only tree not showing this clade, differed only in having *Bembidion antiquum* from the outgroup included within the *Nebria* clade, a relationship not suggested by any of the other results. This *Nebria* clade is also supported by two unique bases (Suppl. material 1: Table S1).

Ledoux and Roux (2005: 75, “Graphique 2”) provided a tree illustrating proposed phylogenetic relations and classification of subgenera of *Nebria* based on their analyses of morphological data. Their analyses included a custom-designed, distance-based clustering method, as well as unspecified analyses using PHYLIP (Felsenstein 1989). Their clustering method was also designed to determine boundaries of taxa, including boundaries of subgenera, based upon a distance-based threshold. Their classification method found 12 distinct groups of subgenera, four of which included a single subgenus, the remainder including from two to four subgenera. They arranged these subgeneric groups into two larger assemblages, the “Vetanebri” and “Notanebri”. The main distinguishing features of these assemblages were as follows. Most Vetanebri male genitalia have a well-developed sagittal aileron at the base of the median lobe and females have a long, spiraled or convoluted spermathecal duct, or one that is abruptly expanded basally near its insertion on the bursa copulatrix. In contrast, most Notanebri males lack or have only a vestigial sagittal aileron and females typically have short and simple (not spiraled or convoluted) spermathecal ducts. Ledoux and Roux considered the absence of a sagittal aileron and presence of a long, spiraled or convoluted spermathecal duct as apotypic states. These distinguishing features explain why they included *Nippononebria* and *Vancouveria* in their Vetanebri. Indeed, those two clades have males with a well-developed sagittal aileron and females with very long, slender and convoluted spermathecal ducts. However, the fact that these features are shared also with *Leistus* males and females, respectively, suggests that both features may be symplesiomorphies within the Nebriini. This would mean that Ledoux and Roux’s Notanebri is supported as monophyletic by two synapomorphies while their Vetanebri is based mainly on two likely symplesiomorphies.

Results of our analyses of molecular data also support several large clades within *Nebria*, most including multiple subgenera. Following the system of informal hierarchic ranks between genus and subgenus used by Maddison (2012) for *Bembidion* Latreille, 1802, we call these clades “complexes”, with some of them subdivided into “subcomplexes” of subgenera. Major clades that include one or more complexes we call “series”. Our reconstruction of phylogenetic relationships within *Nebria* shows the genus divided into four series (Fig. 4A).

The first of these is the *Oreonebria* Series (Fig. 4B), which corresponds in many ways to Ledoux and Roux’s Vetanebri. However, it differs from the latter in key details. It does not include *Nippononebria* or *Vancouveria* but does include *Archastes* and a group of species that previously have been ascribed to *Epinebriola* Daniel & Daniel, 1904. This clade is strongly supported in both 8G analyses, the Nuc G analysis, and the 28S analysis (Chart 1, line 32). There is also moderate support from the Topo analysis and weak support from the NPC G and COIPJ analyses. One unique base (Suppl. material 1: Table S1) and one amino acid (Suppl. material 1: Table S2) unique to this clade among *Nebria* also support this clade. We find no consistent alternative pattern of relationships for members of this clade seen among the ML trees in which the clade does not appear (Suppl. material 2: Figs S4, S6, S7, S9, S10, S12). However, ML trees from the Mito G, CAD2, PEPCK, and *wg* analyses (Suppl. material 2: Figs S4, S9, S10, S12) show the *Oreonebria* Series as a grade rather than a clade, but each with different relationships shown among the taxa included in the grade.

The *Oreonebria* Series is an endemic Eurasian lineage that includes more than 180 described species-group taxa. This clade occupies a disjunct geographical distribution comprising the Alps mountain system of Europe in the west and central Asia to far eastern Asia, including Japan and Taiwan, in the east. It includes two groups of subgenera, the *Eonebria* and *Oreonebria* Complexes (Fig. 4B). Homoplasy is so widespread in key characters among *Nebria* in general and this clade in particular that we cannot cite any synapotypic features shared by all clade members. The main morphological characters that Ledoux and Roux (2005) cited as characteristic of this clade are either inconsistent or not unique to it. For example, males of this clade were characterized as having a sagittal aileron at the base of the median lobe of the genitalia, but males of some subgenera or species groups in this series do not have an evident sagittal aileron. Similarly, females of this clade were said to have long and helical spermathecal ducts or ducts with the proximal portion thickened and abruptly narrowed distally. This is correct as far as we have observed, but long and convoluted ducts or proximally thickened spermathecal ducts are found also in females of many Notanebri taxa, so this feature does not distinguish this clade from the latter.

The remaining three series of *Nebria* are strongly supported as a monophyletic group in both 8GML and 8G B analyses (Fig. 5A), as well as in the Nuc G, NPC G, and 28S analyses (Chart 1, line 44). The Mito G, COIPJ, CAD2PEPCK and *wg* analyses also provide independent but weak support. This clade is also supported by three bases unique in the entire dataset, one additional base unique among Nebriitae and two unique base insertions in 28S (Suppl. material 1: Tables S1, S3). Strong support for a sister group relationship between this clade and the *Oreonebria* Series (Fig. 4A) is shown in trees from the 8GML, 8G B, Nuc G, and NPC G concatenated analyses and the 28S analysis. This is a large clade, including more than 460 described species-group taxa, with a Holarctic distribution. As for the *Oreonebria* Series, we cannot cite any morphological feature unique to all members of this clade.

The Nebriola Series, which includes only subgenus Nebriola Daniel, 1903, is a small group comprised of only 19 described species-group taxa. Members of all described species are small and flightless due to atrophied hindwings. Their combined geographical distribution is confined to mountain systems of western Europe, from the Pyrenees Mountains of northern Spain and southern France northeast through the Alps to the Jura Mountains of eastern France and Switzerland and the Black Forest of southern Germany. Ledoux and Roux (2005) included *Nebriola* in their “Boreonebrides” among their Notanebri and cited the main morphological features characterizing members of this subgenus. Most distinctive among these are (1) males with protarsomeres 1 to 4 dilated; (2) male mesotarsomeres (and to a less extent metatarsomeres) 2 to 4 dilated, ca. as wide as long; and (3) females with the spermathecal duct long and convoluted and thickened proximally. Our results show the monophyly of subgenus Nebriola strongly supported (MLB values ≥ 98) in all concatenated and single-gene analysis except that for PEPCK (in which MLB support is a modest 58) (Chart 1, line 45). The clade is also supported by three unique amino acids, six unique bases and three unique insertions in 28S (Suppl. material 1: Tables S1–S3).

The *Nebria* Series is the most diverse and widespread group in the genus. More than 320 species-group taxa have been described, and the geographical range of the group encompasses most of the Holarctic Region and the northern edge of the Oriental Region in Asia. The group includes two main groups of subgenera, the *Boreonebria* and *Nebria* Complexes, and the latter includes three subgeneric subgroups, the *Nebria*, *Epinebriola* and *Eunebria* Subcomplexes (Fig. 4C). This series is equivalent to Ledoux and Roux’s (2005) Notanebri with the exclusion of subgenera *Nebriola*, *Reductonebria*, *Catonebria* and the Nearctic species included in their *Nakanebria* Ledoux & Roux, 2005. Monophyly of this group, thus comprised, is well supported by the 8GML, 8G B, and Nuc G concatenated-gene analyses, as well as by the 28S and PEPCK analyses (but the latter only weakly) (Chart 1, line 49). It is also supported by two unique bases (Suppl. material 1: Table S1). Modest support for a contradictory clade including *Nebriola* within the *Nebria* Series and excluding *Boreonebria* and the Palearctic *Nakanebria* species in our sample is provided by the NPC G and Topo analyses (Suppl. material 2: Figs S3 and S11, respectively). Results from the mitochondrial gene analyses, whether in single- or concatenated- gene analyses, show no consistent pattern of relationship among the included taxa. We conclude that the evidence for the monophyly of this group outweighs the evidence against it. However, we recognize that our sample includes only ca. 20% of described species-group taxa, so additional taxon sampling for molecular data is needed for this clade.

The *Catonebria* Series is a moderately diverse group with 104 described species, representing a majority of Nearctic *Nebria* species. It has a Holarctic distribution with a geographical range extending from central Asia east to eastern North America. It includes two subgeneric groups, the *Reductonebria* and *Catonebria* Complexes (Fig. 4D). Unlike any of the other complexes in genus *Nebria*, these two are more diverse in the Nearctic than in the Palearctic Region. This group corresponds to Ledoux and Roux’s (2005) “Serinebrides”, except that we exclude the Palearctic species of *Nakanebria* from it. The clade is supported by all our concatenated- and single-gene analyses (Chart 1, line 94) as well as by seven unique bases (Suppl. material 1: Table S1) and a two-base deletion in 28S unique among *Nebria* (Suppl. material 1: Table S3). With 95% of the species-group taxa described for this clade included in our sample, we have high confidence in these results.

Although our results provide evidence that the *Nebriola*, *Nebria*, and *Catonebria* Series together form a clade and that each individually is monophyletic, relationships among them are not yet as clear. Trees from both the 8GML (Fig. 4A) and 8G B (Fig. 5A) show a clade including the *Nebria* and *Catonebria* Series as sister to *Nebriola*, with moderate and strong support, respectively (Chart 1, line 46). The Nuc G, CAD2, and PEPCK analyses also support this clade, but there is no strong support from any single-gene analysis, and the NPC G, 28S, and Topo results provide MLB support ≥ 50 against this clade. Results from the 28S analysis show support (MLB value = 86) for a contradictory clade including *Nebriola* and the *Nebria* Series (Suppl. material 2: Fig. S5), but this clade has no other support (Chart 1, line 47). Although results from the NPC G concatenated analysis show support against the *Nebria* + *Catonebria* clade (MLB value = -57) and the ML tree from that analysis (Suppl. material 2: Fig. S3) shows a clade including *Nebriola* and the *Catonebria* Series, no single-gene or concatenated analysis shows much support for it (Chart 1, line 48). Consequently, our evidence suggests that a clade including the *Nebria* and *Catonebria* Series is likely sister to the *Nebriola* clade, but additional molecular sampling of species from both *Nebriola* and the *Nebria* Series would further test this hypothesis.

### *Oreonebria* Series: *Eonebria* Complex

The group of subgenera that we refer to as the *Eonebria* Complex (Figs 4B, 5B) is similar to Ledoux and Roux’s (2005) “Eonebrides” in that it includes the subgenera *Eonebria* Semenov & Znojko, 1928 and *Sadonebria* Ledoux & Roux, 2005. However, it also includes a group of species previously classified in subgenus Epinebriola. We recognize this last group of species as a distinct subgenus, *Parepinebriola*, which is described below (see the Taxonomy section). Together, these three subgenera represent an endemic Palearctic lineage that includes over 90 species with a combined geographical range extended from the southern margin of the Tibetan Plateau to Japan. Support for this clade is provided by all the single-gene analyses except for PEPCK and *wg*, and by all the concatenated analyses (Chart 1, line 33).

The most diverse subgenus in this clade is *Eonebria* (Fig. 3A) with 77 described species-group taxa. Most of these species occupy the eastern part of the Tibet-Qinghai Plateau east to western Hubei Province, and from eastern Qinghai in the north to northern Yunnan Province in the south. However, a group of three species, including the type species, *Nebria komarovi* Semenov & Znojko, 1928, occurs in the Russian Far East and North Korea and no species are known from the intervening area. This represents a significant disjunction in the range of the subgenus. A monophyletic *Eonebri*a, with *N. komarovi* most basal among species included in our sample, is well supported by analyses of the mitochondrial genes both individually and concatenated, and independently by PEPCK and *wg* results (Chart 1, line 37). Strong support is also provided by the concatenated 8GML analysis. However, the trees from the 8G B and 28S analyses (Suppl. material 2: Figs S1, S5) show *N. komarovi* as more distantly related to the other *Eonebria* species in our sample than are the species of *Nakanebria*. Additional molecular sampling of species from each of the vicariant regions might either further support a monophyletic *Eonebria* or reveal a more significant phylogenetic split between the subfaunas.

Diversity within subgenus Sadonebria has increased from the three species recognized by Ledoux and Roux (2005) to 15, based on morphometric analyses of pronota, elytra and male genitalia and detailed examination of the internal sac of the median lobe of males by Sasakawa (2006, 2008, 2009, 2010, 2016; Sasakawa and Toki 2011). All but one of these species occur in Japan. The lone exception is *Nebria niitakana* Kano, 1930 from Taiwan. Only one of the Japanese species, *Nebria chinensis* Bates, 1872, also occurs on the Asian mainland as far west as eastern Sichuan and Shaanxi Provinces. Based on the four species in our sample, the monophyly of *Sadonebria* is well supported by all our analyses (all MLB or BPP values ≥ 93) (Chart 1, line 36).

One of the most unexpected results of our study was the discovery of the group of five species represented in our sample, including *Nebria delicata* Huber & Schmidt, 2017 and *Nebria retingensis* Huber & Schmidt, 2017 and three undescribed species (Figs 4B, 5B), which we assign to a new subgenus, *Parepinebriola* (for description see Taxonomy section). The geographical range of the known species of this group occupies a very limited area extending from the Central Trans-Himalaya in southern Tibet eastward to the Gaoligong Shan of northwestern Yunnan. This group is well supported as monophyletic in all our single-gene and concatenated analyses (Chart 1, line 34). All but one of these species had been identified as members of subgenus Epinebriola [the lead author had initially identified the fifth sample as an *Eonebria* species]. We had DNA from only eight (28%) of the 29 described species of *Epinebriola* available for our study, including the two that our results suggest are not part of an *Epinebriola* clade. Consequently, we do not yet know if other species currently assigned to *Epinebriola* actually belong to the *Parepinebriola* clade. Only three species among those remaining in *Epinebriola* occur near or within the known geographical range of *Parepinebriola* species. Two of them, *Nebria businskyorum* Ledoux & Roux, 1997 and *Nebria laevistriata* Ledoux & Roux, 1998 represented in our taxon sample are clearly part of the *Epinebriola* clade and form a distinct subclade within that taxon (see further discussion below under that subgenus). The third, *Nebria zayula* Andrewes, 1936 occurs near the eastern end of the range of *Parepinebriola* and remains a candidate for this taxon. As might be expected from the past taxonomic intermixing, morphological characters that distinguish members of *Parepinebriola* species from those of *Epinebriola* species are few and mainly involve internal features (see the Taxonomy section for discussion of these characters).

The monophyly of a group including *Sadonebria* and *Eonebria* as sister to *Parepinebriola* (Fig. 5B) is well supported by all the concatenated analyses and by the 28S, 16S-ND1 and Topo single-gene analyses (Chart 1, line 35) (Suppl. material 2: Figs S5, S6, S11).

### *Oreonebria* Series: *Oreonebria* Complex

The *Oreonebria* Complex of subgenera (Figs 4B, 5B) is similar to Ledoux and Roux’s Vetanebri but with the addition of *Archastes* and the exclusion of their Nippononebrides and Eonebrides. We recognize five subgenera within the group, which include 90 described species-group taxa. The geographical range of the group is widely disjunct, with subgenus Oreonebria restricted to the Alps mountain system of western Europe and the remaining subgenera found in eastcentral to far eastern Asia. This group is well supported as monophyletic by the 8GML, 8G B, and Nuc G concatenated analyses and the 28S analysis (Chart 1, line 38) and, to a lesser degree, by the COIBC, COIPJ, and PEPCK analyses (Suppl. material 2: Figs S7, S8, S10).

Within the complex, two main groups of subgenera are apparent. The first is a clade including *Falcinebria* Ledoux & Roux, 2005 and *Epispadias* Ledoux & Roux, 2005. It is well supported (MBL and BPP values ≥ 96) in all our analyses except for *wg* (Chart 1, line 39), as well as by three unique amino acids (Suppl. material 1: Table S2) and a unique base insertion in 28S (Suppl. material 1: Table S3). *Falcinebria* currently includes 15 species, with seven of these only recently distinguished by Sasakawa (2020) using the same character systems as noted above for *Sadonebria*. It is a group of morphologically very similar species. The geographical range of this subgenus includes Japan (11 species), Taiwan (two species), and the Asian mainland (one species known from Guangxi and one from Sichuan Provinces in China). The two species represented in our sample (one from Taiwan and one from Japan) form a monophyletic group based on evidence from every one of our analyses (Chart 1, line 40). *Epispadias* was previously known from a single species, *Nebria janschneideri* Ledoux & Roux, 1999, based on two specimens from the Jinfo Shan in southeast Sichuan Province (now part of Chongqing Municipality). The species in our sample is undescribed but clearly related to *N. janschneideri* because males of both species share a genitalic median lobe that is completely membranous dorsally except at the basal bulb, a feature unique among nebriites. They also share exceptionally low-elevation habitats for *Nebria* in the regions where they have been found.

The other major group of subgenera in this complex includes *Orientonebria* Shilenkov, 1975, *Archastes* and *Oreonebria* (Fig. 4B). *Orientonebria* includes a single species, *Nebria coreica* Solsky, 1875 (Fig. 3B), which is restricted to central Japan and the adjacent Asian mainland in southern Primorsky Krai in Russia, Jilin Province in China and North and South Korea. The diversity and overall distributions of *Archastes* and *Oreonebria* already have been discussed (see above). This clade is well supported in all our analyses except the 16S-ND1 analysis (Fig. 5B) (Chart 1, line 41), as well as by one base unique for the entire taxon sample and one unique among nebriites (Suppl. material 1: Tables S1). In addition, a clade including *Orientonebria* and *Archastes* is well supported as sister to *Oreonebria* by all our analyses (Chart 1, line 42).

Ledoux and Roux (2005) followed Jeannel in treating *Nebria gagates* Bonelli, 1810 as a distinct, monobasic subgenus, *Nebriorites* Jeannel, 1941. They also treated *Nebria bremii* Germar, 1831 as a separate subgenus, *Germarina* Jeanne, 1985 (= *Marggia* Huber, 2014). A second species, *Nebria bluemlisalpicola* Szallies & Huber, 2014, has been described in this subgenus. Results from every one of our concatenated and single-gene analyses support a monophyletic *Oreonebria* including both *N. gagates* and *N. bremii* (Chart 1, line 43, Figs 4B, 5B). In none of the trees from these analyses is either of these species shown anywhere but embedded within *Oreonebria* (Suppl. material 2: Figs S1 to S10). Within *Oreonebria*, our results support Ledoux and Roux’s division of the subgenus into the *austriaca* and *castanea* species groups, although we did not have all of the species available for our analyses. In addition, ML trees from all our analyses except the 28S and 16S-ND1 analyses show *N. gagates* as a member of the *austriaca* group clade and *N. bremii* as part of the *castanea* group clade (Fig. 4B).

### *Nebria* Series: *Boreonebria* Complex

The *Boreonebria* Complex is comprised of two subgenera, *Boreonebria* and *Nakanebria*. This group includes 66 species-group taxa with a combined geographical range that covers most of the Holarctic Region. It differs from Ledoux and Roux’s (2005) “Boreonebrides” in that *Nebriola* is excluded from the group and the Palearctic species of *Nakanebria* are included in it. All females of this group have the spermathecal duct inserted on the ventral face of the spermathecal chamber of the bursa copulatrix, a feature not shared with females of any other subgenus of *Nebria*. This clade is well supported in all our concatenated analyses and in all but one (Topo) single-gene analyses (Chart 1, line 50; Figs 4C, 5C). These two subgenera also share one unique amino acid and six unique bases (Suppl. material 1: Tables S1, S2).

Ledoux and Roux (2005) included *Nakanebria* with *Reductonebria* and *Catonebria* in their “Serinebrides”, but whereas the Nearctic species in their *Nakanebria* appear to be closely related to the latter two subgenera (see below), the Palearctic species do not. Because the type species of this subgenus, *Nebria kurosawai* Nakane, 1960, is part of the Palearctic fauna, the subgeneric name remains with the Palearctic species as part of the *Boreonebria* Complex. The group includes six species-group taxa, four found only on the island of Hokkaido in northern Japan, one in the southern Kuril Islands, and one on the Asian mainland in northern North Korea. While our sample includes only one of these taxa (*Nebria shiretokoana* Nakane, 1960), we have examined specimens of all the included species except *Nebria kumgangi* Shilenkov, 1983 and can confirm close relationships based on morphology in general and the ventral insertion of the spermathecal duct on the bursa in particular.

Subgenus Boreonebria includes the 60 remaining species of this complex. Their combined geographical range extends across the Holarctic region, from Greenland and Iceland eastward across Eurasia, Beringia and across North America from Alaska to Ellesmere Island and the Island of Newfoundland. In Eurasia, they range from above the Arctic Circle to southern Europe (but not northern Africa), the Tian Shan, Altai, and Qilian mountain systems of central Asia, northern China, North Korea, and northern Japan. In North America, they range south into the northern Sierra Nevada in California, the southern Rocky Mountains in northern New Mexico, and into the southern Appalachian Mountains of Tennessee and North Carolina. Ledoux and Roux (2005) treated Pseudonebriola Ledoux & Roux, 1989 as a distinct subgenus closely related to Boreonebria. Our results strongly support a clade including *Pseudonebriola* nested within *Boreonebria* (Chart 1, line 51; Figs 4C, 5C) but not sister to it. This relationship is seen in ML trees from all concatenated gene analyses and all but one (Topo) single-gene analyses Suppl. material 2: Figs S2 to S12). At the same time, a clade including all *Boreonebria* species but excluding *Pseudonebriola* species is strongly contra-indicated (Chart 1, line 52), as is a clade including all *Pseudonebriola* species and excluding all *Boreonebria* species (Chart 1, line 53). This evidence suggests that either *Pseudonebriola* should be considered as a junior synonym of *Boreonebria* or the latter should be split into two or more subgenera. Even within *Boreonebria*, *Pseudonebriola* is shown either as a grade or as polyphyletic rather than a clade in all ML trees except the NPC G tree (Suppl. material 2: Fig. S3). Consequently, we conclude that these names should be synonymized.

Within *Boreonebria*, several well-supported clades can be recognized (Fig. 5C). First and most basal is the *hudsonica* species group (sensu Ledoux and Roux 2005). This group of four species is endemic to North America, with two species each in the western and eastern parts of the continent. This clade is supported by all concatenated and single-gene analyses except for *wg* (Chart 1, line 54). A clade including the remaining *Boreonebria* species as sister to the *hudsonica* group is also supported by the concatenated analyses and all single-gene analyses except for 28S and Topo (Chart 1, line 55). Within this latter clade, the *nivalis* group, which includes some but not all species from several of the species groups recognized by Ledoux and Roux (2005), is seen as sister to the remainder of the subgenus. Members of all of these species share an antennal scape that is more or less elongate and sinuate. Their combined east/west geographical range is that of the subgenus, but they occupy areas north of 50°N latitude except in Mongolia, the Asian Far East and the northernmost Appalachian Mountains and Island of Newfoundland in eastern North America. This group includes at least eleven species, one of which (*Nebria nivalis* (Paykull), 1798) is Holarctic, one (*Nebria gaspesiana* Kavanaugh, 1979) occurs only in northeastern North America, and the remainder are Eurasian. Two of the species in our taxon sample are from Mongolia and are not yet described. This clade is well supported by all our concatenated and single-gene analyses except 28S (Chart 1, line 56).

Our results suggest that sister to the *nivalis* group is a clade that includes all the species currently assigned to subgenus Pseudonebriola and the remaining *Boreonebria* species, which we call the *gyllenhali* group Fig. 4C. This clade is well supported by all the concatenated analyses except the Mito G analysis, as well as by the CAD2 and *wg* analyses (Chart 1, line 57). Modest additional support is shown by the COIBC and COIPJ results. Species currently assigned to *Pseudonebriola* appear to represent either a grade or a polyphyletic assemblage, which, as a group, occupy mountain ranges in the region in central Asia extending from the western end of the Tian Shan just south of Issyk Lake northward and eastward to the southern end of Lake Baikal in southern Buryatia, Russia (Huber and Schnitter 2020). One species, *Nebria mingyii* Ledoux & Roux, 2014, is known from the Qilian Shan of Qinghai Province, China, more than 1000 km south and east of the range of any other species in the group. Fifteen species have been included in the group and two additional undescribed species (both in our taxon sample) appear to be closely related to them. Ledoux and Roux (2005) recognized five species groups within *Pseudonebriola*. In our sample, which included five (33%) of the described species and the two undescribed species, two moderately well supported clades are represented. These do not appear to be sister taxa. The first includes two species, *Nebria tekesensis* Ledoux & Roux, 2005 and *Nebria murzini* Ledoux & Roux, 2000, which Ledoux and Roux had placed in two different species groups. This clade is supported by all the concatenated analyses and by three nuclear and two mitochondrial single-gene analyses (Chart 1, line 58). It appears to be sister to a clade including the other group of *Pseudonebriola* species and the *gyllenhali* group of *Boreonebria*. This latter clade is supported by the 8GML, 8G B, and Mito G concatenated analyses, as well as by the COIBC, COIPJ, and, to a lesser extent, the CAD2 analyses (Chart 1, line 59). The second *Pseudonebriola* group, which we are calling the *sajanica* group, is probably equivalent to Ledoux and Roux’s species group of the same, but we were not able to include *Nebria stanislavi* Dudko & Matalin, 2002 in our taxon sample. This group also is supported as monophyletic by all concatenated analyses and by COIBC and COIPJ (mitochondrial) and CAD2 and *wg* (nuclear protein-coding) single-gene analyses (Chart 1, line 60). These two groups of species appear to occupy opposite ends of the range of the group as a whole, with the first group in the southwestern portion of the range and the *sajanica* group occupying the northeastern portion. It should be interesting to see how the species not included in our sample assort between these two clades or if additional clades are found with wider molecular sampling.

Our *gyllenhali* group of *Boreonebria* is only partially equivalent to that of Ledoux and Roux’s (2005) species group of the same name in that we include *Nebria frigida* Sahlberg, 1844 and exclude several *nivalis* group species that Ledoux and Roux included. This group is supported as monophyletic by the 8GML, 8G B, Nuc G, and NPC G analyses and only modestly by one single-gene analysis (CAD2) (Chart 1, line 61). Nonetheless, it is the only pattern of relationship among these taxa that is supported with any consistency. Relationships within this group are only partially resolved with our findings. Kavanaugh (1978, 1979) initially considered *Nebria castanipes* Kirby, 1837, *Nebria lassenensis* Kavanaugh, 1979 and *Nebria lindrothi* Kavanaugh, 1979 as subspecies of a Holarctic *Nebria gyllenhali* (Schönherr, 1806). Results from our 8GML, 8G B and Mito G concatenated analyses and COIBC and COIPJ analyses support a Nearctic clade, including those three species and *Nebria crassicornis* Van Dyke, 1925 and *Nebria intermedia* Van Dyke, 1949, but not including *N. gyllenhali* (Chart 1, line 63). Conversely, a clade including *N. gyllenhali* with *N. castanipes*, *N. lassenensis*, and *N. lindrothi* but excluding *N. crassicornis* and *N. intermedia* (Chart 1, line 62) is contra-indicated. This suggests that *N. gyllenhali* is an endemic Palearctic species, not most closely related to any of the Nearctic taxa.

We have not had the opportunity to include in our study several of the species of *Boreonebria* that Ledoux and Roux included in this group from southern and eastern Europe, China (including Tibet), Kazakhstan, or Afghanistan. However, those authors provided illustrations of the female bursa copulatrix and spermathecal duct and reservoir for several of these, all but one of which appear to have the spermathecal duct inserted ventrally or apicoventrally on the bursa. The lone exception is *Nebria klapperichi* Bänninger, 1956, which appears to have the duct inserted mid-dorsally on the bursa (Ledoux and Roux 2005, pg. 116, fig. 53). This feature, coupled with the fact that the area where this species occurs (in Afghanistan) is more than 1000 km southeast of the range of the nearest confirmed members of this group, suggests that *N. klapperichi* is not a member of *Boreonebria* or the *Boreonebria* Complex.

### *Nebria* Series: *Nebria* Complex

The group of taxa that we recognize as the *Nebria* Complex (Fig. 4C) includes eleven (> 40%) of the subgenera recognized by Ledoux and Roux (2005) and 286 species-group taxa. It is the most diverse subgeneric complex within *Nebria* and restricted to the Palearctic Region, except for a few accidental introductions of *Nebria brevicollis* (Fabricius, 1792) into North America. This clade is supported in all our concatenated analyses and in the 28S, CAD2, PEPCK and Topo analyses (Chart 1, line 64). As noted above, our results show that this group is comprised of three distinct groups of subgenera that we recognize as the *Nebria*, *Epinebriola* and *Eunebria* Subcomplexes, respectively. The large and diverse *Nebria* Subcomplex is strongly supported as monophyletic by results from all concatenated and single-gene analyses except for COIPJ (Chart 1, line 65). Sister to the *Nebria* Subcomplex is a group including the *Epinebriola* and *Eunebria* Subcomplexes. Monophyly of this group is well supported by the 8GML, 8G B, and Nuc G concatenated analyses and the 28S and Topo analyses and also (but weakly) by the Mito G analysis (Chart 1, line 71) and there is no other consistent pattern of relationship supported by our results.

#### *Nebria* Subcomplex

This group of subgenera is equivalent to the “Nebrides” of Ledoux and Roux (2005) except that we also include here subgenus Tyrrhenia Ledoux & Roux, 2005, which they included in their “Eunebrides”. Currently, nearly 160 species-group taxa are arrayed among four subgenera: *Nebria* s. str., *Alpaeonebria* Csiki, 1946, *Spelaeonebria* Peyerimhoff, 1911, and *Tyrrhenia*. This group is mainly a European lineage, with highest diversity in southern Europe, northern Turkey, and the Caucasian mountain region. The geographical area of the group covers all of Europe, extreme North Africa, the Mediterranean fringe of the Middle East and Asia Minor to the southern end of the Caspian Sea, and no species (except for *Nebria brevicollis* in the north), occurs east of northcentral Iran. This group is the least well represented in our taxon sample with only ten (ca. 6%) of the described taxa included, but all of the subgenera are represented.

*Tyrrhenia* includes 18 species-group taxa with a combined geographical range extending from Portugal and Morocco across southern Europe and northern Africa to southeastern Turkey. Although we had only two species of this subgenus represented in our sample, they form a well- supported clade in all our analyses (Chart 1, line 66). Two base insertions and two deletions in 28S are unique to the clade (Suppl. material 1: Table S3). They also appear in all but one (COIPJ) of our ML trees as sister to a clade including other members of this subcomplex (Figs 4C, 5C; Suppl. material 2: Figs S1–S12). This latter clade, the Nebrides of Ledoux and Roux, is also well supported by our results, including those from all the concatenated and single-gene analyses except for PEPCK (for which we obtained insufficient data) (Chart 1, line 67). One unique base (Suppl. material 1: Table S1) and one unique three-base insertion in 28S (Suppl. material 1: Table S3) also support this clade.

*Spelaeonebria* includes a single species, *Nebria nudicollis* Peyerimhoff, 1911, restricted to the Djurdjura Mountains of northern Algeria, where these large and elegant beetles (3C) live in limestone caves at high elevation. *Alpaeonebria*, as currently comprised, includes 33 species-group taxa arrayed in nine species groups (Ledoux and Roux 2005). This subgenus occupies two separate regions. The first is an area extending from the Carpathian Mountains in the northeast west through the Alps of Austria, Switzerland, and northern Italy (but not into France or Germany) and south through the Balkan countries to northern Albania, Macedonia, and western Bulgaria. The second region includes the mountains of northern Africa in Algeria and Morocco and Tenerife and Grand Canary in the Canary Islands. The subgenus is absent from the Iberian, Italian, and southern Balkan peninsulas and the islands of Mediterranean. Our taxon sample was insufficient to test whether or not *Alpaeonebria* is a clade.

Ledoux and Roux (2005) treated Alpaeus Bonelli, 1810 as a synonym of subgenus Nebria because they found the morphological features traditionally used to distinguish between them unreliable (see also Ledoux and Roux 1990). As presently constituted, the nominate subgenus includes more than 130 species-group taxa with a combined geographical range extended from northern Africa through all of Europe except the Arctic, eastward to the Urals in the north and to the Middle East and the southern edge of the Caspian Sea in Asia Minor in the south. Ledoux and Roux recognized fifteen different species groups within this subgenus.

The number of taxa represented in our sample for this group is low in relation to its high diversity. While this prevents us from drawing firm conclusions about relationships within it, our results allow us to clarify a few issues. First, our representatives of both *Alpaeonebria* (*Nebria germarii* Heer, 1837) and *Spelaeonebria* (*N. nudicollis*) are nested within a *Nebria* clade in ML trees from all concatenated and single-gene analyses except for Topo (Suppl. material 2: Fig. S11), in which *N. germarii* is shown as sister to a group including the *Nebria* s. str. species and *N. nudicollis*. The relationship suggested by the Topo result is not supported by evidence from most other genes (Chart 1, line 69). A clade including *Nebria* s. str. species but excluding either *N. nudicollis* alone (Chart 1, line 68) or along with *N. germari* (Chart 1, line 70) is strongly contra-indicated by our results. This suggests that treating *Spelaeonebria* and *Alpaeonebria* as separate subgenera renders *Nebria* s. str. as a paraphyletic or even a polyphyletic assemblage. In most of the trees, *Nebria turcica* Chaudoir, 1843 is shown as sister to the other species in our sample (Fig. 4C; Suppl. material 2: Figs S1–S6, S8), but in the ML trees from the COIBC and CAD2 analyses (Suppl. material 2: Figs S7, S9), it is shown as sister to *N. germarii* (the *Alpaeonebria* representative in our sample). *Nebria nudicollis* is shown either as sister to *Nebria tibialis* Bonelli, 1810) and nested within a European clade of *Nebria* species (Fig. 4C) or as sister to that clade (Suppl. material 2: Figs S4, S10, S12). Peyerimhoff (1911) suggested a close relationship between *Spelaeonebria* and *Nebria exul* Peyerimhoff, 1910, which was included by Ledoux and Roux (2005) in *Alpaeonebria*. Both *Nebria turcica* and *N. tibialis* were previously included in *Alpaeus* (Farkač and Janata 2003). Clearly, there is more complexity to this clade than we can resolve with our limited sample. Our choice at this point is to either synonymize both *Spelaeonebria* and *Alpaeonebria* with *Nebria* s. str. and create an even larger single taxon or accept the current status, which includes a para- or polyphyletic *Nebria* s. str. We choose the latter option pending much broader taxon sampling.

#### *Epinebriola* Subcomplex

This group is comprised of 46 described species-group taxa currently arrayed among four subgenera: *Epinebriola*, *Barbonebriola* Huber & Schmidt, 2017, *Patrobonebria* Bänninger, 1923 and *Paranebria* Jeannel, 1937. Ledoux and Roux (2005) included *Epinebriola* (including the species of *Barbonebriola* known to them) and *Patrobonebria* with *Psilonebria* Andrewes, 1923 in their “Patrobonebrides”, whereas *Paranebria* was part of their “Eunebrides” with *Tyrrhenia*, *Eunebria* Jeannel, 1937 and *Asionebria* Shilenkov, 1982. The monophyly of this group is well supported by all our concatenated and single-gene analyses (Chart 1, line 72). *Paranebria* includes only two species. *Nebria livida* (Linnaeus, 1758) ranges across Eurasia at latitudes from slightly south of the Arctic Circle to mid-latitudes, from the United Kingdom east to Japan but with large gaps in the record from western and central Asia. The second species, *Nebria macrogona* Bates, 1873, occurs only in Japan. Members of both of these species have full-sized hindwings and are likely capable of flight. *Patrobonebria* includes ten described and at least one undescribed species. This group is restricted to the western and southern margins of High Asia, with a combined geographical range extended from northwestern Afghanistan to ranges of the Hengduan Shan in western Yunnan Province, China. All except two of these species (*Nebria assidua* Huber & Schmidt, 2009 and *Nebria pertinax* Huber & Schmidt, 2009) have members with full-sized, functional hindwings. Huber and Schmidt (2017) distinguished members of *Barbonebriola* from those of *Epinebriola* mainly by the presence of laterally bulging, seta-bearing tubercules on the maxillary stipes (not seen in *Epinebriola* members). They recognized six species in this group, with a combined geographical range extended from extreme northern Pakistan narrowly along the Himalayan Mountain system to western Nepal. All of these species have vestigial hindwings. The remaining 28 species of this subcomplex are currently included in subgenus Epinebriola and all have vestigial hindwings. Males of the species that we have examined share a median lobe of their genitalia with well-developed basolateral lobes on the basal bulb and an apical orifice slightly to markedly deflected right. Females share a short spermathecal duct and medium-length spermathecal reservoir. The combined geographical range of this group is similar to that of *Patrobonebria* except that, in the eastern part of its range, on the eastern margin of the Tibet-Qinghai Plateau, it is also extended north to the Kunlun Mountains of extreme western Qinghai Province, China.

Our analyses of relationships within this clade have produced some unexpected findings. As already noted above, two described species (*N. delicata* and *N. retingensis*) previously included in *Epinebriola* have been shown instead to be part of the *Eonebria* Complex. A clade including them with members of the *Epinebriola* Subcomplex is contra-indicated by all of our results (Chart 1, line 73). *Patrobonebria* is well supported as a clade in all of our analyses (Chart 1, line 79) and *Paranebria* is supported as monophyletic (Chart 1, line 78) in all trees except the 28SML tree (Suppl. material 2: Fig. S5). However, both of these groups are nested within *Epinebriola* as currently comprised (Fig. 4C) in all trees except the 28S tree, which shows a clade including *Patrobonebria* and *N. macrogona* (but not *N. livida*) as sister to *Epinebriola* including *Nebria kagmara* Huber & Schmidt, 2017, our only representative of *Barbonebriola*. *Nebria kagmara* also is nested within *Epinebriola* in ML trees from all analyses. This evidence suggests that *Paranebria*, *Patrobonebria*, and *Barbonebriola* should be considered junior synonyms of *Epinebriola*, but we wanted to look at this group in greater detail to determine if it would be possible to conserve any of these names.

We only had access to a very old pinned specimen of *Nebria oxyptera* Daniel & Daniel, 1904, the type species of *Epinebriol*a, for DNA extraction. This may explain why we were unable to obtain sequence data from it for any of the nuclear protein-coding genes. Consequently, this important taxon is missing from several of our analyses and resulting ML trees. Nonetheless, in five of the eight ML trees in which it appears, it is shown as sister to the *Paranebria* species, forming a clade with them that is sister to *Patrobonebria* (Fig. 4C; Suppl. material 2: Figs S1, S2, S4, S8). The monophyly of *N. oxyptera* + *Paranebria* is supported in all the concatenated analyses in which data for *N. oxyptera* are included and in the single-gene analysis for COIPJ (Chart 1, line 77). In the tree for 16S-ND1 (Suppl. material 2: Fig. S6), *N. oxyptera* is seen as sister to a clade including *Paranebria* with *Patrobonebria*. In either case, the type species of *Epinebriola* is shown to be more closely related to *Paranebria* and *Patrobonebria* than to the other *Epinebriola* species in our sample. Consequently, we see no way to recognize either *Paranebria* or *Patrobonebria* as a separate subgenus without rendering *Epinebriola* paraphyletic, so the three should be synonymized. What is perhaps most unexpected here is the fact that taxa with full-sized hindwings are, in most trees, nested well within larger clades of taxa with reduced hindwings. This requires that the loss of functional wings has occurred repeatedly in this group and probably independently.

*Nebria kagmara* is not the most genetically distinct taxon within our *Epinebriola* sample. That distinction belongs to a clade including *Nebria businskyorum* Ledoux & Roux, 1997 and *Nebria laevistriata* Ledoux & Roux, 1998, two of the three species Ledoux and Roux (2005) included in their *pindarica* species group. This species pair is strongly supported as monophyletic in all our analyses (Chart 1, line 74) and as sister to all other members of the subcomplex in our taxon sample in all but the 28S and COIBC analyses (Fig. 4C) (Chart 1, line 75). A unique two-base insertion in 28S (Suppl. material 1: Table S3) also supports this clade. Another small clade recovered with strong support from all of our analyses (Chart 1, line 76) includes three species (*Nebria pseudorestias* Huber & Schmidt, 2017, *Nebria martensi* Huber & Schmidt, 2012 and *Nebria numburica* Huber & Schmidt, 2017), the *martensi* species group. Members of this clade share a unique three-amino acid insertion in the *wg* gene fragment (Suppl. material 1: Table S3). Different analyses suggest different relationships of this clade to the others in this lineage (Suppl. material 2: Figs S1–S12), but in most results, it occupies a middle level in the diversification of the subcomplex (Fig. 4C). Based on morphological data, it seems likely that most if not all of the other species of *Epinebriola* that were not included in our analyses are more closely related to this clade than to any of the others in the subcomplex. Although the six species included in *Barbonebriola* certainly share at least one outstanding morphological feature, maintaining them as more than a distinctive species group within *Epinebriola* would render the latter paraphyletic unless it is further subdivided, which we discourage based on data available at this time. Consequently, these two subgeneric names should also be synonymized (see proposed revised classification of Nebriitae below, Table 4).

**Table 4.** A revised classification of the supertribe Nebriitae.

Supertribe Nebriitae
Tribe Notiokasiini
[Genus *Notiokasis* Kavanaugh & Nègre, 1983]
Tribe Notiophilini
Genus *Notiophilus* Duméril, 1805
Tribe Opisthiini
Genus *Opisthius* Kirby, 1837
Genus *Paropisthius* Casey, 1920
Tribe Pelophilini
Genus *Pelophila* Dejean, 1821
Tribe Nebriini
[Genus *Archaeonebria* Kavanaugh & Schmidt, 2019 **Fossil**]
Genus *Nippononebria* Uéno, 1955 **Revised Status**
Subgenus Nippononebria Uéno, 1955
Subgenus Vancouveria Kavanaugh, 1995 **Revised Status**
Genus *Leistus* Frölich, 1799
Subgenus Nebrileistus Bänninger, 1925
Subgenus Sardoleistus Perrault, 1980
Subgenus Pogonophorus Latreille, 1802
[*Manticora* Panzer, 1803]
[*Oreobius* Daniel, 1903]
[*Chaetoleistus* Semenov, 1904]
*Eurinoleistus* Breit, 1914
Subgenus Evanoleistus Jedlička, 1965
Subgenus Leistus Frölich, 1799
[*Leistidius* Daniel, 1903]
[*Acroleistus* Reitter, 1905]
*Euleistus* Reitter, 1905
*Leistophorus* Reitter, 1905
*Neoleistus* Erwin, 1970
Genus *Nebria* Latreille, 1802
*Oreonebria* Series
*Eonebria* Complex
Subgenus Parepinebriola**New Subgenus**
Subgenus Sadonebria Ledoux & Roux, 2005
Subgenus Eonebria Semenov & Znojko, 1828
*Oreonebria* Complex
Subgenus Epispadius Ledoux & Roux, 1999
Subgenus Falcinebria Ledoux & Roux, 2005
Subgenus Orientonebria Shilenkov, 1975
Subgenus Archastes Jedlička, 1935 **New Status**
Subgenus Oreonebria Daniel, 1903
*Nebriorites* Jeannel, 1941 **New Synonymy**
*Germaria* Jeanne, 1972
*Germarina* Jeanne, 1985
*Marggia* Huber, 2014 **New Synonymy**
*Nebriola* Series
Subgenus Nebriola Daniel, 1903
*Nebria* Series
*Boreonebria* Complex
Subgenus Nakanebria Ledoux & Roux, 2005
Subgenus Boreonebria Jeannel, 1937
*Pseudonebriola* Ledoux & Roux, 1989 **New Synonymy**
*Nebria* Complex
*Nebria* Subcomplex
Subgenus Tyrrhenia Ledoux & Roux, 2005
Subgenus Nebria Latreille, 1802*
*Alpaeus* Bonelli, 1810
*Helobia* Stephens, 1828
*Harpazobia* Gistel, 1856
Subgenus Spelaeonebria Peyerimhoff, 1911
Subgenus Alpaeonebria Csiki, 1946
*Epinebriola* Subcomplex
Subgenus Epinebriola Daniel & Daniel, 1904
*Patrobonebria* Bänninger, 1923 **New Synonymy**
*Paranebria* Jeannel, 1937 **New Synonymy**
[*Himalayonebria* Ledoux, 1985]
*Barbonebriola* Huber & Schmidt, 2016 **New Synonymy**
*Eunebria* Subcomplex
Subgenus Psilonebria Andrewes, 1923
*Asionebria* Shilenkov, 1982 **New Synonymy**
Subgenus Eurynebria Ganglbauer, 1891
Subgenus Eunebria Jeannel, 1937
[*Tetungonebria* Shilenkov, 1982]
[*Sphodronebria* Sciaky & Pavesi, 1994]
*Catonebria* Series
*Reductonebria* Complex
Subgenus Insulanebria**New Subgenus**
Subgenus Reductonebria Shilenkov, 1975
Subgenus Erwinebria**New Subgenus**
*Catonebria* Complex
Subgenus Nivalonebria**New Subgenus**
Subgenus Neaptenonebria**New Subgenus**
Subgenus Palaptenonebria**New Subgenus**
Subgenus Catonebria Shilenkov, 1975
Incerta sedis
[Genus *Archileistobrius* Shilenkov & Kryzhanovskij, 1983]
[Genus *Ledouxnebria* Deuve, 1998 **Fossil**]

* As presently comprised, this subgenus is demonstrably para- or even polyphyletic; taxa in brackets [] not represented in our taxon sample.

#### *Eunebria* Subcomplex

This subcomplex includes 68 described species-group taxa presently arrayed in four subgenera: *Asionebria*, *Eunebria*, *Psilonebria* and *Eurynebria*. Ledoux and Roux (2005) included the first two along with *Tyrrhenia* and *Paranebria* in their Eunebrides and *Psilonebria* with *Patrobonebria* and *Epinebriola* in their Patrobonebrides. *Eurynebria* was assigned to its own monobasic group, the “Halonebrides”. This group ranges widely but discontinuously across the Palearctic Region in mid- and southern latitudes, from extreme northern Africa and the United Kingdom east to Japan and Taiwan, and along the southern margin of High Asia. Monophyly of the group (Fig. 4C) is supported by results from all our concatenated analyses as well as from 28S and CAD2 analyses (Chart 1, line 80). This clade also appears in ML trees from the PEPCK and *wg* analyses (Suppl. material 2: Figs S10, S12).

Relationships among the taxa included in our analyses proved to be somewhat unexpected. The group is clearly divided into two well-supported clades (Fig. 4C). The first includes all the species of *Asionebria* and *Psilonebria* in our sample, but also three species from Ledoux and Roux’s (2005)*przewalskii* group of *Eunebria*. A clade retaining the *przewalskii* group with the other species of *Eunebria* in our sample is strongly contra-indicated by results from all analyses (Chart 1, line 81). This clade probably includes all 20 of the described species-group taxa in these three groups, including those species and subspecies in the *przewalskii* species group not included in our sample; however, confirming this will require additional DNA sampling. In addition, several undescribed taxa are also included in this group. The combined geographical range of this group includes the southern and eastern parts of the Tibetan Plateau north of the Himalayan Mountains, extending from the central parts of the Tibetan Himalaya and Trans-Himalaya in southern Tibet to the Qilian Mountain system north and northeast of Qinghai Lake in eastern Qinghai and western Gansu Provinces, China. *Psilonebria* as presently comprised occupies only the southwesternmost part of this range, whereas the other two groups range from the central Tibetan Himalaya and Trans-Himalaya to western Gansu. This means that the three groups overlap in only the central part of the Trans-Himalaya in southern Tibet. A group including all three subgroups is strongly supported as monophyletic in all concatenated and single-gene analyses (Chart 1, line 82) and by eight unique bases and two unique amino acids (Suppl. material 1: Tables S1, S2). The two species of *Psilonebria* represented in our sample (Nebria cf. superna Andrewes, 1923 (Fig. 3D) and *Nebria* sp XIZ 20 [undescribed]) are supported as a monophyletic group (Fig. 4C) in all of our analyses (Chart 1, line 83), whereas a monophyletic *Asionebria* is contra-indicated in all analyses (Chart 1, line 84). Instead, both *Psilonebria* and the *przewalskii* group species are nested within *Asionebria* in trees from all concatenated analyses except the Mito G analysis (Suppl. material 2: Fig. S4)) and all single-gene analyses except COIBC, COIPJ and CAD2 (Suppl. material 2: Figs S7–S9). Our results suggest that this group represents a single lineage that should be recognized as a single subgenus. Members of this group share the following morphological features: head distinctly wider than average for the genus relative to other body proportions; antennomeres 3 and 4 laterally compressed basally (except in *Nebria przewalskii* Semenov, 1889); metepisterna impunctate; and median lobe of male genitalia without a sagittal aileron but with a sclerotized basal collar. The name *Psilonebria* has priority over *Asionebria*, so that should be the name of this subgenus.

The second clade in the *Eunebria* Subcomplex includes *Eurynebria* (*N. complanata*) and the remaining species of *Eunebria* in our sample (Fig. 4C). *Nebria complanata* (Fig. 2F) once occupied sandy sea beach habitats on the southern Irish Sea and Atlantic coasts of Ireland and the United Kingdom and along Atlantic coastal Europe and northern Africa from Belgium and France to central Morocco. It has been recorded also from the sandy Mediterranean shores of France, Italy, Turkey, Algeria, and Tunisia [the record from Iran (Azadbakhsh and Nozari 2015, Huber 2017) requires confirmation]. Unfortunately, many of the former populations of this attractive species have been extirpated (A. Casale, pers. comm.). The subgenus Eunebria (excluding the *przewalskii* group) is comprised of almost 50 described species-group taxa, collectively ranging from northern Africa and western Europe to Japan and Taiwan, but with endemic clusters of species in largely disjunct areas between these extremes. Three species occur in Europe, with a fourth species (likely related to them, Ledoux and Roux 2005) in Morocco. Five species occur in the area including the Caucasus, Elbruz, and Zagros mountains from Georgia to central Iran. Twenty-five species occur in central Asia, arrayed in a broad semicircular pattern around the Taklimakan Desert and edge of the Tibetan Plateau, from northern Mongolia and Tuva in Russia, southwest to eastern Uzbekistan and Afghanistan, then southeast along the Himalayan Mountains to central Nepal. Another group of five described and at least three undescribed species occupies an area on the southeastern edge of High Asia in northeasternmost India, Yunnan and central to southern Sichuan. Four species occur in far eastern Asia (including two in Japan, one in Taiwan, and one on the Chinese mainland in Fujian Province). Finally, a single, distinctive species, *Nebria tetungi* Shilenkov, 1982, occupies an area from northern Sichuan to easternmost Qinghai Province in China. This last species has been described twice as the type species of a new subgenus (i.e., *Tetungonebria* Shilenkov, 1982 and *Sphodronebria* Sciaky & Pavesi, 1994), but Ledoux and Roux (2005) included it in *Eunebria*. We were not able to include this species in our molecular sample and its relationships to other *Nebria* species remain unclear. This group is supported as monophyletic by all our analyses (Chart 1, line 85) and by three shared unique bases, one unique amino acid and one unique base insertion in 28S (Suppl. material 1: Tables S1–S3).

We had only ten (22%) of the described taxa included in our sample of this subcomplex, so our results represent only a preliminary assessment of relationships within the group. With respect to the relationship between *Eurynebria* and *Eunebria*, several possibilities remain viable. There is some support for a clade including all *Eunebria* as sister to *Eurynebria* (Chart 1, line 86) from the 8GML (Fig. 5C), 8G B and Mito G (Suppl. material 2: Figs S1, S4) concatenated analyses and also from the COIPJ and Topo analyses (Suppl. material 2: Figs S6, S11). Although none of the evidence is compelling, the Nuc G and NPC G concatenated analyses and 28S, COIBC, CAD2, and *wg* analyses all support *Eurynebria* as nested within *Eunebria* and related to one or another of the clades included within the latter (Chart 1, lines 87–89). Each of these clades occupies one or two of the focal areas for *Eunebria* described above. One clade includes the European and central Asian species in our sample. This clade is supported by all our analyses except the 28S analysis (Chart 1, line 90) and by two shared unique bases (Suppl. material 1: Table S1). Results from the CAD2 analyses show *Eurynebria* as sister to this clade (Suppl. material 2: Fig. S9). Within this clade the European and central Asian species each form sister clades (Fig. 4C) supported by all our concatenated analyses and by three or more single-gene analysis (Chart 1, lines 91 and 92). Results from the 28S analysis suggest that *Eurynebria* is most closely related to the European clade, a result that makes sense geographically. Another clade within *Eunebria* is one including the species in our sample from southeastern Asia (centered in Yunnan Province) and far eastern Asia. This east Asian clade is well supported by all our analyses (Chart 1, line 93). Results from the Nuc G and NPC G concatenated analyses and the *wg* single-gene analysis suggest that *Eurynebria* is sister to this clade (Suppl. material 2: Figs S2, S3, S12).

Given the conflicting evidence from our results, we cannot establish unambiguously whether *Eurynebria* is sister to *Eunebria* or nested within it. This is an important distinction for taxonomy because *Eurynebria*, which has included only *N. complanata* since its introduction, has priority over *Eunebria* if the two groups are united. We choose to leave these subgenera as separate pending additional evidence supporting the alternative.

### *Catonebria* Series: *Reductonebria* Complex

This complex corresponds to subgenus Reductonebria as treated by Ledoux and Roux (2005). The group includes 40 described species-group taxa with a combined geographical range that extends from the Altai Mountain system of central Asia eastward to eastern North America, but with gaps inside this range. The group is well supported by all of our concatenated and single-gene analyses (Chart 1, line 95) as well as by four synapotypic amino acids and two unique bases (Suppl. material 1: Tables S1, S2). Within this complex, three distinct lineages are apparent (Fig. 4D). The first includes just two species, *Nebria carbonaria* Eschscholtz, 1829 and *Nebria snowi* Bates, 1883. This group corresponds to Ledoux and Roux’s “*carbonaria* group”. *Nebria carbonaria* occurs on the Kamchatka Peninsula and the northern Kuril Islands (Paramushir and Onekotan Islands) and *N. snowi* is endemic to the Kuril Islands. This clade is so strongly supported in all our analyses (Chart 1, line 96), as well as by two unique bases (Suppl. material 1: Tables S1, S2), that we recognize it as a distinct subgenus, *Insulanebria*. Morphological features that unambiguously distinguish members of this group from those of other members of the *Reductonebria* Complex are few and mainly involve internal features (see the Taxonomy section for discussion of these characters).

The second lineage in the complex is subgenus Reductonebria (Fig. 4D). This group is comprised of 17 species, some of which occupy very large geographical ranges. The combined range of the group is that of the entire complex. This clade is also very strongly supported in all of our analyses (Chart 1, line 99) and by one synapotypic amino acid (Suppl. material 1: Table S2) and one unique base insertion in 28S (Suppl. material 1: Table S3). Within this clade, an initial divergence among the 15 taxa in our sample resulted in vicariant Palearctic and Nearctic sister clades. The Palearctic clade, equivalent to Ledoux and Roux’s “*ochotica* group”, includes at least three species, one geographically widespread, the others more restricted in distribution. *Nebria altaica* Gebler, 1847 is restricted to the region from the southern end of Lake Baikal west through the Eastern and Western Sajan Mountains to the western end of Altai Mountains. *Nebria ochotica* Sahlberg, 1844 ranges from Yakutia in central Siberia east to the Russian Far East, Sakhalin Islands, Kamchatka and the Kuril Islands, Hokkaido Island in Japan, and south along the mainland coastal region to North Korea. *Nebria japonica* Bates, 1883 appears to be restricted to the mountains of Honshu Island, Japan. The only species of this subgenus not represented in our taxon sample were *Nebria angustula* Motschulsky, 1866, which is endemic to the Kamchatka Peninsula, and *Nebria nicolasi* Ledoux & Roux, 2006, which is known only from the Qionglai Mountains of central Sichuan Province, China. Members of these two species each have unique features that put their close relationship to other species in this group in question, but we have no evidence at present to place them anywhere but in the Palearctic group. Results from all our concatenated analyses as well as the COIBC, COIPJ, CAD2, and Topo analyses support the three species of this group on our sample as a clade (Chart 1, line 100).

The Nearctic group of *Reductonebria* is comprised of 12 species (Fig. 4D), including all those assigned to the “*pallipes*”, “*obliqua*”, and “*mannerheimi*” groups by Ledoux and Roux (2005) or the “*appalachia*”, “*pallipes*”, “*mannerheimi*”, and “*obtusa*” groups of Lindroth (1961). Nine of these occur only in the west, all living at relatively low elevations for *Nebria* species, with a combined geographical range extended from the Kenai Peninsula in southcentral Alaska south to southern California, northern Arizona and New Mexico, and east as far as the Black Hills of western South Dakota and northwestern Nebraska. *Nebria mannerheimii* Fischer von Waldheim, 1828, *Nebria eschscholtzii* Ménétriés, 1844, and *Nebria obliqua* LeConte, 1866 occupy large geographical ranges while the six other western species are geographically more restricted. Two species occur only in the east: *Nebria pallipes* Say, 1823 ranges broadly from the Canadian Maritime Provinces south along the Appalachian Mountains to northern Georgia and South Carolina and west to Michigan, Illinois, and Kentucky. *Nebria appalachia* Darlington, 1932 is restricted to the highest elevations of the southern Appalachian Mountains in North Carolina and Tennessee. The last species, *Nebria suturalis* LeConte, 1850, has a glacio-relictual distribution including the tops of the highest peaks in the Green Mountains of Vermont and White Mountains of New Hampshire, streams along the Labrador coast and coasts of Ungava and Hudson Bays in Québec, the north shore of Lake Superior in Ontario, the margins of glaciers in the Rocky Mountains of central Alberta, and high mountain summits in the southern Rocky Mountains of central Colorado. The monophyly of this group is supported by results from all of our concatenated analyses and from the 16S-ND1, COIPJ, and Topo analyses (Chart 1, line 101; Fig. 5D; Suppl. material 2: Figs S1–S4, S8, S11). Within this group, relationships generally are not well resolved by our molecular data. The species pair, *N. mannerheimi* and *Nebria darlingtoni* Kavanaugh, 1979, is supported as monophyletic in all analyses and as sister to the other species in the group in the 8GML, 8G B, Nuc G, and NPC G concatenated analyses and CAD2, PEPCK, Topo, and *wg* analyses. Two other species pairs, *Nebria diversa* LeConte, 1863 with *N. eschscholtzii* and *N. pallipes* with *N. appalachia*, are also supported as monophyletic in all or most analyses, respectively (Fig. 5D; Suppl. material 2: Figs S1–S12). We find no other consistent pattern of relationships within this clade.

The third lineage in the *Reductonebria* Complex (Fig. 4D) is comprised of 21 species and equivalent to the “*sahlbergi*” and “*gregaria*” groups of Ledoux and Roux (2005) or the “*gregaria* group” of Lindroth (1961). This is an endemic western Nearctic lineage with a combined geographical range extending from the outer Aleutian Islands of Alaska south to the central Sierra Nevada of California, northern Arizona and New Mexico, and east to streams draining the eastern slopes of the Rocky Mountains from Alberta to New Mexico. Monophyly of this group is supported by all of our analyses except for the Topo analysis (Chart 1, line 102), and we recognize this group as a distinct subgenus, *Erwinebria*. The morphological features that unambiguously distinguish members of this group from those of other members of the *Reductonebria* Complex mainly involve internal features (see the Taxonomy section for discussion of these characters). As for subgenus Reductonebria in North America, branch lengths in this group are very short, suggesting recent divergences, and resolution of relationships among the included species is generally poor. However, some patterns are still evident. *Nebria lyelli* Van Dyke, 1925 from high elevations in the central Sierra Nevada of California is shown as sister to a clade including all other species in trees from all concatenated analyses and the COIBC, COIPJ, and *wg* analyses (Fig. 4D, Suppl. material 2: Figs S1–S4, S7, S8, S12). *Nebria danmanni* Kavanaugh, 1981 from high elevations on the Olympic Peninsula in Washington is shown as sister to a clade including the remaining group taxa in these same analyses except for COIBC. In general, several subgroups recognized by Kavanaugh (1978) are shown as monophyletic only in some but not all analyses or with changes in their species composition. For example, he included *Nebria edwardsi* Kavanaugh, 1979 (described as a subspecies of *Nebria arkansana* Casey, 1913) in his *arkansana* subgroup along with *N. arkansana*, *Nebria fragilis* Casey, 1924, *Nebria zioni* Van Dyke, 1943 and *Nebria oowah* Kavanaugh,1979 (also described as a subspecies of *N. arkansana*). Results from all concatenated analyses except Nuc G and NPC G and all three mitochondrial single-gene analyses support the monophyly of a clade including the other four species (Chart 1, line 104), but no analysis provides support for including *N. edwardsi* in that clade (Chart 1, line 103). A clade including *Nebria gregaria* Fischer von Waldheim, 1822, *Nebria haida* Kavanaugh, 1984, *Nebria charlottae* Lindroth, 1961, *Nebria louiseae* Kavanaugh, 1984, and *Nebria lituyae* Kavanaugh, 1979 (the *gregaria* subgroup) is supported by our Nuc G, NPC G, 28S, and *wg* analyses (Chart 1, line 105). However, a clade including all those species but excluding *N. lituyae* is supported by 8GML, 8G B, Mito G concatenated, and COIBC and COIPJ single-gene analyses (Chart 1, line 106). In this case, the nuclear and mitochondrial genes are suggesting different patterns of relationship.

Relationships among the three lineages also remain ambiguous. Results from the 8GML, 8G B and Mito G concatenated analyses and the 16S-ND1, PEPCK, and *wg* analyses (Chart 1, line 98) support a clade including *Reductonebria* and *Erwinebria* as sister to *Insulanebria*. This relationship is also supported by two unique bases (Suppl. material 1: Table S1). Evidence supporting a clade including *Insulanebria* and *Reductonebria* as sister to *Erwinebria* is provided by the Nuc G and NPC G concatenated and COIPJ, CAD2, and Topo single-gene analyses (Chart 1, line 97). The ML tree from the COIBC analysis (Suppl. material 2: Fig. S7) shows these three subgenera as an unresolved trichotomy, which probably represents the best summary of all the evidence at this time.

### *Catonebria* Series: *Catonebria* Complex

At present, this complex includes a single subgenus, *Catonebria*, named for the chain-like appearance of alternating elytral intervals seen in many but not all members of this group (Fig. 3F). This visual effect is the result of more or less deeply foveate setiferous pores on all or some of intervals 3, 5, 7, and 9 and the associated distortions of the adjacent striae. Ledoux and Roux’s (2005) concept of subgenus Catonebria corresponds to the entire complex, whereas Lindroth (1961), who did not distinguish subgenera in his treatment, arrayed the Nearctic species in three species groups (his “*metallica*”, “*ingens*”, and “*ovipenni*s” groups). This complex is comprised of 64 described species-group taxa, including some of the largest and most colorful species in the genus. The combined geographical range of the complex extends from the Altai and Dzungarian Alatau mountain systems of southcentral Siberian Russia and eastern Kazakhstan, respectively, eastward across Asia, through the Aleutian Islands and Alaskan mainland to streams draining the eastern flanks of the Rocky Mountains from Alberta to southern New Mexico. The southern limits of this range in Asia include southeastern Kazakhstan, northwestern Xinjiang and Shanxi Province in China, northern Mongolia, and the eastern Asian coast as far south as North Korea. The complex has not been recorded from Japan or Taiwan. In North America, the group ranges south into the Sierra Nevada in California and into isolated mountain ranges scattered across southern Nevada, eastcentral Arizona and southcentral New Mexico. Support for the monophyly of this complex is provided by all the concatenated analyses and by the 28S, COIBC and PEPCK analyses (Chart 1, line 107). Support against this clade comes from our CAD2 and *wg* analyses (Chart 1, line 108, Suppl. material 2: Figs S9 and S12), which suggest that part of the group (see below) is more closely related to the *Reductonebria* Complex than to the remainder of the *Catonebria* Complex. In our judgment, the evidence for this complex as a single monophyletic group outweighs the contradictory evidence.

Within the complex, three distinct lineages are evident (Fig. 4D). The first includes just two western North American species. *Nebria paradisi* Darlington, 1931 occurs at high elevations on the volcanic peaks of the Cascade Range from northern Washington south to Mount Hood in northern Oregon. *Nebria turmaduodecima* Kavanaugh, 1981 occurs only on the highest peaks of the Trinity Alps in northwestern California. Members of both species live at the edges of glaciers, permanent snowfields, and meltwater streams, so the outlook for their continued survival worsens as these habitats shrink and disappear and global and regional temperatures rise. Ledoux and Roux (2005) included both of these species in their subgenus Nakanebria and Lindroth (1961) included *N. paradisi* in his “*ovipennis* group”. This species pair is strongly supported as a clade by all our analyses (Chart 1, line 107) and by one unique amino acid and four unique bases (Suppl. material 2: Tables S1, S2). However, results from our CAD2 and *wg* analyses suggested that this was more closely related to the *Reductonebria* Complex than to the other *Catonebria* Complex members. Because of its distinctiveness from *Catonebria*, we recognize this group as a distinct subgenus, *Nivalonebria*. Both internal and external morphological features unambiguously distinguish members of this group from those of other members of the *Catonebria* Complex (see the Taxonomy section for discussion of these characters).

The second lineage in the complex is comprised of 20 described species-group taxa, which Ledoux and Roux (2005) included in their “*mellyi*” and “*kincaidi*” groups. Lindroth (1961) was aware of only two Nearctic species in this group and assigned them both to his “*ovipennis* group” along with *N. paradisi*. The geographical distribution of this group is one of the most remarkable among all Nebriitae. A group of six species and an additional eight subspecies occupy a relatively small area in central Asia including the Western Sayan, Altai, and Kuznetsky Alatau mountain systems of southcentral Siberia and northeastern Kazakhstan. A second group of six species occurs in western North America, with a combined range extending from southeastern Alaska south to the southern Sierra Nevada in California and east across the Columbia Plateau to the Salmon River Mountain system of central Idaho and the Bitterroot Mountains of western Montana. This disjunction between the respective areas is more than 7000 km, and extensive collecting in many of the intervening areas has failed to discover additional populations or species. All members of this group are flightless and live in restricted cool or cold habitats around snowfields and glaciers and along meltwater streams at high elevations or along cool, shaded, forested streams at middle elevations. The monophyly of this group is well supported by all of our analyses (Chart 1, line 113) and by one amino acid unique among nebriites, a unique base and a unique base insertion in 28S (Suppl. material 2: Tables S1–S3). In addition, the Palearctic and Nearctic groups of species are well supported as separate clades in all of our concatenated analyses and all or most, respectively, of our single-gene analyses (Chart 1, lines 114 and 115; Fig. 5D; Suppl. material 2: Figs S1, S12)). Consequently, we recognize these groups as distinct subgenera. The Palearctic clade, subgenus Palaptenonebria, is also supported by one amino acid unique among *Nebria* and one unique base and the Nearctic clade, subgenus Neaptenonebria, by one unique amino acid and one unique base (Suppl. material 1: Tables S1, S2) (see the Taxonomy section for discussion of morphological features that characterize these two clades). Branch lengths within the *Palaptenonebria* clade are generally shorter than in the *Neaptenonebria*, clade, which suggests more recent divergence among the former than among the latter.

The third lineage in the Catonebria Complex is subgenus Catonebria itself. In our concept of the subgenus, it is comprised of 42 species-group taxa with a combined geographical range equal to that of the entire complex. Ten species and one additional subspecies occur in the Palearctic Region, while the remaining 31 species are endemic to western North America. Both faunas include both widespread and geographically restricted species, as well as taxa with full-sized hindwings and others with reduced hindwings incapable of supporting flight. Some of the beetles in this subgenus possess vivid and stunning metallic reflection and coloration, as well as the most well-developed catenation of the elytral intervals (Fig. 3F). This group includes species arrayed in Ledoux and Roux’s (2005) “*catenulata*”, “*metallica*”, “*trifaria*”, “*scaphelytra*”, and “*suensoni*” groups and Lindroth’s (1961) “*metallica*” and “*ingens*” groups. Results from all of our concatenated and analyses except the PEPCK analysis support the monophyly of this group (Chart 1, line 116). Branch lengths among the terminal species are very short in ML trees from all the nuclear gene analyses, slightly longer in trees from the mitochondrial gene analyses, so resolution of relationships among the terminal branches is unsatisfactory. Nonetheless, a few relationships within the subgenus are clear. There is no vicariance relationship evident between all the Palearctic species in our sample and all the Nearctic species because the former are nested within the Nearctic group. The Palearctic species are seen as a single clade only in ML trees from the Nuc G, NPC G, and 16S-ND1ML analyses (Suppl. material 2: Figs S2, S3, S6), but otherwise are arrayed in different combinations among the Nearctic taxa (Suppl. material 2: Figs S5, S7, S9–S12). Three Palearctic species are missing from our sample. One of these, *Nebria baicalopacifica* Dudko & Shilenkov, 2006 is closely related to other species in Dudko’s “*catenulata*” species group and its inclusion is unlikely to have altered our results substantially. However, the other two species, *Nebria scaphelytra* Kavanaugh & Shilenkov, 1996 from North Korea and *Nebria suensoni* Shilenkov & Dostal, 1983 from northeastern Shanxi Province, China are very distinct (both were recognized as monotypic species groups by Ledoux and Roux 2005), so adding them to our the sample could have produced different results. Within the Nearctic fauna, several groups of species are seen repeatedly in ML trees from different analyses and are identified in Fig. 4D. The *meanyi* group of four species is shown in every ML tree, both the *ingens* group of two species and the *trifaria* group of eleven species in all but one ML tree (PEPCK and COIPJ, respectively), the *vandykei* group of three species and *gebleri* group of five species in all but two ML trees, and the *piperi* group of four species in all but one (Mito G) of the concatenated gene trees and three of the single-gene trees (28S, PEPCK, and Topo). Each of these groups is strongly supported (support values ≥ 99) as monophyletic by four or more of the five concatenated gene analyses and by at least three single-gene analyses with support values ≥ 50 and most much higher (Fig. 5D; Suppl. material 2: Figs S1–S12). Strongest support values from single-gene analyses are seen mainly with the mitochondrial ML trees. Unfortunately, the pattern of relationships among these groups is highly inconsistent between ML trees from different single-gene analyses.

A clade including *Palaptenonebria* and *Neaptenonebria* with *Catonebria* as sister to *Nivalonebria* (Fig. 4D) is supported by the 8GML, 8G B, Nuc G, and NPC G concatenated analyses (but only at support values ranging from 54–71) and by the *wg* analysis (MLB value = 55) (Chart 1, line 110). Contradictory support for a clade including *Nivalonebria* with *Palaptenonebria* and *Neaptenonebria* as sister to *Catonebria* is provided by the Mito G concatenated analysis and the COIBC analysis, but again with only modest support levels (Chart 1, line 111). A clade including *Nivalonebria* with *Catonebria* as sister to *Palaptenonebria* and *Neaptenonebria* is also supported, but only by the 28S analysis (Chart 1, line 112). Although we would prefer to have better support for the pattern of relationships, we regard the stronger evidence as supporting sister status for *Palaptenonebria* + *Neaptenonebria* and *Catonebria*, with that trio as sister to *Nivalonebria*.

### Uncertain relationships among closely related species

Although many of the deeper nodes in the phylogeny were reconstructed with confidence, we found poor resolution among recently diverged (terminal) taxa. Within several of the species groups, we could not confidently reconstruct phylogenetic relationships based upon the data at hand. This was particularly true for groups within subgenera of the *Catonebria* Series. Given the low genetic divergences observed, as indicated by the short branches within these groups in the phylogenetic trees, this is not surprising, for two reasons. Only ca. 5100–5600 bases were analyzed for most species, which is likely insufficient because of the low per-site differences observed; a larger fraction of the genome is likely needed to ensure that the short internal branches are represented in the data by derived bases marking the presence of those branches. In addition, the short internal branches suggest short intervals between speciation events, and thus are likely regions of the tree with at least some incomplete lineage sorting (Pamilo and Nei 1988); one therefore expects that there will be conflict among gene trees of different linkage groups, as well as with the actual species tree. With at most six linkage groups studied (the mitochondrial genome as well as five nuclear genes), and with the short internal branches, we do not expect to be able to see a clear signal of the shape of the species tree for those regions of rapid divergence.

### Summary of phylogenetic relationships

A summary of phylogenetic relationships among the tribes, genera, and subgenera of supertribe Nebriitae based on our analyses is presented in Fig. 6. All clades shown in that tree are supported by MLB and BPP values greater than 95 except for the clade including subgenera *Sardoleistus* + *Pogonophorus* + *Evanoleistus* + *Leistus* s. str. of genus *Leistus* (MLB = 79, BPP = 100) and the clade we have called the *Nebria* Series in genus *Nebria* (MLB = 79, BPP = 100). The tribes of nebriites are each strongly supported as monophyletic, but relationships among them are not yet well resolved, so we show them as an unresolved quadritomy. Within genus *Leistus*, *Nebrileistus* is well supported as sister to the remaining subgenera and *Evanoleistus* and *Leistus* s. str. form a well-supported clade, but relationships between this clade and *Sardoleistus* and *Pogonophorus* are poorly resolved and are shown as an unresolved trichotomy. In genus *Nebria*, the *Oreonebria* Series is well supported as sister to a clade including the remaining three series of the genus. However, relationships among the *Nebriola*, *Nebria*, and *Catonebria* Series remain uncertain, so they are shown as an unresolved trichotomy. The same is true for the three subgenera recognized in the *Reductonebria* Complex and three main lineages in the *Catonebria* Complex, although the *Catonebria* Series is very strongly supported as a clade. The above exceptions notwithstanding, the tree is well resolved and strongly supported. A revised classification of the Nebriitae based on the results of this study is provided in Table 4.

### Concordance of geography with phylogeny

A sound hypothesis of phylogenetic relationships within any group of taxa is an essential prerequisite for interpreting the evolutionary and biogeographic significance of their observed geographical relationships. Facilitating a better understanding of the biogeographic and evolutionary histories of the Nebriitae, and particularly of the nebriines, was a prime motivation for this project. Although our sampling of several of the larger subgenera of *Leistus* and *Nebria* was inadequate to encompass their full diversity, a few overall conclusions can be drawn from our results. First and most importantly, we recognize a general consistency between the phylogenetic structure suggested by our analyses, particularly within the Nebriini, and the geographic cohesion of the clades recognized. This provides us with an added measure of confidence in our phylogenetic results. Although additional molecular sampling will be required to establish the geographical relationships of species groups in several subgenera, particularly within *Leistus* s. str., *Evanoleistus*, *Eonebria*, *Boreonebria*, *Nebria* s. str., *Epinebriola*, and *Eunebria*, a few clear and concordant vicariance patterns are already evident among the better-represented groups. For example, we recognize vicariance relationships between eastern Asia and western North America in one generic pair (*Opisthius* vs. *Paropisthius*), two subgeneric pairs (*Nippononebria* s. str. vs. *Vancouveria* and *Neaptenonebria* vs. *Palaptenonebria*) and at least four species group pairs (*Leistus niger* and related species vs. Nearctic species [formerly assigned to *Neoleistus*] in *Leistus* s. str.; *Nebria gyllenhali* and related species vs. *N. castanipes* and related species in *Boreonebria*; Palearctic vs. Nearctic *Reductonebria* species; and the Palearctic species vs. the *N. metallica* + *N. labontei* + *N. trifaria* group clade of Nearctic *Catonebria*).

Our results suggest that dispersal resulting in amphi-Pacific disjunct distributional patterns occurred at different times in the evolutionary history of Nebriitae. Deep splits such as in *Opisthius/Paropisthius* and *Nippononebria* s. str./*Vancouveria* may have resulted from dispersal events to western North America simultaneously with the Late Cretaceous-Paleogene formation of the Cordilleran Mountain system as it was hypothesized for metriine beetles (Wrase and Schmidt 2006). This chronological scenario becomes realistic due to fossil evidence of a stem group representative of *Nippononebria* in Eocene Baltic amber (*Archaeonebria* Kavanaugh & Schmidt in Schmidt et al. 2019). *Archaeonebria* was hypothesized as sister group of *Nippononebria* + *Vancouveria*. During the Eocene, the distributional pattern of *Nippononebria* must have been trans-Palearctic or even Holarctic. In the course of the further development of the Palearctic fauna, the lineage from the western part of the pre-Palearctic went extinct. Younger splits within amphi-Pacific distributed species groups of *Leistus* and *Nebria* may have resulted from Neogene exchange of cold temperate and boreal faunal elements across Beringia, which occurred until the Pliocene (Sanmartin et al. 2001; Liebherr and Schmidt 2004).

Similarly, vicariance relationships are evident between Central Asia and the Asian Far East in one subgeneric pair (*Archastes* vs. *Orientonebria*) and at least two potential species group pairs (most *Eonebria* species vs. *N. komarovi* and related species and *N. yunnana* and related species vs. the species pair, *N. lewisi* + *N. uenoi*, in *Eunebria*). Vicariance between subgenus Oreonebria in the Alps and its sister group, *Archastes* + *Orientonebria*, in central and far eastern Asia is a pattern seen in some other carabid groups, such as within the broscine genus *Broscosoma* Rosenhauer, 1846 and the scaritine genus *Reicheiodes* Ganglbauer, 1891. As one last example, we cite vicariance relationships evident between eastern North America and the Rocky Mountain region of western North America. This pattern is shown by two species group pairs (the *N. lacustris* + *N. bellorum* clade vs. the *N. hudsonica* + *N. gouleti* clade of *Boreonebria* and the *N. pallipes* + *N. appalachia* clade vs. *N. georgei* and related species of *Reductonebria*). These and other vicariance patterns are likely to be clarified and/or replicated with additional molecular sampling; and because some involve sister taxa at different hierarchic levels (that is, between generic, subgeneric or species groups pairs), they likely reflect different divergence times. Future efforts will be directed at further examination of the spatial and temporal relationships implied by the phylogeny. Time calibration of our tree should help to correlate these and other biogeographically interesting vicariance relationships more precisely with events in Earth’s history, but that task was not part of the present study.

## Taxonomy

### A revised classification of Nebriitae

Based on results from this study, we present a revised classification of the supertribe Nebriitae to the genus-group level as summarized in Table 4. In relation to the classification proposed by Ledoux and Roux (2005), this classification includes three genus-group taxa (*Nippononebria*, *Vancouveria* and *Archastes*) with revised status, seven (*Nebriorites*, *Marggia*, *Pseudonebriola*, *Patrobonebria*, *Paranebria*, *Barbonebriola*, and *Asionebria*) as new synonymies, and six (*Parepinebriola*, *Insulanebria*, *Erwinebria*, *Nivalonebria*, *Neaptenonebria*, and *Palaptenonebria*) as new subgenera (which are described below). As noted above in the Discussion section for the Nebria Subcomplex, subgenus Nebria s. str. was inadequately sampled by our study to determine whether it is paraphyletic or polyphyletic as presently comprised (that is, with *Alpaeonebria* and *Spelaeonebria* retained as separate subgenera). Additional taxon sampling within this large complex is needed to better resolve relationships among the subgenera, including others (*Alpaeus*, for example) that may need to be resurrected or described. The same is true for subgenus Eunebria and its relationship to *Eurynebria*, as noted in the Discussion section for the *Eunebria* Subcomplex.

The most significant question that we had to address in establishing the classification presented here was whether to retain *Nebria* as a single large genus, as did Ledoux and Roux (2005), or to break it up into several smaller genera equivalent to what we treat here informally as “series”, “complexes” or “subcomplexes” of subgenera. Whereas our analyses of DNA sequence data show each of these complexes and subcomplexes as well supported clades, there is little concordant morphological support for them at present, although the required thorough morphological study is yet to be done. Consequently, the fragmentation of *Nebria* into four genera (equal to the four series), six genera (equal to the complexes) or ten genera (equal to the complexes and their individual subcomplexes) would result in the proliferation of genera only poorly or not at all circumscribed morphologically. It could also require significant nomenclatural modification of specific names that might then come out of synonymy. *Nebria*, in the broad sense as maintained here, is a well-known, morphologically cohesive entity. Hence, we view the classification presented here as the best compromise at this time to reflect the available evidence about phylogenetic relationships within the genus as well as to minimize nomenclature instability.

Explanation of certain morphological characters used in diagnoses and discussions. We use the term standardized body length (SBL) as a measure of body size. It is the sum of three separate measures: head length, measured along the midline from the apical margin of the clypeus to a point level with the posterior margin of the eyes; pronotal length, measured from apical to basal margin along the midline; and elytral length, measured along the midline from the apex of the scutellum to a point level with the apex of the longer elytron. This measure of total length eliminates problems with misalignment or telescoping of the three body tagmata, but it also underestimates overall body length by ca. 15%. What we call the “mid-shaft” of the median lobe of the aedeagus of males is the middle section of this tubular structure basal to the apical orifice. We use the term “gonocoxa” for the basal part of the female ovipositor, which Deuve (1993) called the “gonosubcoxite”. We describe the bursa copulatrix of females as divided into a “vestibular chamber”, which is the expanded basal (posterior) part of the bursa immediately internal to the gonopore, and a “spermathecal chamber”, which is the part of the bursa to which the spermathecal duct is attached. The two “chambers” may be quite distinct or merge, one with the other, without a clear distinction. These and all features mentioned in the following sections are more fully discussed in Kavanaugh (1978).

#### Subgenus Parepinebriola

Taxon classificationAnimaliaColeopteraCarabidae

Kavanaugh, subgen. nov.

A3FC909F-CAF0-5161-9EA8-722B9782BB36

http://zoobank.org/3DEF2B4B-DD00-4EBE-B0FD-BCFB6611903B

Epinebriola Daniel & Daniel, 1904 (in part); Huber and Schmidt 2017 (in part)

##### Type species.

*Nebria delicata* Huber & Schmidt, 2017:59, by present designation.

##### Diagnosis.

Body size small or very small, SBL = 8.0 to 9.9 mm. Head moderately wide, slightly constricted behind eyes; vertex with a single central pale spot or a larger pale area and with a single pair of supraorbital setae present. Eyes slightly to moderately reduced in size, moderately to markedly convex. Antennal scape with one or two subapicodorsal setae; antennomeres 3 and 4 not laterally compressed, without extra setae. Labrum with three or four pairs of apical setae. Maxillary stipes typical for genus, with setae inserted flush on smooth surface. Penultimate labial palpomere with three setae. Pronotum with one midlateral seta (a second lateral seta present unilaterally in a few specimens) and one basolateral seta present on each side. Elytral intervals smooth, without macrosculpture, interval 3 with one to seven setiferous pores, intervals 5 and 7 without setiferous pores, intervals 3, 5, and 7 without catenations. Hindwings reduced to short, membranous lobes with only faint vestiges of venation. Metepisterna smooth to sparsely and faintly punctate. Protarsomeres 1–3 expanded in males; mesotarsomeres 2–4 longer than their apical width; tarsi dorsally glabrous or with only a few fine setae. Abdominal sternites IV to VI without paralateral setae. Median lobe of male aedeagus sclerotized dorsally at least to midlength on shaft; basal bulb only slightly expanded relative to shaft diameter and broadly open basally and with lateral and dorsal basal collar, without or with only slightly developed laterobasal lobes; sagittal aileron present at base as a small midline fin attached to collar; mid-shaft slightly tapered apically and with lateral right face unmodified; apical orifice mid-dorsal. Right paramere slender and moderately long. Female valvifers with vestiture; gonopods VIII fused to dorsomedial bases of gonocoxae; gonocoxae with ventral diagonal row of setiform setae and mediodorsal row of setae present. Bursa copulatrix without or (in one species) with dorsal paramedial sclerotized plates in vestibular chamber; spermathecal chamber broadly wedge-shaped, triangular in dorsal aspect, without a posterodorsal extension from its longitudinal axis in lateral aspect and without dorsal, anterodorsal or ventral sclerites; spermathecal duct of medium length, slender and uniform in diameter throughout or nearly so or (in one species) distinctly and abruptly thicker proximally, inserted basodorsally on spermathecal chamber; spermathecal reservoir moderately long.

##### Etymology.

The subgeneric epithet is a noun of feminine gender and a combination of the Greek word, *para*, meaning near, and the genus-group name, *Epinebriola*, in reference to the marked similarity of members of this group to those of subgenus Epinebriola.

##### Remarks.

As noted above in the Discussion section, the described members of this group were previously assigned to subgenus Epinebriola. We had no reason to doubt this assignment until after we had reviewed the results of our analyses. Two of the three undescribed species had also been tentatively identified as *Epinebriola* spp. The third shared more external features with *Eonebria* species than with *Epinebriola* species and had been identified initially as belonging to the former group. A subsequent, more detailed re-examination of both external and internal morphological features revealed a few differences that appear to distinguish members of this group from those of *Epinebriola*. In males: the median lobe of the male genitalia has a basal bulb without or with only slightly developed laterobasal lobes, whereas these lobes are moderately- to well-developed in *Epinebriola*; a sagittal aileron is present dorsobasally as a small midline fin attached to a basal collar, but the aileron is absent or present only as a flat, lightly sclerotized collar in *Epinebriola*; and the apical orifice is mid-dorsal, but moderately to markedly deflected right in *Epinebriola*. In females: the spermathecal chamber of the bursa copulatrix is simple, without a posterodorsal extension from the longitudinal axis in lateral view, while it has a slight posterodorsal extension in *Epinebriola*; the spermathecal duct is moderately long and slender (except distinctly thickened proximally only in one species, but short and moderately thick throughout in *Epinebriola*. We note that all *Parepinebriola* members in our sample have a small to large central pale area on the vertex of the head, whereas all the *Epinebriola* members in our sample lack such a pale area. However, we do not know if this distinction holds for the remaining *Epinebriola* not in our sample. *Parepinebriola* members also appear to differ morphologically with those of *Nakanebria* and *Eonebria*. In members of both of these groups, the head is not at all constricted behind the eyes, there is no trace of a sagittal aileron at the base of the male aedeagal median lobe, the basal bulb is quadrate with an expanded basal sleeve, the apical orifice is markedly deflected right, the female spermathecal chamber has a posterodorsal extension from the longitudinal axis in lateral view, the spermathecal duct is inserted mediodorsally on the spermathecal chamber and the spermathecal duct is slightly to extremely long.

Known distribution and diversity. The known geographical range of this clade extends from the central Trans-Himalaya (Gangdise Shan) of southern Tibet eastward to the Gaoligong Shan of northwestern Yunnan Province, China. Presently, it includes five species. Two of these (*N. delicata* and *N. retingensis*) have been described and previously included in *Epinebriola* (Huber & Schmidt, 2017), and the remaining three species are new and will be described in a separate paper.

#### Subgenus Insulanebria

Taxon classificationAnimaliaColeopteraCarabidae

Kavanaugh, subgen. nov.

E9B783A0-DB20-5E80-BD3B-2DAA17E02A8C

http://zoobank.org/B7EC25A1-0006-4636-AD76-BE2DE9F282F1

Reductonebria Shilenkov, 1975 (in part); = “carbonaria” group sensu Ledoux and Roux 2005

##### Type species.

*Nebria carbonaria* Eschscholtz, 1829:24, by present designation.

##### Diagnosis.

Body size small, SBL = 8.2 to 9.5 mm (1–2). Head width medium to slightly broadened, not constricted behind eyes; vertex with a pair of paramedial pale spots and a single pair of supraorbital setae (13). Eyes of moderate size, moderately to markedly convex. Antennal scape with one subapicodorsal seta; antennomeres 3 and 4 not laterally compressed, without extra setae. Labrum with three pairs of apical setae. Maxillary stipes typical for genus, with setae inserted flush on smooth surface. Penultimate labial palpomere with three setae. Pronotum with midlateral setae absent, a single pair of basolateral setae present. Elytral intervals smooth, without macrosculpture, interval 3 with five to eight or more setiferous pores, interval 5 without setae (except one seta present in a few specimens) and interval 7 with zero or two or more setiferous pores, intervals 3, 5 (if setose), and 7 (if setose) moderately catenate. Hindwings reduced to short membranous lobes with only vestigial venation evident. Metepisterna smooth, impunctate. Protarsomeres 1–3 expanded in males; mesotarsomeres 2–4 longer than their apical width; tarsi dorsally glabrous. Abdominal sternites IV–VI with two to four pairs of posterior paramedial setae, without paralateral setae. Median lobe of male aedeagus sclerotized dorsally at least to midlength on shaft, only faintly deflected right in dorsal aspect; basal bulb expanded, triangular or quadrate, broadly open basally and closed dorsally, without a sagittal aileron present at base or with only a lightly sclerotized collar; mid-shaft parallel-sided in lateral aspect, circular or only slightly compressed in cross-section, with right lateral face unmodified; apical orifice slightly to moderately deflected right. Right paramere slender and moderately long. Female valvifers without vestiture; gonopods VIII fused to dorsomedial bases of gonocoxae; gonocoxae with ventral diagonal row of setiform setae and mediodorsal row of setae present. Bursa copulatrix without dorsal sclerites in vestibular chamber and with longitudinal axis slightly arched basodorsally in lateral aspect; spermathecal chamber broadly cordate in dorsal aspect, and without dorsal or anterodorsal sclerites, insemination duct sclerotized as a long, narrow plate ventrally in the chamber; spermathecal duct slightly long, uniform in diameter throughout or nearly so, inserted sub-basodorsally on spermathecal chamber; spermathecal reservoir of medium length.

##### Etymology.

The subgeneric epithet is a noun of feminine gender and a combination of the Latin word, *insula*, meaning island, and the genus name, *Nebria*, in reference to the occurrence of both known species of this clade in Kuril Island Archipelago of far eastern Asia.

##### Remarks.

Members of this group are easily identified as members of the *Reductonebria* Complex using the Ledoux and Roux’s (2005) key to subgenera. They can be distinguished from members of the other two subgenera in this complex, *Reductonebria* and *Erwinebria* by several external and internal features. Most members of both *Insulanebria* species have one or more setiferous pores on elytral interval 7 as well as interval 3, whereas among members of the other subgenera, only a few individuals of Nebria (Reductonebria) diversa LeConte, 1863 have any setiferous pores on interval 7. In addition, the intervals bearing setae in *Insulanebria* members are moderately catenate while they are not or only faintly catenate in members of the other two subgenera except again for some specimens of *N. diversa*. The median lobe of male genitalia is only faintly deflected right in dorsal aspect in *Insulanebria* members, more distinctly deflected right in members of the other groups. Females of *Insulanebria* species have valvifers without vestiture, the longitudinal axis of the bursa only slightly arched basodorsally in lateral aspect, the spermathecal chamber without dorsal or anterodorsal sclerites but with the insemination duct sclerotized as a long, narrow plate ventrally in the chamber, and the spermathecal duct inserted sub-basodorsally on the spermathecal chamber. By contrast, females of the other two subgenera, have valvifers with vestiture, the longitudinal axis of the bursa moderately to markedly sigmoid dorsally in lateral aspect, the spermathecal chamber with either dorsal or anterodorsal sclerites but with the insemination duct not sclerotized in the chamber, and the spermathecal duct inserted basodorsally on the spermathecal chamber.

##### Known distribution and diversity.

The geographical range of this clade is restricted to the Kamchatka Peninsula and Kuril Islands Archipelago. It includes only two species, *N. carbonaria* and *N. snowi*, both of which have previously been included in subgenus Reductonebria.

#### Subgenus Erwinebria

Taxon classificationAnimaliaColeopteraCarabidae

Kavanaugh, subgen. nov.

6226343F-CCF3-514D-B574-B8B0341021C8

http://zoobank.org/12F2832A-C3A8-44B3-9909-32482CEBFE6B

= “gregaria group” sensu Lindroth 1961
Reductonebria Shilenkov, 1975 (in part); = “*sahlbergi*” + “gregaria” groups sensu Ledoux and Roux 2005

##### Type species.

*Nebria sahlbergii* Fischer von Waldheim, 1828:254, by present designation.

##### Diagnosis.

Body size small to medium, SBL = 7.1 to 11.7 mm. Head width medium to slightly broadened, not constricted behind eyes; vertex with a pair of paramedial pale spots and a single pair of supraorbital setae. Eyes slightly reduced to moderate in size, moderately to markedly convex. Antennal scape with one (two in very few specimens) subapicodorsal seta; antennomeres 3 and 4 not laterally compressed, without extra setae. Labrum with three pairs of apical setae. Maxillary stipes typical for genus, with setae inserted flush on smooth surface. Penultimate labial palpomere with three setae. Pronotum with midlateral setae absent, a single pair of basolateral setae present. Elytral intervals smooth, without macrosculpture, interval 3 with three to eight or more setiferous pores, intervals 5 and 7 without setiferous pores, interval 3 not or only faintly catenate, intervals 5 and 7 not catenate. Hindwings full-sized or slightly to markedly reduced in size. Metepisterna smooth or faintly punctulate. Protarsomeres 1–3 expanded in males; mesotarsomeres 2–4 longer than their apical width; tarsi dorsally glabrous or with a few fine setae. Abdominal sternites IV–VI with two to four pairs of posterior paramedial setae, without paralateral setae. Median lobe of male aedeagus sclerotized dorsally at least to midlength on shaft, moderately to markedly deflected right in dorsal aspect; basal bulb expanded, quadrate, broadly open basally and closed dorsally, without a sagittal aileron present at base or with only a lightly sclerotized collar; mid-shaft parallel-sided or slightly to moderately narrowed basally in lateral aspect, slightly compressed in cross-section, with right lateral face unmodified; apical orifice markedly to extremely deflected right. Right paramere slender and moderately long. Female valvifers with vestiture (a few specimens of some species without vestiture); gonopods VIII fused to dorsomedial bases of gonocoxae; gonocoxae with ventral diagonal row of setiform setae and mediodorsal row of setae present. Bursa copulatrix without dorsal sclerites in vestibular chamber and with longitudinal axis of the bursa moderately to markedly sigmoid dorsally in lateral aspect; spermathecal chamber broadly cordate in dorsal aspect, without (in *Nebria lyelli* Van Dyke, 1925 and *Nebria quileute* Kavanaugh, 1979) or with a small to large midline sclerotized dorsal plate, insemination duct not sclerotized; spermathecal duct medium-length to slightly long, uniform in diameter throughout or nearly so, inserted basodorsally on spermathecal chamber; spermathecal reservoir of medium length.

##### Etymology.

The subgeneric epithet is a noun of feminine gender and a combination of the surname of Terry L. Erwin, in whose honor we name this subgenus, and the genus name, *Nebria*.

##### Remarks.

Members of this group are easily identified as members of the *Reductonebria* Complex using the Ledoux and Roux’s (2005) key to subgenera. Features that distinguish them from members of subgenus Insulanebria have been discussed above for that taxon. We have not yet identified any external morphological features that consistently distinguish all members of all species of *Reductonebria* from all *Erwinebria* members, but there are several distinguishing internal features evident in both sexes. The mid-shaft is parallel-sided or slightly to moderately narrowed basally and the apical orifice is markedly to extremely deflected right in *Erwinebria* males. In *Reductonebria* males, the mid-shaft is markedly narrowed apically and the apical orifice is only moderately deflected right. As in most other *Nebria* examined, the gonopods VIII in *Erwinebria* females are fused dorsomedially with the bases of gonocoxae. In *Reductonebria* females, these gonopods are distinctly separate from, although in the same position in relation to, the gonocoxae. The spermathecal chamber is broadly cordate in dorsal aspect in *Erwinebria* females, but somewhat broadly ovoid in *Reductonebria* females. Although most members of both of the subgenera have sclerotized plates in the dorsal or dorsoapical wall of the spermathecal chamber of bursa copulatrix, they are of a different form in the two groups. In *Erwinebria* females (except in *N. lyelli* and *N. quileute*, which have no dorsal sclerites), the dorsal sclerite is variously formed as a small to large domed, horseshoe- or saddle-shaped plate associated with the point insertion of the spermathecal duct on the chamber. In *Reductonebria* females, the sclerotized area is predominately an apicodorsal cap on the spermathecal chamber. This cap sclerite may be relatively small, round, and smooth, as in *N. ochotica*, *N. mannerheimii* and *N. darlingtoni* females, or larger, crenulated rather than smooth, and/or expanded laterally or basally to or beyond the insertion point of the spermathecal duct.

##### Known distribution and diversity.

The geographical range of this clade covers much of western North America, from the outer Aleutian Islands in the northwest, south to eastcentral California, northern Arizona, and New Mexico and east to the eastern edge of the Rocky Mountain system from Yukon Territory in Canada south to northcentral New Mexico.

Twenty-one species in this clade have been described.

#### Subgenus Nivalonebria

Taxon classificationAnimaliaColeopteraCarabidae

Kavanaugh, subgen. nov.

E1AC8532-7E40-5F19-924E-8D07745F9C82

http://zoobank.org/BDE4FBFA-633D-4D30-A57A-10D8A9B294AD

= “ovipennis group” (in part) sensu Lindroth 1961
Nakanebria Ledoux & Roux, 2005 (in part)

##### Type species.

*Nebria paradisi* Darlington, 1931:24, by present designation.

##### Diagnosis.

Body size small to medium, SBL = 8.7 to 11.3 mm. Head markedly broadened, not constricted behind eyes; vertex with a single medial pale area and a single pair of supraorbital setae. Eyes moderate in size, moderately convex. Antennal scape with one subapicodorsal seta; antennomeres 3 and 4 not laterally compressed, without extra setae. Labrum with three pairs of apical setae. Maxillary stipes typical for genus, with setae inserted flush on smooth surface. Penultimate labial palpomere with three setae. Pronotum with one midlateral and one basolateral seta present on each side. Elytral intervals smooth, without macrosculpture, interval 3 with one to seven setiferous pores, interval 5 with zero to two and interval 7 with zero to two or more setiferous punctures, intervals with setae only faintly catenate; elytral medial margins diverge slightly and arcuate from the midline apically. Hindwings reduced to short, slender strap-like vestiges. Metepisterna smooth, impunctate. Protarsomeres 1–3 expanded in males; mesotarsomeres 2–4 longer than their apical width; tarsi dorsally glabrous. Abdominal sternites IV–VI with one to four pairs of posterior paramedial setae, without paralateral setae. Median lobe of male aedeagus sclerotized dorsally at least to midlength on shaft, symmetrical in dorsal aspect; basal bulb expanded, quadrate, broadly open basally and closed dorsally, without a sagittal aileron present at base or with only a lightly sclerotized collar; mid-shaft parallel-sided in lateral aspect, slightly compressed in cross-section, with right lateral face unmodified; apical orifice markedly deflected right. Right paramere slender and very long. Female valvifers without vestiture; gonopods VIII fused to dorsomedial bases of gonocoxae; gonocoxae with ventral diagonal row of setiform setae and mediodorsal row of setae present. Bursa copulatrix with a pair of large dorsal paramedial sclerotized plates in vestibular chamber, with its longitudinal axis moderately sigmoid dorsally in lateral aspect; spermathecal chamber broadly cordate in dorsal aspect, without dorsal sclerites (in some *N. paradisi*) or with a small midline plate (other *N. paradisi*) or large and broad dorsal plate (in *N. turmaduodecima*); spermathecal duct very long and of uniform diameter throughout or nearly so, inserted basodorsally on spermathecal chamber; spermathecal reservoir of medium length.

##### Etymology.

The subgeneric epithet is a noun of feminine gender and a combination of the Latin word, *nivalis*, meaning snow, and the genus name, *Nebria*, in reference to the occurrence of all known members of this clade in the vicinity of montane snowfields and glaciers.

##### Remarks.

Members of this subgenus can be distinguished from those of subgenus Nakanebria, to which they were assigned by Ledoux and Roux (2005), by several external and internal morphological features. Externally, the head is markedly broad, the medial margins of the elytra are slightly and arcuately divergent apically, and the ventroapical margin of metatarsomere 4 is markedly lobate laterally in members of *Nivalonebria* species, whereas the head is of moderate width, the medial margins of the elytra are sinuately divergent apically, and the ventroapical margin of metatarsomere 4 is extremely lobate laterally in *Nakanebria* members. Internally, the apical orifice of the median lobe of male genitalia is markedly deflected right in *Nivalonebria* members, only slightly so in *Nakanebria* members. Females of *Nivalonebria* species have valvifers without vestiture, a bursa copulatrix with a pair of large dorsal paramedial sclerotized plates in the vestibular chamber and with its longitudinal axis moderately sigmoid dorsally in lateral aspect and a spermathecal duct markedly long and inserted basodorsally on the chamber. In contrast, *Nakanebria* females have valvifers with vestiture, a bursa copulatrix without sclerotized plates in the vestibular chamber and with its longitudinal axis only slightly deflected apicodorsally, and a spermathecal duct slightly to markedly short and inserted medioventrally on the chamber. *Nivalonebria* members are distinguished from those of the other subgenera in the *Catonebria* complex externally in having a single medial pale area on the vertex (as opposed to a distinct pair of paramedial pale spots as seen in the other subgenera) and the medial margins of the elytra slightly and arcuately divergent apically. In members of *Neaptenonebria*, *Palaptenonebria* and all *Catonebria* (except *Nebria baumanni* Kavanaugh, 2015) the medial margins of the elytra diverge apically and a distinct angle. Internally, they are distinguished in having males with the right paramere of the aedeagus very long and slender and females with a bursa copulatrix with a pair of large dorsal paramedial sclerotized plates in the vestibular chamber and a very long spermathecal duct. Males of *Neaptenonebria*, *Palaptenonebria* and *Catonebria* have the right paramere slightly or moderately shorter, and females have a bursa without sclerites in the vestibular chamber and with a shorter spermathecal duct. The right lateral face of the mid-shaft of the median lobe of *Nivalonebria* males is smoothly convex and lacks any of the depressions or invaginations seen in this region in *Neaptenonebria*, *Palaptenonebria*, and some *Catonebria* males.

##### Known distribution and diversity.

The geographical range of this clade includes two disjunct areas of western North America, each with a single endemic species: (1) the northern Cascade Range of Washington and northern Oregon, from North Cascades National Park in the north to Mount Hood in the south (with *N. paradisi* endemic to that area); and (2) the Trinity Alps of northwestern California (with *N. turmaduodecima* endemic there).

#### Subgenus Neaptenonebria

Taxon classificationAnimaliaColeopteraCarabidae

Kavanaugh, subgen. nov.

6F8FB7FB-DD9C-5589-AD6B-E214F38A097F

http://zoobank.org/5CEF95B0-A9A1-48D3-A718-933B50FA7237

= “ovipennis group” (in part) sensu Lindroth 1961.
Catonebria Shilenkov, 1975 (in part); = “kincaidi group” sensu Ledoux and Roux 2005.

##### Type species.

*Nebria ovipennis* LeConte, 1878:477, by present designation.

##### Diagnosis.

Body size small to medium, SBL = 9.4 to 12.8 mm. Head width medium or slightly to markedly broadened, not constricted behind eyes; vertex with a pair of paramedial pale spots on vertex and one pair of supraorbital setae. Eyes moderate in size or slightly reduced, moderately convex. Antennae slightly to moderately elongate; antennal scape with one subapicodorsal seta; antennomeres 3 and 4 not laterally compressed, without extra setae. Labrum with three pairs of apical setae. Maxillary stipes typical for genus, with setae inserted flush on smooth surface. Penultimate labial palpomere with three setae (except only two present in *Nebria carri* Kavanaugh, 1979 specimens). Pronotum markedly to extremely cordate in dorsal aspect, with lateral margination faintly impressed and slightly narrow at middle; one midlateral seta and one basolateral seta present on each side (except basolateral setae absent from *N. carri*, *Nebria balli* Kavanaugh, 1979 and *Nebria kincaidi* Schwarz, 1900 specimens). Elytral intervals smooth, moderately convex, without macrosculpture; elytral interval 3 with two to seven setiferous pores, interval 5 with zero or one setiferous pore, and interval 7 with zero to two or more setiferous pores; umbilicate series comprised of from ten to 17 setae; intervals bearing setae faintly to moderately catenate. Hindwings reduced to short, slender strap-like vestiges. Metasternum very markedly to extremely short. Metepisterna smooth, impunctate. Protarsomeres 1–3 expanded in males (except all five tarsomeres expanded in males from some populations of *N. ovipennis* LeConte, 1878); mesotarsomeres 2–4 longer than their apical width (except expanded in specimens from some populations of *N. ovipennis*); tarsi dorsally glabrous. Abdominal sternites IV–VI with from one (seen only in some specimens of *N. balli*) to five or more pairs of posterior paramedial setae and with from zero to five pairs of posterior paralateral setae; sternite VII with one pair of paramedial apical setae in males, two pairs in females. Median lobe of male aedeagus sclerotized dorsally at least to midlength on shaft, symmetrical to slightly deflected right in dorsal aspect; basal bulb expanded, quadrate, broadly open basally and closed dorsally, without a sagittal aileron present at base or with only a lightly sclerotized collar; mid-shaft parallel-sided in lateral aspect, slightly to moderately compressed in cross-section, with right lateral face with a distinctly and deeply invaginated pouch; apical orifice extremely deflected right. Right paramere narrow and slightly shortened. Female hemisternites VIII with basal apodeme very deeply emarginated. Female valvifer without vestiture; gonopods VIII fused to dorsomedial bases of gonocoxae; gonocoxae with ventral diagonal row of setiform setae and mediodorsal row of setae present, mediodorsal row oriented obliquely relative to longitudinal axis of gonocoxa. Bursa copulatrix without dorsal sclerites in vestibular chamber and without a posterodorsal extension from its longitudinal axis in lateral aspect; spermathecal chamber broadly cordate in dorsal aspect, without dorsal sclerites (in most species) or with a simple flat plate on dorsal surface of an accessory lobe (in *N. kincaidi* and *N. balli*); spermathecal duct medium to slightly short in length and of uniform diameter throughout or nearly so, inserted basodorsally on spermathecal chamber or on a dorsal accessory lobe of that chamber; spermathecal duct slightly short to medium length; spermathecal reservoir of medium length.

##### Etymology.

The subgeneric epithet is a noun of feminine gender and combination of the Greek word, *neos*, meaning new, in reference to the Nearctic (New World) distribution of the group, the Greek word, *aptenos*, meaning unable to fly, and the genus name, *Nebria*, in reference to the observation that all known members of this clade have extremely reduced hindwings incapable of supporting flight.

##### Remarks.

Members of this group are easily identified as members of the *Catonebria* Complex using the Ledoux and Roux’s (2005) key to subgenera. Features that distinguish them from members of subgenus Nivalonebria have been discussed above for that taxon. We have not yet identified any external morphological feature that consistently and satisfactorily distinguishes all members of all species of *Neaptenonebria* from all *Catonebria* members. The hindwings are more highly reduced (to short, slender strap-like vestiges) in all *Neaptenonebria* members than in any *Catonebria* member. The most highly reduced hindwings seen among *Catonebria* species are in *Nebria pektusanica* Horvatovich, 1973) from Changbei Shan (named Mt. Pektusan in North Korea), and they are short and lobate with some recognizable venation present. Features associated with wing loss in carabids, especially *Nebria*, are poor indicators of relationship, but they can still serve to aid identification. Internally, *Neaptenonebria* males have the right paramere of the aedeagus narrowed and slightly shorter than is typical for *Nebria* males, including those of *Catonebria*, but the difference is not dramatic. All males of *Neaptenonebria* species have the right lateral face of the mid-shaft region of the aedeagal median lobe with a distinct longitudinal groove, the basal end of which is invaginated as a moderately (as in *N. ovipennis* males) to deeply invaginated and basally-directed pouch (as in *N. carri* males). Presence of an invaginated pouch is distinctive for this clade; but males of *Palaptenonebria* species and *Catonebria* members of the *gebleri* group, as well as of *N. metallica* Fischer von Waldheim, 1822 and *Nebria labontei* Kavanaugh, 1984, also have a shallow, more or less distinct longitudinal groove in this location. *Neaptenonebria* females have the mediodorsal row of setae on the gonocoxae oriented obliquely in relation to the longitudinal axis of the gonocoxa, whereas this setal row is parallel to the longitudinal axis in females of other subgenera of the *Catonebria* Series.

A few additional features distinguish members of this clade from most members of the *Palaptenonebria* clade. Most adults of *Neaptenonebria* species are medium-sized with SBL > 10 mm, whereas all *Palaptenonebria* adults are smaller, with SBL ≤ 9.5 mm; but smallest the members of the former clade (SBL = 9.4 mm) overlap in size with the largest members of the latter (SBL = 9.5 mm). In *Neaptenonebria* species, the pronotal shape is more extremely cordate with the lateral pronotal margination more faintly impressed and less narrowed at the middle than in *Palaptenonebria* species. The elytra are more convex in *Neaptenonebria* species than in *Palaptenonebria* species, and the elytral umbilicate series is comprised of 11–17 setae in *Neaptenonebria* species but only 8–12 setae in *Palaptenonebria* species. The length of the metasternum is greatly reduced in both of these clades but more extremely so among *Neaptenonebria* adults. Most members of all species of the *Neaptenonebria* clade except *N. ovipennis* have paralateral setae on sternites IV–VI, and, when present, these setae are inserted much nearer to the posterior than the anterior margin of the sternite. All specimens examined of several species of the *Palaptenonebria* clade lack paralateral setae and, in the other species and those individuals in which these setae are seen, they are inserted in a more anterior position on the ventrites. In all *Neaptenonebria* species, all males examined have a single pair of apical paramedial setae on sternite VII and females have two pairs of these setae, whereas in all *Palaptenonebria* species at least some if not most or all males have two or more pairs of setae and at least some females have three or more pairs of setae. Finally, the basal apodemes of the female hemisternites VIII are more deeply emarginate in all *Neaptenonebria* females than in any *Palaptenonebria* females.

##### Known distribution and diversity.

The geographical range of this clade is restricted to western North America. It includes six species that together occupy a disjunct geographical distribution, with three species endemic to the Sierra Nevada of California and westernmost Nevada, one endemic to the mountains of Central Idaho and the Bitterroot Mountains of westernmost Montana, one to the Cascade Range of southern Washington and northern Oregon, and one to the western slope of Coast Ranges of southeastern Alaska and British Columbia south to Vancouver Island and the Olympic Peninsula of Washington.

#### Subgenus Palaptenonebria

Taxon classificationAnimaliaColeopteraCarabidae

Kavanaugh, subgen. nov.

6CE79A2D-0ED2-5B82-8439-A36BD3B559F6

http://zoobank.org/4C1DE2A8-7672-432F-B7D5-35E513913BD7

Catonebria Shilenkov, 1975 (in part); = “mellyi” group sensu Ledoux and Roux 2005.

##### Type species.

*Nebria mellyi* Gebler, 1847:312, by present designation.

##### Diagnosis.

Body size small, SBL = 7.2 to 9.5 mm. Head width medium or slightly broadened, not constricted behind eyes; vertex with a pair of paramedial pale spots on vertex and one pair of supraorbital setae. Eyes slightly to moderately reduced in size, moderately convex. Antennae medium length to slightly elongate; antennal scape with one subapicodorsal seta (or two in *Nebria roddi* Dudko & Shilenkov, 2001 and *Nebria baenningeri korgonica* Dudko & Shilenkov, 2001); antennomeres 3 and 4 not laterally compressed, without extra setae. Labrum with three pairs of apical setae. Maxillary stipes typical for genus, with setae inserted flush on smooth surface. Penultimate labial palpomere with three setae. Pronotum moderately cordate in dorsal aspect, with lateral margination shallowly to moderately impressed and markedly narrow at middle; one midlateral seta and one basolateral seta present on each side. Elytral intervals smooth, slightly to moderately flattened, without macrosculpture, elytral interval 3 with one to seven setiferous pores, intervals 5 and 7 with zero to two setiferous pores; umbilicate series comprised of eight to ten setae; intervals bearing setae faintly to moderately catenate. Hindwings reduced to short, slender strap-like vestiges. Metasternum moderately to markedly short. Metepisterna smooth, impunctate. Protarsomeres 1–3 expanded in males; mesotarsomeres 2–4 longer than their apical width; tarsi dorsally glabrous (or with few sparse and fine setae in *Nebria mellyi* Gebler, 1847 specimens). Abdominal sternites IV–VI with from two to five or more pairs of posterior paramedial setae and with zero to three pairs of anterior paralateral setae; sternite VII with one or two pairs of paramedial apical setae in males, two to three or more pairs in females. Median lobe of male aedeagus sclerotized dorsally at least to midlength on shaft, symmetrical to slightly deflected right in dorsal aspect; basal bulb expanded, quadrate, broadly open basally and closed dorsally, without a sagittal aileron present at base or with only a lightly sclerotized collar; mid-shaft parallel-sided in lateral aspect, slightly to moderately compressed in cross-section, right lateral face with a shallow longitudinal groove or shallow indentation; apical orifice moderately (in *Nebria lyubechanskii* Dudko, 2008 specimens only) or extremely deflected right. Right paramere narrow and slightly shortened. Female hemisternites VIII with basal apodeme faintly to moderately emarginated. Female valvifer without vestiture; gonopods VIII fused to dorsomedial bases of gonocoxae; gonocoxae with ventral diagonal row of setiform setae and mediodorsal row of setae present, mediodorsal row oriented parallel to longitudinal axis of gonocoxa. Bursa copulatrix without dorsal sclerites in vestibular chamber and without a posterodorsal extension from its longitudinal axis in lateral aspect; spermathecal chamber broadly cordate in dorsal aspect, without dorsal sclerites (in most species) or with a simple, broad and convoluted plate anterior to spermathecal duct insertion (in *Nebria sajana sarlyk* Dudko & Shilenkov, 2001, *N. s. dubatolovi* Dudko & Shilenkov, 2001 and *N. s. sitnikovi* Dudko & Shilenkov, 2001); spermathecal duct slightly short in length and of uniform diameter throughout or nearly so, inserted basodorsally on spermathecal chamber or on a dorsal accessory lobe of that chamber (in *Nebria sajana sajana* Dudko & Shilenkov, 2001); spermathecal reservoir of medium length.

##### Etymology.

The subgeneric epithet is a noun of feminine gender and combination of the Greek word, *palaios*, meaning old, in reference to the Palearctic (Old World) distribution of the group, *aptenos*, meaning unable to fly, and the genus name, *Nebria*, in reference to observation that all known members of this clade have extremely reduced hindwings incapable of supporting flight.

##### Remarks.

As for members of subgenus Neaptenonebria, those of this group are easily identified as members of the *Catonebria* Complex using the Ledoux and Roux’s (2005) key to subgenera. Features that distinguish them from members of subgenus Nivalonebria have been discussed above for that taxon. We cannot suggest any external morphological feature that consistently and satisfactorily distinguishes all members of all species of *Palaptenonebria* from all *Catonebria* members, and there are few uniquely distinguishing internal features evident as well. The hindwings are more highly reduced (to short, slender strap-like vestiges) in all *Palaptenonebria* members than in any *Catonebria* member, but are similar in size to those of *Neaptenonebria* species. Internally, *Palaptenonebria* males have the right paramere of the aedeagus narrowed and slightly shorter than those of *Catonebria* males, but, again, similar in form to those in *Neaptenonebria* males. All males of *Palaptenonebria* species have the right lateral face of the mid-shaft region of the aedeagal median lobe modified to some extent. There is at least a shallow longitudinal depression or groove present in this region, and in *N. mellyi* that groove is deeply impressed and deepest and most sharply delineated basally. However, in no males of this clade is there an invaginated, basally-directed pouch as seen in all *Neaptenonebria* males. As noted above, a groove or depression similar to that seen in *Palaptenonebria* males is found also in *Catonebria* males of the *gebleri* group, as well as of *N. metallica* and *Nebria labontei*. In *Palaptenonebria* females, the mediodorsal row of setae on the gonocoxae is present and oriented parallel to the longitudinal axis of the gonocoxa rather than obliquely in relation to the longitudinal axis as in Neaptenonebria females. See the Discussion section above for Neaptenonebria for a few additional distinguishing features or trends.

##### Known distribution and diversity.

The geographical range of this clade is restricted to the Altai-Sayan region of southern Siberia, including the Altai, Western Sayan and Tannu-Ola Mountain systems, and presently includes six taxa ranked as species and another eight ranked as subspecies. Dudko and Shilenkov (2001) provided an excellent revision of this group.

## Future Research

A better understanding of relationships among nebriite carabids should be achieved with additional taxon sampling for molecular data. Atop the list of taxa that remain unsampled are *Notiokasis* and *Archileistobrius*. Molecular data should allow us to confirm whether or not the former is a member of this clade and just how the latter is related to other Nebriini. Within genus *Nebria*, several groups remain inadequately sampled. This is especially true for the diverse *Nebria* Complex, including the currently non-monophyletic subgenus Nebria s. str. The addition of species from any part of the range of this subgenus would be useful, but especially those from the Caucasus Mountains region and Asia Minor, the Balkan and Italian Peninsulas, and the Carpathian Mountains system. Subgenera *Tyrrhenia* and *Alpaeonebria* are also under-represented in our sample, and each includes groups that, together, might not comprise a single clade. Our sample did not include any representatives of subgenus Boreonebria from the southern part of its range, across mid-latitudes in Asia from North Korea to Kazakhstan. Consequently, relationships of species from these areas to other members of the subgenus remain unclear. Additional sampling within *Epinebriola*, including additional western and central Himalayan species and species currently assigned to *Patrobonebria*, could help to clarify relationships within that clade. Data from additional *Eunebria* species should help determine whether *Eurynebria* is sister to *Eunebria* or just where within the latter it is nested. Although we do not anticipate that the sampling of DNA from additional *Archastes* species will alter the relationships of this group to other *Nebria* suggested by this study, genetic diversity within the group deserves further examination. It is not yet clear how the central Asian and Far East Asian *Eonebria* species are related, whether they represent distinct clades or if they share more complicated relationships. Additional samples from both of these areas could help resolve this question. Finally, although sampling of the *Reductonebria* and *Catonebria* Complexes has been extensive, a few species not included in our sample remain of interest. Among *Reductonebria*, *N. angustula* from the Kamchatka Peninsula, Russia and *N. nicolasi* from the Qionglai Mountains of central Sichuan Province, China exhibit some morphological features atypical for the subgenus. Among *Catonebria*, *N. scaphelytra* and *N. suensoni* are outliers, morphologically and geographically, respectively. Adding these taxa to the molecular taxon sample should help to clarify phylogenetic relationships for each of them and for the subgenera to which they belong.

Many of the species yet to be sampled for DNA are from areas not easy or even possible to visit safely for collecting fresh material, and others are known only from one or a few specimens deposited in collections. The use of high-throughput and ancient DNA sequencing technologies offers an opportunity to access DNA non-destructively from dried and pinned specimens of many of these species. Future research should make use of this source of new DNA sequence data whenever possible.

We have not yet been able to carry out a molecular clock analysis of the nebriite phylogenetic tree using our data. Recent additions to the fossil record for nebriites may allow us to better estimate dates for at least some major nodes in the tree, but this work remains for the future.

Much classical taxonomic work using morphology is also still ahead. Many of the clades we recognize based on analyses of DNA sequence data currently have few if any concordant morphological synapomorphies that support them. A re-examination of internal and external morphology of members of these new clades in particular may yield new synapomorphic features previously overlooked, or perhaps also from new character systems, if we focus our sampling on these clades and their boundaries. The re-examination of morphological features is particularly important among members of the *Epinebriola* and *Eunebria* species that were unavailable to use for this study. For each of these subgenera, groups of species formerly assigned to them were found to be more closely related to other subgenera. *Nebria delicata* and *N. retingensis* have been transferred from *Epinebriola* to subgenus Parepinebriola subgen. nov. Outstanding questions include which, if any, of the species still included in *Epinebriola* should also be transferred to *Parepinebriola*, and do the morphological features tentatively recognized as distinguishing the two subgenera hold true? Similarly, three members of the *przewalskii* group of *Eunebria* have been shown instead to be members of *Psilonebria.* Do the other members of the *przewalskii* group not examined in our study, or perhaps those other species still included in *Eunebria*, share the morphological features now associated with *Psilonebria*? Re-examination of morphological features in these groups may help to answer these questions.

Additional morphological characters are also needed before we can generate useful keys for the identification of the subgeneric series, complexes and subcomplexes and even some of the subgenera within *Nebria* as we have proposed. Characters used in these keys will need to be well illustrated.

We have noted and lamented the general inability of the results of our analyses of molecular data to resolve clearly phylogenetic relationships among many of the terminal taxa in our species trees. The divergences of many of these taxa are associated with very short branch lengths, suggesting that they are relatively recent events. In addition, we have used single specimens to represent all but a few of the species-group taxa in our sample. Recent phylogeographic studies by Schoville et al. (2012) of several *Nebria* species in the Sierra Nevada of California and Weng et al. (2020) of species of Nippononebria subgenus Vancouveria in western North America have shown just how complex the relationships of species and populations can appear when larger samples and samples from throughout the geographical ranges of each taxon are included in analyses. In general, they have confirmed species concepts developed using morphological data. Phylogeographic analyses among species groups appear to us to offer the means to understand better the relationship between species trees and gene trees and to reconcile molecular results with those of morphology among closely related species.

## Supplementary Material

XML Treatment for Parepinebriola

XML Treatment for Insulanebria

XML Treatment for Erwinebria

XML Treatment for Nivalonebria

XML Treatment for Neaptenonebria

XML Treatment for Palaptenonebria

## Appendix A. Locality data for specimens that yielded DNA sequences for this study

**Table:** For each specimen, DNA voucher number is listed under “DNA ID” and its depository is listed under “Dep” (AF = A. Faille collection; CAS = California Academy of Sciences; JS = J. Schmidt collection; KS = K. Sasakawa collection; OSAC = Oregon State University Arthropod Collection; SDS = S.D. Schoville collection).

Taxon	DNA ID	Locality	Dep
*Leistus birmanicus*	DHK0084	China, Yunnan, Gongshan County, Dulongjiang Township, Bapo, Miliwang, 1956 m, 27.72383°N, 098.36117°E	CAS
*Leistus crenatus*	DHK1939	Algeria, Central Djurdjura, Anou Boussouil, 1718 m, 36.4690°N, 4.1914°E	CAS
*Leistus ferrugineus*	DHK1903	Italy, Piedmont, Cuneo, Po River Valley at Paesano, 618 m, 44.67584°N, 7.27709°E	CAS
*Leistus ferruginosus*	DHK0336	USA, Oregon, Benton County, Alsea Falls, 326 m, 44.31949°N, 123.49750°W	CAS
*Leistus frateroides*	SDS13-123A	Russia, Altai Republic, Evrechala Mt., 1891 m, 51.47490°N, 087.42992°E	CAS
*Leistus fulvibarbis*	DHK1937	France, Eure, La Saussaye, 49.25326°N, 0.99201°E	CAS
*Leistus fulvus*	DHK1938	Azerbaijan, Qax, forest litter in road to Qax, 580 m, 41.39828°N, 46.92153°E	CAS
*Leistus gaoligongensis*	DHK0340	China, Yunnan, Longyang County, Luoshuidong, 2308 m, 24.94833°N, 98.75667°E	CAS
*Leistus kryzhanovskii*	SDS13-330A	Russia, Altai Republic, Aigulaksky Range, 2555 m, 50.44896°N, 087.40025°E	CAS
*Leistus lihengae*	DHK0343	China, Yunnan, Longyang County, Luoshuidong, 2308 m, 24.94833°N, 98.75667°E	CAS
*Leistus longipennis*	DHK0338	USA, California, Del Norte County, 8.3 miles N of Crescent City, 18 m, 41.86559°N, 124.13906°W	CAS
*Leistus madmeridianus*	DHK0339	USA, California, Sonoma County, Salt Point State Park, 91 m, 38.56823°N, 123.31916°W	CAS
*Leistus niger*	DHK1761	Russia, Republic of Khakassia, Karasibo River, 750 m, 52.30713°N, 90.12761°E	CAS
*Leistus niitakaensis*	DHK1800	Taiwan, Chiayi County, Alishan District, west slope Yu Shan, 3429 m, 23.46641°N, 120.95041°E	CAS
*Leistus nitidus*	DHK1888	France, Hautes-Alpes, Ristolas, 1643 m, 44.77196°N, 6.95399°E	CAS
*Leistus nokoensis*	DHK1834	Taiwan, Tiachung City, Xueshan, Black Forest, 3340 m, 24.39359°N, 121.24936°E	CAS
*Leistus nubivagus*	DHK0502	Canary Islands, Tenerife, La Victoria, Barranco del Madroño, 1435 m	CAS
*Leistus parvicollis*	DHK2107	Greece, Kefallonia, Enos, 1000–1600 m	CAS
*Leistus pyrenaeus*	DHK1941	Spain, Lleida, Llastarri, Minas de Canal	CAS
*Leistus rufomarginatus*	DHK1394	Belgium, West Flanders, Wijnendale Forest	CAS
*Leistus sardous*	DHK1942	Italy, Sicily, Piana degli Albanesi – Grotta del Garrone	AF
*Leistus smetanai*	DHK1860	Taiwan, Taichung City, Xueshan, Black Forest, 3460 m, 24.39373°N, 121.25114°E	CAS
*Leistus* sp JIL 2	SDS12-202	China, Jilin, northern slope of Changbai Shan, 1772 m, 42.04913°N, 128.05782°E	CAS
*Leistus* sp NEP	DHK2108	Nepal, Lapchi Kang range, Chera, 4400 m, 27.89861°N, 86.06278°E	CAS
*Leistus* sp SCH	DHK0634	China, Sichuan, Litang County, Shalulishan, Haizishan Yakou, 4610 m, 29.47366°N, 100.21921°E	CAS
*Leistus* sp YUN 1	DHK0015	China, Yunnan, Gongshan County, Heiwadi, 2070 m, 27.79650°N, 98.58267°E	CAS
*Leistus* sp YUN 2	DHK0224	China, Yunnan, Shibali Yakou, second cirque, 3700 m, 27.20333°N, 98.69303°E	CAS
*Leistus taiwanensis*	DHK1838	Taiwan, Nantou County, Tatajia at Dongpu Cabin, 2563 m, 23.48438°N, 120.88591°E	CAS
*Nebria acuta*	DHK0018	USA, Alaska, Wrangell Mts., Kennicott, National Creek, 615 m, 61.48469°N, 142.88696°W	CAS
*Nebria aenea*	DHK1445	Russia, Altai Krai, Charyshsky District, Altai Mts., Gorelyi, Korgon River, 1000 m, 53.0°N, 83.8°E	CAS
*Nebria albimontis*	SDS05-018	USA, California, Mono County, North Fork Perry Eiken Creek, 3278 m, 37.63879°N, 118.22910°W	CAS
*Nebria altaica*	DHK0404a	Russia, Buryat Republic, Tunka Mts., East Branch Kingarga River, 1500 m, 51.97275°N, 102.45970°E	CAS
*Nebria angustata*	DHK1653	Switzerland, Bern, Guttannen, Furtwangsattel	CAS
*Nebria angusticollis*	DHK1896	France, Hautes-Alpes, Col d’Agnel, 2704 m, 44.68499°N, 6.97789°E	CAS
*Nebria appalachia*	DHK0507	USA, Tennessee, Sevier County, Great Smoky Mountains National Park, Ramsey Prong, 1084 m, 35.7088°N, 83.3129°W	CAS
*Nebria arinae*	SDS12-063E	Russia, Altai Republic, Holzun Mts., 2162 m, 50.20821N°, 084.55408°E	CAS
*Nebria arkansana*	DHK0838	USA, Colorado, Summit County, Quandary Peak, 3763 m, 39.39159°N, 106.11371°W	CAS
*Nebria austriaca*	DHK1654	Austria, Lower Austria, The Oscher	CAS
*Nebria baenningeri*	SDS12-025A	Russia, Altai Republic, Holzun Mts., 2018 m, 50.31269°N, 084.61154°E	CAS
*Nebria baicalica*	DHK0386a	Russia, Irkutsk Oblast, Lake Baikal at Bolschie Koty, 450 m, 51.90405°N, 105.08014°E	CAS
*Nebria balli*	DHK0930	USA, Washington, Mt. Rainier National Park, Paradise area, Edith Creek Basin, 1807 m, 46.79678°N, 121.72711°W	CAS
*Nebria banksii*	DHK0406	Russia, Kamchatka, Avancha River, 11 m, 53.18297°N, 158.39328°E	CAS
*Nebria baumanni*	DHK1814A	USA, Nevada, Clark County, Spring Mts., Deer Creek, 2570 m, 36.31018°N, 115.62334°W	CAS
*Nebria bellorum*	DHK0510	USA, Tennessee, Sevier County, Great Smoky Mountains National Park, Middle Fork Little Pigeon River, 630 m, 35.7031°N, 83.3581°W	CAS
*Nebria beverlianna*	DHK0804	USA, Wyoming, Sublette County, Hoback River, 1930 m, 43.27364°N, 110.52606°W	CAS
*Nebria boschi*	DHK1509	Germany, Baden-Wüttemberg, Tübingen, Bad Urach, Höhle, 670 m, 48.480°N, 9.368°E	CAS
*Nebria bremii*	DHK1648	Switzerland, Uri, Schlossberglücke	CAS
*Nebria brevicollis* OR	DHK0717	USA, Oregon, Polk County, 1.5 miles W of Dallas, 160 m, 44.90748°N, 123.35466°W	CAS
*Nebria brevicollis* SP	DHK0009	Spain, Albacete, Peñascosa, 38.67305°N, 2.40324°E	CAS
*Nebria businskyorum*	DHK0676	China, Xizang, Medog, Baibung, E of Doxong Pass, 3937 m, 29.48748°N, 94.95791°E	CAS
*Nebria calva*	DHK0430a	USA, Arizona, Apache County, West Fork Little Colorado River, 2814 m, 33.95821°N, 109.51614°W	CAS
*Nebria capillosa*	DHK2029	Nepal, Dolpo district, Rupghat Khola valley E of Juphal, 2150–2500 m, 29.97°N, 82.85°E	CAS
*Nebria carbonaria*	DHK0459a	Russia, Kuril Islands, Paramushir, inland from Severo-Kurilsk, east slope Ebeko Volcano, 50.67533°N, 156.09550°E	CAS
*Nebria carri*	DHK0208	USA, Idaho, Camas County, Carrie Creek, 2426 m, 43.59555°N, 114.69264°W	CAS
*Nebria cascadensis*	DHK0899	USA, Washington, Whatcom County, North Fork Nooksack River, 222 m, 48.90471°N, 121.99183°W	CAS
*Nebria castanea*	DHK1519	Italy, Trentino-Alto, Passo di Gávia, 2547 m, 46.35983°N, 10.50054°E	CAS
*Nebria castanipes*	DHK0001	Canada, British Columbia, Alexander Creek, 3.6 miles W of Crowsnest Pass, 1308 m, 49.65580°N, 114.73222°W	CAS
*Nebria catenata*	DHK1067	USA, Utah, Grand County, La Sal Mts., Mill Creek, 2545 m, 38.50914°N, 109.28248°W	CAS
*Nebria catenulata*	DHK0405a	Russia, Buryat Republic, Tunka Mts., East Branch Kingarga River, 1500 m, 51.97275°N, 102.45970°E	CAS
*Nebria changaica*	DHK1451	Russia, Altai Republic, Altai Mts., Ukok Upland near Murzdy-Bulak Lake, 2700–3100 m, 49.28°N, 87.65°E	CAS
*Nebria charlottae*	DHK1254	Canada, British Columbia, Haida Gwaii, Graham Island, north shore at Tow Hill, 3 m, 54.07382°N, 131.66662°W	CAS
*Nebria chinensis*	DHK0600	China, Shaanxi, Zhouzhi County, Houzhenzi village, 1271 m, 33.85190°N, 107.83776°E	CAS
*Nebria chuskae*	DHK0437a	USA, Arizona, Apache County, Lukachukai Creek, 2200 m, 36.44443°N, 109.17993°W	CAS
*Nebria coloradensis*	DHK0852	USA, Colorado, Lake County, Lake Creek, 2927 m, 39.06532°N, 106.42093°W	CAS
*Nebria complanata*	DHK1949	Italy, Grosseto, Parco Regionale della Maremma, Monti dell’Uccellina, 42.63284°N, 11.07733°E	CAS
*Nebria coreica*	DRM4721	Russia: Primorsky Krai, 20 km W of Nachodka	CAS
*Nebria crassicornis*	DHK0021	USA, Washington, Olympic National Park, Hurricane Ridge, 1580 m, 47.97166°N, 123.49000°W	CAS
*Nebria dabanensis*	DHK0402b	Russia, Buryat Republic, Khamar-Daban Mts., Baikalsky Preserve, 1803 m, 51.34774°N, 105.13042°E	CAS
*Nebria danmanni*	DHK1134	USA, Washington, Olympic National Park, Mount Olympus, Snow Dome, 2050 m, 47.81165°N, 123.70196°W	CAS
*Nebria darlingtoni*	DHK0023	USA, California, El Dorado County, South Fork American River, 934 m, 38.76730°N, 120.48415°W	CAS
*Nebria delicata*	DHK2082	China, Xizang, Mt. Mendju Zari SE of Lhasa, 5200–5420m, 29.57°N, 91.22°E	JS
Nebria cf. desgodinsi	DHK2026	Nepal, Solu Khumbu district, near Kothe (Mosom Kharka), Inkhu Khola, 3600–3700 m, 27.67°N, 86.83°E	CAS
*Nebria desolata*	DHK0450a	USA, Utah, Garfield County, 6.0 miles NE of Henrieville, Dry Creek, 1971 m, 36.44443°N, 109.17993°W	CAS
*Nebria diaphana*	DHK1652	Italy, Trentino, Passo di Brocon	CAS
*Nebria diversa*	DHK0014	USA, Oregon, Lincoln County, 2 miles N of Newport at Moolack Beach, 6 m, 44.70572°N, 124.06115°W	CAS
*Nebria dubatolovi*	SDS12-092A	Russia, Altai Republic, Katunskii Mts., 2419 m, 50.00761°N, 086.25175°E	CAS
*Nebria edwardsi*	DHK0025	USA, Montana, Mineral County, Trout Creek, 13.4 miles NE of Hoodoo Pass, 1006 m, 47.07933°N, 114.92120°W	CAS
*Nebria edwardsi*	DHK0463a	USA, Montana, Mineral County, Trout Creek, 13.4 miles NE of Hoodoo Pass, 1007 m, 47.07933°N, 114.92120°W	CAS
*Nebria eschscholtzii*	DHK0022	USA, California, Somona County, Mark West Creek, 142 m, 38.54291°N, 122.72066°W	CAS
*Nebria fontinalis*	DHK1536	Switzerland, Uri, Oberalp, 2036 m, 46.66236°N, 8.66794°E	CAS
*Nebria formosana*	DHK1592	Taiwan, Taichung City, Heiping District, Xue Shan, Black Forest, 3460 m, 24.39372°N, 121.25114°E	CAS
*Nebria fragariae*	DHK0996	USA, Oregon, Grant County, Strawberry Creek, 1750 m, 44.31951°N, 118.67473°W	CAS
*Nebria fragilis*	DHK0028	USA, Utah, Utah County, South Fork American Fork River, 2207 m, 40.43519°N, 111.63603°W	CAS
*Nebria frigida*	DHK0005	USA, Alaska, Wrangell Mts., Kennicott, National Creek, 615 m, 61.48469°N, 142.88696°W	CAS
*Nebria fulgida*	DHK0409a	Russia, Buryat Republic, Tunka Mts., Kingarga River, 1380 m, 51.97478°N, 102.41099°E	CAS
*Nebria fulviventris*	DHK1397	Italy, Tuscany, Vallombrosa, 43.733°N, 11.557°E	CAS
*Nebria gagates*	DHK1880	France, Hautes-Alpes, Ristolas, Torrent de Clot Lucette, 1740 m, 44.76780°N, 6.94974°E	CAS
*Nebria gebleri*	DHK0002	USA, Alaska, Chichagof Island, Goulding River, 23 m, 57.77752°N, 136.25931°W	CAS
*Nebria georgei*	DHK0501	USA, Arizona, Grand Canyon National Park, Colorado River Mile 180, 36.187°N, 113.106°W	CAS
*Nebria germari* AU	DHK1645	Austria, Upper Austria, Höllengebirge, Höllkogel	CAS
*Nebria germari* GE	DHK1646	Germany, Bavaria, Allgäuer Alps, Ifen	CAS
*Nebria giulianii*	SDS06-031B	USA, California, Inyo County, Milner Creek, 2241 m, 37.59480°N, 118.29763°W	CAS
*Nebria gouleti*	DHK0006	USA, Idaho, Idaho County, Selway River, 7.5 miles SE of Lowell, 475 m, 46.08804°N, 115.50410°W	CAS
*Nebria gregaria*	DHK0017	USA, Alaska, Aleutian Islands, Chuginadak Island, north shore, 10 m, 52.88447°N, 169.74653°W	CAS
*Nebria gyllenhali*	DHK0010	Russia, Buryat Republic, Khamar-Daban Mts., Tankhoy, 512 m, 51.53806°N, 105.112049°E	CAS
*Nebria haida*	DHK1300	Canada, British Columbia, Haida Gwaii, Moresby Island, ridge N of Takakia Lake, 837 m, 52.93787°N, 132.04862°W	CAS
*Nebria holzunensis*	SDS12-033D	Russia, Altai Republic, Holzun Mts., 1981 m, 50.25637°N, 084.59192°E	CAS
*Nebria hudsonica*	DHK0381a	USA, Wyoming, Sublette County, Hoback River, 1956 m, 43.24509°N, 110.48779°W	CAS
*Nebria ingens*	SDS08-025G	USA, California, Inyo County, North Fork Big Pine Creek, 3446 m, 37.11192°N, 118.51174°W	CAS
*Nebria intermedia* OR	DHK0990	USA, Oregon, Baker County, Elkhorn Range, Anthony Lake, 2182 m, 44.96122°N, 118.23200°W	CAS
*Nebria intermedia* UT	DHK1082	USA, Utah, Sevier County, 8.2 miles SE of Monroe, 2707 m, 38.55808°N, 112.08167°W	CAS
*Nebria intermedia* WY	DHK0809	USA, Wyoming, Teton County, Togwotee Pass, 2910 m, 43.75231°N, 110.06836°W	CAS
*Nebria japonica*	DHK1371	Japan, Honshu, Miyagi Prefecture, Road 12 E of Mt. Zao, 1462 m,	CAS
*Nebria jeffreyi*	DHK0039	USA, Oregon, Harney County, Steens Mts., Little Blitzen River, 2663 m, 42.69593°N, 118.59060°W	CAS
*Nebria jockischi*	DHK1526	Switzerland, Valais, Talgletscher Glacier, 2316 m, 46.45237°N, 7.83339°E	CAS
*Nebria kagmara*	DHK2120	Nepal, Karnali, Dolpo District, Kagmara Lekh below Kagmara La, 4400 m	CAS
*Nebria kazabi*	DHK1795	Russia, Republic of Tuva, Western Tannu-Ola Range, 2625 m, 50.54529°N, 90.99348°E	CAS
*Nebria kincaidi*	DHK0874	USA, Washington, Olympic National Park, Grand Pass, 1750 m, 47.86979°N, 123.35916°W	CAS
*Nebria komarovi*	DRM5171	China [locality unspecified]	CAS
*Nebria korgonica*	DHK1475	Russia, Altai Krai, Charyshsky District, Altai Mts., Gorelyi, Belok Mt., headwaters of Sentelek River, 1900 m, 51.075°N, 83.583°E	CAS
*Nebria labontei*	DHK0984	USA, Oregon, Wallowa County, West Fork Wallowa River below Glacier Lake, 2479 m, 45.16528°N, 117.28163°W	CAS
*Nebria lacustris*	DHK0004	USA, Maryland, Montgomery County, Potomac River at Plummers Island, 17 m, 38.96967°N, 77.18517°W	CAS
Nebria cf. laevistriata	DHK0492	China, Yunnan, Gongshan County, Bingzhongluo Township, 0.9 km N of Chukuai Lake, 4050 m, 27.99075°N, 098.47512°E	CAS
*Nebria lafresnayei*	DHK1946	Spain, Huesca, Villanua, Sinistro (Gouffre El)	CAS
*Nebria lamarckensis*	SDS06-648F	USA, California, Inyo County, side canyon of Pine Creek, 2350 m, 37.37605°N, 118.68400°W	CAS
*Nebria lassenensis*	DHK0220	USA, California, Shasta County, Lake Helen, 2485 m, 40.4689°N, 121.5131°W	CAS
*Nebria laticollis*	DHK1529	France, Haute-Savoie, Vallorcine, NW of Refuge Loriaz, 1962 m, 46.03847°N, 6.90832°E	CAS
*Nebria lewisi*	DHK1378	Japan, Honshu, Tochigi Prefecture, Fujioka, Watarase-sitch	CAS
*Nebria ligurica*	DHK1889	France, Hautes Alpes, 5.5 km SE of St. Véran, 2640 m, 44.66354°N, 6.92478°E	CAS
*Nebria limbigera*	DHK2083	China, Xinjiang, south slope of Wusunshan (= Ketmen) Mts., NNE of Zhaosu, 3080 m, 43.37667°N, 81.23861°E	CAS
*Nebria lindrothi*	DHK0020	USA, Colorado, Mineral County, Wolf Creek, 2 miles W of Wolf Creek Pass, 3180 m, 37.47989°N, 106.78024°W	CAS
*Nebria lituyae*	DHK0016	USA, Alaska, Juneau area, Mount Roberts Trail above treeline, 718 m, 58.29450°N, 134.37492°W	CAS
*Nebria livida*	DHK0007	Russia, Irkutsk Region, Tibielti River at Tunka Valley highway, 664 m, 51.76619°N, 103.24932°E	CAS
*Nebria lombarda*	DHK1400	Italy, Lombardia, Val di Scalve	CAS
*Nebria louiseae*	DHK1278	Canada, British Columbia, Haida Gwaii, Talunkwan Island, north shore, 2 m, 57.45150°N, 131.66662°W	CAS
*Nebria lyelli*	DHK0054	USA, California, Mono County, below Conness Lakes, 3134 m, 37.97600°N, 119.29690°W	CAS
*Nebria lyubechanskii*	SDS13-286A	Russia, Republic of Tuva, Western Tannu-Ola Range, north slope, 2744 m, 50.54470°N, 91.01261°E	CAS
*Nebria macra*	DHK1968	China, Xizang, Nyainqentanglha Shan, N Yangpachem, Bhuda valley, 5000–5200 m, 30.18222°N, 90.48917°E	CAS
*Nebria macrogona*	DHK1377	Japan, Honshu, Yamagata Prefecture, Kaminoyama, Sen’nin-sawa, Mts. Zao	CAS
*Nebria mannerheimii*	DHK0026	USA, Idaho, Idaho County, Salmon River at Riggins, 685 m, 45.32450°N, 116.34933°W	CAS
*Nebria maroccana*	DHK1945	Morocco, Fèz-Meknès, Ifane, Ain Leuh to Azrou, Ifri Mkhrouga	CAS
*Nebria martensi*	DHK2069	Nepal, Jaljale Himal, below Paanch Pokhari, 4000–4100 m, 27.49028°N, 87.46111°E	JS
*Nebria meanyi* CA	DHK1357	USA, California, Siskiyou County, north slope of Mt. Shasta at Bolam Creek, 1850 m, 41.47177°N, 122.23174°W	CAS
*Nebria meanyi* WA	DHK0949	USA, Washington, Mt. Rainier National Park, Van Trump Creek above Christine Falls, 1164 m, 46.78242°N, 121.78021°W	CAS
*Nebria mellyi*	DHK1752	Russia, Kemerovskaya Oblast, Mustag Mt., 1290 m, 53.02153°N, 87.95143°E	CAS
*Nebria mentoincisa*	DHK2017	China, Xizang, Gangdise Shan, Kurum Valley NW Lhasa, SW Namba side valley, 4800–5150 m, 29.67528°N, 90.75444°E	CAS
*Nebria metallica*	DHK0915	USA, Washington, Pierce County, White River at Silver Creek Campground, 802 m, 46.99584°N, 121.53311°W	CAS
*Nebria modoc*	DHK0544	USA, California, Modoc County, Warner Mts., Thoms Creek, 1925 m, 41.56274°N, 120.27076°W	CAS
*Nebria morvani*	DHK0065	Nepal, Karnali, Jumla District, 2 km W of Guthichau, 2800 m, 29.20028°N, 82.30139°E	CAS
*Nebria murzini*	DHK0378a	China, Xinjiang, Tian Shan, Usu, 1500 m	CAS
*Nebria nana*	DHK1952	China, Tibet, S Tanggula Pass, 5000 m, 32.68500°N, 91.87278°E	CAS
*Nebria navajo*	DHK0451a	USA, Arizona, Navajo County, Keet Seel Creek, 1980–2040 m, 36.73423°N, 110.50803°W to 36.75269°N, 110.49601°W	CAS
*Nebria niitakana*	DHK1595	Taiwan, Taichung City, Heiping District, Xue Shan, Black Forest, 3460 m, 24.39372°N, 121.25114°E	CAS
*Nebria niohozana*	DHK1372	Japan, Honshu, Gunma Prefecture,Road 466 W of Shirane-san, 1883 m	CAS
*Nebria nivalis* AK	DHK0388a	USA, Alaska, Katmai National Park, Brooks Lake, 23 m, 58.54625°N, 58.54625°W	CAS
*Nebria nivalis* NU	DHK0387a	Canada, Nunavut, Baffin Island, Glasgow Inlet, Kimmirut, 40 m, 62.84728°N, 69.87410°W	CAS
*Nebria nivalis* gr sp	DHK1802	Russia, Republic of Tuva , Mongun-Taiga, 2490 m, 50.27584°N, 89.93906°E	CAS
*Nebria nudicollis*	DHK1944	Algeria, Central Djurdjura, Takouatz Guerissene, Tijdja à Tizi N´Kouilal	AF
*Nebria numburica*	DHK2075	Nepal, Solu Khumbu district, S of Dudh Kund, 4400–4600 m, 27.70°N, 86.58333°E	CAS
*Nebria obliqua* CO	DHK0436a	USA, Colorado, La Plata County, Durango, Animas River, 1971 m, 37.25852°N, 107.87664°W	CAS
*Nebria obliqua* UT	DHK1104	USA, Utah, Iron County, Parowan Creek, 2.0 miles S of Parowan, 1965 m, 37.81081°N, 112.80845°W	CAS
*Nebria ochotica*	DHK0403a	Russia, Kuril Islands, Paramushir, inland from Krasheninnikova Bay, Krasheninnikova River, 50.28517°N, 155.35650°E	CAS
*Nebria ohdaiensis*	DHK1380	Japan, Honshu Nara Prefecture, Kamikitayama, Mts. Ohdaigahara	CAS
*Nebria olivieri*	DHK2046	France, Ariège, Forêt des Hares, Etang de Laurenti, 1650–2100 m, 42.68°N, 2.03°E	CAS
*Nebria oowah*	DHK1071	USA, Utah, Grand County, La Sal Mts., Oowah Lake, 2678 m, 38.50171°N, 109.27390°W	CAS
*Nebria ovipennis*	DHK0019	USA, California, Tuolumne County, Blue Canyon, 2800 m, 38.315°N, 119.659°W	CAS
*Nebria oxyptera*	DRM5172	China, Xinjiang Uyghur Autonomous Region, Pishan County, Xiadulla	CAS
*Nebria pallipes*	DHK0011	USA, Maryland, Montgomery County, Potomac River at Plummers Island, 17 m, 38.96967°N, 77.18517°W	CAS
*Nebria paradisi* OR	DHK1349	USA, Oregon, Hood River County, Mt. Hood Meadows Ski Area, 1890–2000 m, 45.34272°N, 121.67799°W	CAS
*Nebria paradisi* WA	DHK0929	USA, Washington, Mt. Rainier National Park, Paradise area, Edith Creek Basin, 1807 m, 46.79678°N, 121.72711°W	CAS
*Nebria pasquineli*	DHK0831	USA, Colorado, Boulder County, Lefthand Creek, 2420 m, 40.06590°N, 105.45711°W	CAS
*Nebria pektusanica*	SDS12-197E	China, Jilin, northern slope of Changbai Shan, 1901 m, 42.03954°N, 128.05562°E	CAS
*Nebria perlonga*	DHK0029	China, Xinjiang, Tian Shan, Usu, 1500 m	CAS
*Nebria pertinax*	DHK2119	Nepal, Mahakali/Darchula near Rapla, Shipu Lekh, 4300 m	JS
*Nebria picea*	DHK1649	Switzerland, St. Gallen, Säntis-Lisengrat	CAS
*Nebria picicornis*	DHK1876	France, Hautes Alpes, Aiguilles, Le Guil Torrent, 1454 m, 44.77790°N, 6.85961°E	CAS
*Nebria piperi*	DHK0965	USA, Washington, Mt. Rainier National Park, Nisqually River, W of Longmire, 774 m, 46.73469°N, 121.83055°W	CAS
*Nebria piute*	DHK1098	USA, Utah, Beaver County, North Fork Three Creeks, 3044 m, 38.32708°N, 112.37001°W	CAS
*Nebria praedicta*	SDS08-423b	USA, California, Trinity County, Thompson Peak, upper Grizzly Lake Basin, 2411 m, 41.00458°N, 123.04799°W	CAS
*Nebria prezwalskii*	DHK1950	China, Qinghai, S of Mt. Maqen Gangri, 4360 m, 34.55028°N, 98.14667°E	CAS
*Nebria psammodes*	DHK1908	France, Vaucluse, Toulourenc River at Brantes, 473 m, 44.19050°N, 5.33387°E	CAS
*Nebria pseudorestias*	DHK2081	Nepal, Taplejung distr., S of Kangchenjunga Himal, Timbu(wa) Pokhari, 4350–4500 m, 27.43°N, 88.05°E	CAS
*Nebria purpurata* CO	DHK0843	USA, Colorado, Summit County, Quandary Peak, 3763 m, 39.39159°N, 106.11371°W	CAS
*Nebria purpurata* NM	DHK1034	USA, New Mexico, Taos County, Red River at Junebug Campground, 2620 m, 36.70716°N, 105.43314°W	CAS
*Nebria quileute*	DHK0456a	USA, Washington, Olympic National Park, Boulder Creek, 47.97607°N, 123.69291°W	CAS
*Nebria rathvoni*	DHK0734	USA, California, Tuolumne County, Deadman Creek, 2667 m, 38.31763°N, 119.66534°W	CAS
*Nebria retingensis*	DHK2070	China, Xizang, Gangdise Shan, side valley S of Reting, 4500–4800 m, 30.26583°N, 91.53444°E	CAS
*Nebria riversi*	SDS08-377G	USA, California, Mono County, lake due S of Donohue Pass, 3377 m, 37.75289°N, 119.24847°W	CAS
*Nebria roborowskii*	DHK1955	China, Qinghai, pass Bayankala, 4700–4800 m, 34.12694°N, 97.65722°E	CAS
*Nebria roddi*	SDS13-086A	Russia, Altai Republic, north slope Krasnaya Mt., 2013 m, 50.07248°N, 085.22120°E	CAS
*Nebria rubripes*	DHK1871	France, Cantal, Le Lioran, 1212 m, 45.07996°N, 2.74542°E	CAS
*Nebria rubrofemorata*	DHK1464	Russia, Altai Krai, Altai Mts., watershed of Belogolosov Korgon and Inya Rivers, 2000–2250 m, 50.95°N, 83.645°E	CAS
*Nebria saeviens*	DHK1376	Japan, Honshu, Niigata Prefecture, Road 403 NE of Nozawaonsen, 841 m	CAS
*Nebria sahlbergii*	DHK0968	USA, Washington, Mt. Rainier National Park, Nisqually River, W of Longmire, 774 m, 46.73469°N, 121.83055°W	CAS
*Nebria sajanica*	DHK0401b	Russia, Buryat Republic, Tunka Mts., Kingarga River, 1852 m, 51.98045°N, 102.37566°E	CAS
*Nebria sajanica* gr sp 1	DHK1452	Russia, Republic of Tuva, Western Sayan Mts., near Kozhagar Mt., 2250–2450 m, 51.54°N, 89.17°E	CAS
*Nebria sajanica* gr sp 1	DHK1453	Russia, Republic of Tuva, Western Sayan Mts., near Kozhagar Mt., 2250–2450 m, 51.54°N, 89.17°E	CAS
*Nebria sajanica* gr sp 2	DHK1783	Russia, Krasnoyarsky Krai, Western Sayan Mts., Oiskiji Pass, 1385 m, 52.87277°N, 93.25745°E	CAS
*Nebria sarlyk*	DHK1735	Russia, Altai Republic, Sarlyk Mt., 2040 m, 51.061050°N, 85.690640°E	CAS
*Nebria sayana*	SDS13-205A	Russia, Republic of Khakassia, Samblyl Mt., headwaters of Karasibo River, 1890 m, 52.15164°N, 090.18011°E	CAS
*Nebria schwarzi*	DHK0427	Canada, Alberta, Cline River at Highway 11, 1325 m, 52.16983°N, 116.48304°W	CAS
*Nebria sevieri*	DHK1111	USA, Utah, Iron County, Parowan Creek, 12.5 miles S of Parowan, 2785 m, 37.71458°N, 112.84760°W	CAS
*Nebria shiretokoana*	SDS12-263A	Japan, Hokkaido, Shiretoko Peninsula, Rausu-daira, 1234 m, 44.07454°N, 145.13095°E	CAS
*Nebria sierrablancae*	DHK0431a	USA, New Mexico, Lincoln County, Sierra Blanca Ski Area, 3000 m, 33.39998°N, 105.78932°W	CAS
*Nebria sierrae*	DHK0052	USA, California, Mono County, below Conness Lakes, 3134 m, 37.97600°N, 119.29690°W	CAS
*Nebria siskiyouensis*	DHK0423a	USA, California, Trinity County, Big Flat Campground, 1490 m, 41.06465°N, 122.93493°W	CAS
*Nebria sitnikovi*	SDS12-131A	Russia, Altai Republic, Bashchelakskii Mts., 2012 m, 51.24219°N, 84.20432°E	CAS
*Nebria snowi*	DHK0460a	Russia, Kuril Islands, Ushishir Archipelago, Yankicha Island, inland from Kraternaya Bay, 20 m, 47.50867°N, 152.81950°E	CAS
*Nebria sonorae*	DHK0785	USA, California, Tuolumne County, Blue Canyon Creek, 2822 m, 38.31068°N, 119.66147°W	CAS
*Nebria* sp GAN	DHK2068	China Gansu, Lianhuashan, Shaltan, 2850 m	CAS
*Nebria* sp MG 1	DHK1138	Mongolia, Bayankhongor Province, northern slope of Ikh Bogd Uul, 1730 m, 44.979°N, 100.638°E	CAS
*Nebria* sp MG 2	DHK1139a	Mongolia, Ömnögovi Province, Gobi Gurvanshaikhan National Park, Yolyn Am, 2285 m, 43.496°N, 104.089°E	CAS
*Nebria* sp XIZ 14	DHK0684	China, Xizang, Mainling, Paiqu, W of Doxong Pass, 4060 m, 29.48978°N, 094.93735°E	CAS
*Nebria* sp XIZ 16	DHK0679	China, Xizang, Bomi Zhamo, Garlungla, 3975 m, 29.76340°N, 95.70164°E	CAS
*Nebria* sp XIZ 17	DHK0681	China, Xizang, Bomi Zhamo, Garlungla, 3975 m, 29.76340°N, 95.70164°E	CAS
*Nebria* sp XIZ 20	DHK2008	China, Xizang, Transhimalaya, south slope Lungmari Mts., 4900–5300 m, 30.45°N, 86.50°E	CAS
*Nebria* sp XIZ 5	DHK0682	China, Xizang, Medog, Baibung, E of Doxong Pass, 4074 m, 29.49009°N, 94.95566°E	CAS
*Nebria* sp YUN 1	DHK0013	China, Yunnan, Tengchong County, Wuhe Township, Xiaodifang village, 2150 m, 24.43300°N, 098.75000°E	CAS
*Nebria* sp YUN 4	DHK0489a	China, Yunnan, Gongshan County, Bingzhongluo Township, 0.8 km N of Chukuai Lake, 3950 m, 27.98817°N, 098.47436°E	CAS
*Nebria* sp YUN 6	DHK0003	China, Yunnan, Longling Couty, Zhen’an Township, Bangbie village, 1540 m, 24.81306°N, 098.81667°E	CAS
*Nebria* sp YUN 9	DHK0087	China, Yunnan, Gongshan County, Cikai, Nu Jiang at Dashaba, 1443 m, 27.73606°N, 98.67113°E	CAS
*Nebria* sp YUN 10-dark	DHK0024	China, Yunnan, Tengchong County, Jietou Township, Yongangiao, 1500 m, 25.32556°N, 98.60000°E	CAS
*Nebria* sp YUN 10-pale	DHK0366b	China, Yunnan, Tengchong County, 2 km E of Qushi, 25.23944°N, 098.61667°E	CAS
*Nebria* sp YUN 11	DHK0086	China, Yunnan, Gongshan County, 0.6 km N of Dizhengdang, Dulong, 1898 m, 28.08680°N, 98.32835°E	CAS
*Nebria* sp YUN 13	DHK0483a	China, Yunnan, Gongshan County, Cikai Township, 0.1 km SE of Heipu Yakou, 3720 m, 27.76978°N, 098.44681°E	CAS
*Nebria* sp YUN 18	DHK0651	China, Yunnan, Lijiang County, Laojunshan, 3659 m, 26.64237°N, 99.74392°E	CAS
*Nebria spatulata*	SDS06-227B	USA, California, Fresno County, Sphinx Lakes, upper lake below Sphinx Crest, 3385 m, 36.71427°N, 118.51487°W	CAS
*Nebria splendida*	DHK2085	China, Xinjiang, W of Boro-Horo Mts., Mynmaral Mts., 1880 m, 44.10083°N, 82.19889°E	CAS
*Nebria steensensis*	DHK0042	USA, Oregon, Harney County, Steens Mts., Little Blitzen River, 2663 m, 42.69593°N, 118.59060°W	CAS
*Nebria subdilatata*	DHK0012	Russia, Buryat Republic, Khamar-Daban Mts., Tankhoy, 503 m, 51.53636°N, 105.10694°E	CAS
Nebria cf. superna	DHK1979	China, Xizang, Transhimalaya, pass Gyatso La S of Lhatse, 5200–5300 m, 28.95278°N, 87.43750°E	CAS
*Nebria suturalis* AB	DHK0435	Canada, Alberta, toe of Athabasca Glacier, 2047 m, 52.20819°N, 117.23597°W	CAS
*Nebria suturalis* CO	DHK0837	USA, Colorado, Clear Creek County, Mt. Evans, 4312 m, 39.58790°N, 105.64229°W	CAS
*Nebria suturalis* NH	DHK1151	USA, New Hampshire, Coos County, Mt. Washington, 1886 m, 44.26909°N, 71.30353°W	CAS
*Nebria sylvatica*	DHK0415a	USA, Washington, Olympic National Park, Boulder Creek, 655 m, 47.97607°N, 123.69291°W	CAS
*Nebria tekesensis*	DHK2084	China, Xinjiang, Narat Mts., W of Sarytur Pass, 3280 m, 42.90417°N, 82.62139°E	CAS
*Nebria teletskiana*	SDS12-153C	Russia, Altai Republic, Simultinskii Mts., Yambash Mt., 2006 m, 51.36033°N, 087.13060°E	CAS
*Nebria tibialis*	DHK1395	Italy, Tuscany, Vallombrosa, 43.733°N, 11.557°E	CAS
*Nebria triad*	DHK0471a	USA, California, Trinity County, South Fork Salmon River at Big Flat Campground, 1500 m, 41.06465°N, 122.93493°W	CAS
*Nebria trifaria*	DHK0434a	USA, Utah, Utah County, South Fork American Fork River, 2207 m, 40.43519°N, 111.63603°W	CAS
*Nebria turcica*	DHK1947	Turkey, Erzincan, Sipikor Gecidi, 2433 m, 39.88472°N, 39.56507°E	CAS
*Nebria turmaduodecima*	SDS08-424a	USA, California, Trinity County, Thompson Peak, upper Grizzly Lake Basin, 2411 m, 41.00458°N, 123.04799°W	CAS
*Nebria uenoiana*	DHK1588	Taiwan, Chiayi County, AlishanTownship, Laonong River, 3020 m, 23.40131°N, 121.93831°E	CAS
*Nebria utahensis*	DHK1075	USA, Utah, Garfield County, Henry Mountains, Lonesome Beaver, 2510 m, 38.10945°N, 110.77838°W	CAS
*Nebria vandykei*	DHK0932	USA, Washington, Mt. Rainier National Park, Paradise area, Edith Creek Basin, 1807 m, 46.79678°N, 121.72711°W	CAS
*Nebria wallowae*	DHK0980	USA, Oregon, Wallowa County, West Fork Wallowa River below Glacier Lake, 2479 m, 45.16528°N, 117.28163°W	CAS
*Nebria walteriana*	DHK1959	China, Xizang, NE of pass Shogu La, 5000–5350 m, 29.91333°N, 90.14111°E to 29.95556°N, 90.13028°E	CAS
*Nebria wyeast*	DHK0418b	USA, Oregon, Clackamas County, Mt. Hood, Timberline Lodge, 1876 m, 45.33539°N, 121.70849°W	CAS
*Nebria yuae*	DHK0603	China, Sichuan, Luding County, Gongga Shan, 3753 m, 29.58493°N, 102.02437°E	CAS
*Nebria yunnana*	DHK0377a	China, Yunnan, Zhanyi Co., Huashan Reservoir, 2000 m, 25.75°N, 103.61°E	CAS
*Nebria zioni*	DHK1108	USA, Utah, Iron County, Parowan Creek, 12.5 miles S of Parowan, 2785 m, 37.71458°N, 112.84760°W	CAS
*Nippononebria altisierrae*	DHK1547	USA, California, Alpine County, Carson Pass, 2616 m, 38.69377°N, 119.98749°W	CAS
*Nippononebria campbelli*	SDS13-388A	USA, Washington, Mt. Rainier National Park, Edith Creek Falls, 1613 m, 46.79051°N, 121.73016°W	CAS
*Nippononebria chalceola*	SDS14-629	Japan, Honshu, Fukui Prefecture, Ikeda Town, Mt. Heko, 1300 m	CAS
*Nippononebria changbaiensis*	SDS12-199a	China, Jilin, northern slope of Changbai Shan, 1901 m, 42.03954°N, 128.05562°E	CAS
*Nippononebria virescens*	DHK0348	USA, Oregon, Benton County, 0.3–0.5 miles W of Alsea Falls, 346 m, 44.32065°N, 123.50012°W	CAS
*Notiophilus borealis*	DHK0273b	USA, Alaska, Denali National Park, Wonder Lake, 731 m, 63.46222°N, 150.83167°W	CAS
*Notiophilus semistriatus*	DHK1335	USA, Idaho, Shoshone County, near Hobo Cedar Grove, 1425 m, 47.08477°N, 116.11538°W	CAS
*Notiophilus sierranus*	DHK1490	USA, California, El Dorado County, Blodgett Experimental Forest, 1300 m, 38.90958°N, 120.66123°W	CAS
*Notiophilus sylvaticus*	DHK1292	Canada, British Columbia, Haida Gwaii, Graham Island, Chinukundl Creek, 7 m, 53.32412°N, 131.95519°W	CAS
*Opisthius richardsoni*	DHK0885	USA, Washington, Whatcom County, Nooksack River at Cedarville, 45 m, 48.84072°N, 122.29334°W	CAS
*Paropisthius chinensis*	DHK0612	China, Sichuan, Luding County, Gongga Shan, 3045 m, 29.57393°N, 101.99204°E	CAS
*Paropisthius davidis*	DHK0645	China, Yunnan, Lijiang County, Laojunshan, 3502 m, 26.64210°N, 99.76745°E	CAS
*Paropisthius indicus*	DHK0329a	China, Yunnan, Cikae Township, Dabadi,3022 m, 27.79655°N, 98.50562°E	CAS
*Paropisthius masuzoi*	DHK1583	Taiwan, Nantou County, Ren’ai Township, Hehuanshan, 3090 m, 24.14367°N, 121.28075°E	CAS
*Pelophila borealis* AK	DHK0334b	USA, Alaska, Kodiak Island, Buskin River, 20 m, 57.76541°N, 152.52016°W	CAS
*Pelophila borealis* RFE	DHK2064	Russia, Kamtschatka, os. Asabatsch’e and river Kamtschatka, 56.22278°N, 162.01917°E	CAS
*Pelophila rudis*	DRM2376	USA, Alaska, Fairbanks, east shore of Smith Lake, 150 m, 64.8622°N, 147.8616°W	OSAC
outgroup specimens			
*Bembidion antiquum*	DRM1963	USA, Missouri, Carter County, Current River at Van Buren, 135 m, 36.99037°N, 91.01672°W	OSAC
*Calosoma scrutator*	DHK1712	USA, Oklahoma, Sequoyah County, Sallisaw, 245 m, 35.53371°N, 94.62467°W	CAS
*Calosoma scrutator*	DRM2249	USA, Arizona, Santa Cruz County, Pena Blanca, 31.3852°N, 111.0931°W	OSAC
*Loricera pilicornis*	DHK1185	Canada, Newfoundland, Deer Lake, north shore, 5 m, 49.18058°N, 57.45150°W	CAS
*Metrius contractus*	DHK1424	USA, California, Mendocino County, Angelo Reserve, Skunk Creek, 420 m, 39.72554°N, 123.64412°W	CAS
*Pterostichus melanarius*	DRM2311	USA, Wisconsin, Dane County, Madison, 43.086°N, 89.425°W	OSAC
*Scaphinotus petersi*	DRM0878	USA, Arizona, Pima County, Mt. Lemmon Ski Valley, 2500 m, 32.447°N, 110.777°W	OSAC
*Trachypachus inermis*	DHK0738a	USA, California, Tuolumne County, Sonora Pass, 2920 m, 38.32961°N, 119.63857°W	CAS
*Trachypachus slevini*	DHK0513a	USA, Oregon, Lincoln County, Moolack Beach, 8 m, 44.71078°N, 124.06035°W	CAS

## Appendix B. PCR protocols and primers

We obtained sequence data for more than 97% of those possible for the eight gene fragments for our taxon sample using the protocols and primers listed below.

**Table:** PCR thermal cycling conditions for recommended protocols. The Pattern column indicates cycling pattern. “S”, for standard reaction pattern, included a starting phase of 2 or 3 minutes at 94 °C, followed by the cycling phase, with each cycle consisting of 20–30 seconds of denaturing at 94 °C, 20–30 seconds of annealing at the annealing temperature, and an extension phase at the temperature specified by the Taq manufacturer. “T”, for touchdown reaction, is like the standard reaction but with three cycling phases, each with a separate annealing temperature. The Cycles column lists the usual number of cycles, with all variant numbers used successfully to generate at least one sequence given in parentheses. For touchdown reactions, the numbers are given for each cycling phase, separated by commas. The Ta column lists the usual annealing temperature in °C (with less used but successful temperatures in parentheses). For touchdown reactions, the annealing temperatures are given for each cycling phase, separated by commas. The Ext column lists the duration of the extension phase in seconds, with less used but successful durations in parentheses. The Gene column lists the gene(s) successfully amplified with each set of conditions. Subscripts “ex” and “in” refer to conditions used for outer and inner nested (or hemi-nested reactions), respectively.

#	Pattern	Cycles	Ta	Ext	Gene
C1	S	35 (36)	52 (45, 50)	70	28S, 16S-ND1, COIBC, COIPJ
C2	S	37	52	120	*wg*_ex_
C3	S	35 (37)	54	150	*wg*_in_
C4	T	6 (5), 6(5), 36 (35)	57, 52, 45	90 (60, 120, 180, 300, 330)	CAD, Topo
C5	T	6, 6, 36	60, 55, 50	120 (60, 90, 210, 300)	PEPCK_ex_
C6	S	35	60	90 (300)	PEPCK_in_

**Table:** PCR primers used for both amplification and sequencing. The Dir column identifies forward (F) and reverse (R) primers and indicates external (ex) and internal (in) primers used for nested or hemi-nested PCR. The Ref column lists the reference for the original description of the primer: (1) Maddison 2008; (2) Moore and Robertson 2014; (3) Moulton and Wiegmann 2004; (4) Ober 2002; (5) Simon et al. 1994; (6) Van der Auwera et al. 1994; (7) Vogler and DeSalle 1993; (8) Ward and Downie 2005; (9) Wild and Maddison 2008.

Gene	Primer	Dir	Sequence	Ref
28S	LS58	F	GGGAGGAAAAGAAACTAAC	4
	NLF184/21	F	ACCCGCTGAAYTTAAGCATAT	6
	LS998	R	GCATAGTTCACCATCTTTC	4
	LS1041	R	TACGGACRTCCATCAGGGTTTCCCCTGACTTC	1
16S-ND1	16SAR	F	CGCCTGTTTAACAAAAACAT	7
	ND1A	R	GGTCCCTTACGAATTTGAATATATCCT	5
CO1 PJ	Jer	F	CAACATTTATTTTGATTTTTTGG	5
	Pat	R	TCCAATGCACTAATCTGCCATATTA	5
CO1 BC	LCO1490	F	GGTCAACAAATCATAAAGATATTGG	1
	HCO2198	R	TAAACTTCAGGGTGACCAAAAAATCA	1
*wg*	*wg*550F	F_ex_	ATGCGTCAGGARTGYAARTGYCAYGGYATGTC	9
	*wg*578F	F_in_	TGCACNGTGAARACYTGCTGGATG	8
	*wg*AbR	R_in_	YTCGCAGCACCARTGGAA	9
	*wg*AbRZ	R_ex_	CACTTNACYTCRCARCACCARTG	8
CAD2	CD398F	F	GARCAYACAGCNGGNCCNCAAGA	3
	CD581F4	F	GGWGGWCAAACTGCWYTMAAYTGYGG	1
	CD696R	R	AANGGRTCNACRTTTTCCATATT	2
PEPCK	PK282	F_ex_	GAAGGATGGCTBGCNGARCAYATG	9
	PK330F	F_in_	GAGGACCAAGCNGAYACNGTDGGTTGTTG	9
	PK485R	R	GCAGCVGTNGCYTCRCTYCTCAT	9
Topo	TP643F	F	GACGATTGGAARTCNAARGARATG	9
	TP675F	F	GAGGACCAAGCNGAYACNGTDGGTTGTTG	9
	TP932R	R	GGWCCDGCATCDATDGCCCA	9

## Supplementary materials

Supplementary material 1 Tables S1–S3 Data type clades data Explanation note **Table S1.** Unique bases supporting clades. **Table S2.** Unique amino acids supporting clades. **Table S3.** Unique insertions and deletions supporting clades. https://binary.pensoft.net/file/553668This dataset is made available under the Open Database License (http://opendatacommons.org/licenses/odbl/1.0/). The Open Database License (ODbL) is a license agreement intended to allow users to freely share, modify, and use this Dataset while maintaining this same freedom for others, provided that the original source and author(s) are credited.David H. Kavanaugh, David R. Maddison, W. Brian Simison, Sean D. Schoville, Joachim Schmidt, Arnaud Faille, Wendy Moore, James M. Pflug, Sophie L. Archambeault, Tinya Hoang, Jei-Ying Chen

Supplementary material 2 Figures S1–S13 Data type phylogenetic data Explanation note **Figure S1.** Majority rule consensus tree of 150,000 trees from Bayesian analysis of eight-gene concatenated matrix. **Figure S2.** Maximum likelihood trees for concatenated matrix of all nuclear genes. **Figure S3.** Maximum likelihood trees for concatenated matrix of all nuclear protein-coding genes. **Figure S4.** Maximum likelihood trees for concatenated matrix of all mitochondrial genes. **Figure S5.** Maximum likelihood trees for 28S. **Figure S6.** Maximum likelihood trees for 16S-ND1. **Figure S7.** Maximum likelihood trees for the COIBC. **Figure S8.** Maximum likelihood trees for the COIPJ. **Figure S9.** Maximum likelihood trees for the CAD2. **Figure S10.** Maximum likelihood trees for the PEPCK. **Figure S11.** Maximum likelihood trees for the Topo. **Figure S12.** Maximum likelihood trees for the *wg.***Figure S13.** Maximum likelihood tree for concatenated matrix showing only those taxa for which PEPCK was sequenced, with branches colored according to the squared-change parsimony reconstruction of the length of the PEPCK intron. https://binary.pensoft.net/file/553669This dataset is made available under the Open Database License (http://opendatacommons.org/licenses/odbl/1.0/). The Open Database License (ODbL) is a license agreement intended to allow users to freely share, modify, and use this Dataset while maintaining this same freedom for others, provided that the original source and author(s) are credited.David H. Kavanaugh, David R. Maddison, W. Brian Simison, Sean D. Schoville, Joachim Schmidt, Arnaud Faille, Wendy Moore, James M. Pflug, Sophie L. Archambeault, Tinya Hoang, Jei-Ying Chen
