# Supplementary material for: Phylogeny of the supertribe Nebriitae (Coleoptera, Carabidae) based on analyses of DNA sequence data
Source: Zookeys. 2021 Jun 16;1044:41–152. doi: 10.3897/zookeys.1044.62245 (PMC8222211; doi:10.3897/zookeys.1044.62245)
Supplement: Supplementary material 1 — Tables S1–S3 [file zookeys-1044-041-s001.docx]

## Supplementary Tables

**Table S1.** Unique bases supporting clades. Columns for each gene fragment provide the position number in the eight-gene concatenated matrix and the letter code (in parentheses) for each base unique to a clade as shown in Fig. 4. The range of position numbers for each gene fragment in the concatenated matrix is provided (in parentheses) in the column headings. “#” is the total number of unique bases supporting each clade. Bases in bold type are unique within the entire taxon sample including outgroups, those in bold italics are unique among nebriites in the taxon sample.

| **clade** | **28S (1-889)** | **16S-ND1 [16S] (890-1514)** | **16S-ND1 [tRNA-Leu] (1515-1574)** | **16S-ND1 [ND1] (1576-1679)** | **CO1 PJ (1680-2498)** | **CO1 BC (2499-3156)** | **CAD2 (3157-3961)** | **Topo (3962 -4716)** | **PEPCK (4717-5183)** | ***wg*  ( 5184-5654)** | **#** |
| --- | --- | --- | --- | --- | --- | --- | --- | --- | --- | --- | --- |
| **Pelophilini + Opisthiini** |  |  |  |  |  |  |  |  |  | **5337 (G)** | **1** |
| **Pelophilini** | **430 (A), 433 (G), 436 (A), 623 (C), 648 (G)** |  |  |  |  |  | **3195 (G), 3212 (C), 3235 (C), 3260 (C), 3281 (C), 3285 (C), 3508 (C), 3736 (G)** | **4016 (T), 4409 (C)** |  | **5413 (C), 5414 (A), 5561 (G)** | **18** |
| **Opisthiini** | **291 (C), 350 (C)** | **1507 (T)** |  |  |  |  | **3199 (C), 3643 (C)** | **4370 (T)** |  | **5375 (T)** | **7** |
| ***Paropisthius*** | **408 (C), 450 (C), 586 (C), 633 (A), 651 (T)** | **1141 (T), 1481 (G)** |  |  | **1984 (G), 2182 (T), 2304 (G)** |  | **3267 (G), 3299 (A), 3532 (C), 3535 (C), 3586 (G)** | **4186 (C)** | **5088 (T)** |  | **17** |
| ***Nippononebria* s. str.** |  |  |  |  |  | **2776 (A), 2815 (T)** |  |  |  |  | **2** |
| ***Vancouveria*** | **546 (A), 851 (C)** |  |  |  | **2173 (A), 2187 (A)** | **2981 (G)** |  | **4022 (C)** |  |  | **6** |
| ***Leistus*** |  |  |  |  | **1963 (T)** |  |  |  |  |  | **1** |
| ***Leistus* + *Evanoleistus*** | ***662 (T)*** |  |  |  |  |  |  |  |  |  | ***1*** |
| ***nitidus* to *longipennis* clade** | ***430 (T),* 677(C)** |  |  |  |  |  |  |  |  |  | **1 *1*** |
| ***nitidus* to *longipennis* clade** | ***430 (T)*** |  |  |  |  |  |  |  |  |  | ***1*** |
| ***Neoleistus*** | **412 (A)** |  |  | **1656 (T)** |  |  |  |  | **4913 (G)** |  | **3** |
| ***Nebria*** | **524 (T), 538 (A)** |  |  |  |  |  |  |  |  |  | **2** |
| ***Oreonebria* Series** | **339 (C)** |  |  |  |  |  |  |  |  |  | **1** |
| ***Eonebria* Complex** |  |  |  |  |  | **2578 (T)** |  |  |  |  | **1** |
| ***Parepinebriola*** | **647 (A)** | **1359 (A)** |  |  |  |  |  |  |  |  | **2** |
| ***Sadonebria* + *Eonebria*** | **683 (C)** |  |  |  |  | **2993 (C)** |  |  |  |  | **2** |
| ***Sadonebria*** | **150 (A), 154 (A), 397 (C), 432 (A), *441 (A),* 610 (T), 659 (C), *817 (G),* 821 (C), *824 (T), 881 (T),* 883 (A)** |  |  | **1586 (A)** |  |  | ***3679 (A)*** |  |  | ***5585 (C)*** | **9 *6*** |
| ***Eonebria*** | **659 (T)** |  |  |  |  |  |  |  |  |  | **1** |
| ***Epispadias* + *Falcinebria*** | **383 (C), 766 (A)** | **1357 (G)** |  | **1601 (G)** |  | **2599 (A)** |  | **4534 (T), 4609 (G)** |  |  | **7** |
| ***Epispadias* + *Falcinebria*** | ***449 (G)*** |  |  |  |  |  |  |  | ***5097(G)*** |  | ***2*** |
| ***Orientonebria* + *Archastes* + *Oreonebria*** | **444 (T), *576(T)*** |  |  |  |  |  |  |  |  |  | **1 *1*** |
| ***Orientonebria* + *Archastes*** |  | **1024 (G)** |  |  | **1951 (T), 2141 (A)** |  |  |  | ***4747 (C),*  4803 (C), 4967 (G)** | **5426 (A)** | **6 *1*** |
| ***Oreonebria* + *Nebriorites* + *Marggia*** | ***383 (T)*** |  |  |  |  |  |  |  |  |  | ***1*** |
| ***Nebriola* Series + *Nebria* Series + *Catonebria* Series** | **347 (C), 482 (G), *708 (T),* 711 (G)** |  |  |  |  |  |  |  |  |  | **3 *1*** |
| ***Nebriola*** | **571 (C), *660 (C)*** |  |  |  | **2205 (T), 2373 (G), 2375 (A)** | ***2505 (T),*  2950 (T)** | **3682 (T)** | ***4282 (A)*** |  |  | **6 *3*** |
| ***Nebria* Series** | **481 (T), 573 (G)** |  |  |  |  |  |  |  |  |  | **2** |
| ***Boreonebria* Complex** | **341 (A), 500 (A), 606 (A), 685 (T)** | **1252 (G)** |  |  |  | **3103 (A)** |  |  |  |  | **6** |
| ***Boreonebria*** |  | **1287 (T)** |  |  |  |  |  |  |  |  | **1** |
| ***hudsonica* group** | **360 (C)** |  |  |  |  |  |  |  |  |  | **1** |
| ***nivalis* group** |  |  |  |  |  |  | ***3656 (A)*** |  |  |  | ***1*** |
| ***N. intermedia* + *N. crassicornis*** |  |  |  |  |  |  |  | **4273 (G), 4681 (G)** |  |  | **2** |
| ***Nebria* Complex** | ***500 (C)*** |  |  |  |  |  |  |  |  |  | ***1*** |
| ***Nebria* Subcomplex** |  |  | **1565 (T)** |  |  |  |  |  |  |  | **1** |
| ***Tyrrhenia*** | ***236 (T),*  475 (G), 602 (C), 850 (-)** |  |  |  |  |  |  |  |  |  | **3**  ***1*** |
| ***Alpaeonebria* + *Spelaeonebria* + European *Nebria*** | **603 (-)** |  |  |  |  |  |  |  |  |  | **1** |
| ***Alpaeonebria*** | **602 (-), 604 (-)** |  |  |  |  |  |  |  |  |  | **2** |
| **European *Nebria* s. str.+ *Spelaeonebria*** | **602 (T)** | **1491 (C)** |  |  |  |  |  |  |  |  | **2** |
| ***Epinebriola* Subcomplex** | **341 (T)** |  |  |  |  |  |  |  |  |  | **1** |
| ***Epinebriola* + *Barbonebriola* + *Paranebria* + *Patrobonebria*** | ***360 (T), 710 (A)*** |  |  |  |  |  |  |  |  |  | **1 *1*** |
| ***N. businskyorum* + *N. cf. laevistriata*** | **666 (C)** | **1135 (T)** |  |  | **2349 (T)** |  |  |  |  |  | **3** |
| **remaining *Epinebriola* without *N. oxyptera*** | **238 (T)** |  |  |  | **1902 (A)** |  |  |  |  | **5406 (C), 5407 (A), 5408 (G), 5409 (C), 5410 (A),** | **7** |
| ***Patrobonebria*** | ***145 (A)*** |  |  |  |  |  |  |  |  |  | ***1*** |
| ***Asionebria* + *Psilonebria* + *przewalskii* group** | **471 (G), 634 (T), 821 (A), 882 (C)** |  |  |  | **2428 (T)** | ***2854 (T)*** |  | **4301 (C), 4412 (G)** | **4854 (G)** |  | **8 *1*** |
| ***N. mentoincisa* + *Psilonebria*** |  |  |  |  |  |  |  |  | ***5025 (A)*** |  | ***1*** |
| ***Psilonebria*** |  |  |  | **1597 (A)** |  |  |  |  |  |  | **1** |
| ***Eunebria* + *Eurynebria*** | **355 (G), *540 (G), 543 (A),* 789 (C), *792 (C)*** |  |  |  |  |  | **3278 (A)** |  |  |  | **3 *3*** |
| **Europe & Central Asian *Eunebria*** | **827 (T)** | **1194 (T)** |  |  |  |  |  |  |  |  | **2** |
| **European *Eunebria*** | **428 (G)** | **1241 (T)** |  |  |  |  |  |  |  |  | **2** |
| **Central Asian *Eunebria*** | ***149 (A),* 331 (G), *723 (G)*** |  |  |  |  |  |  |  |  |  | **2 *1*** |
| **Yunnan & E Asian *Eunebria*** | ***533 (A), 650 (A)*** | **1508 (A)** |  |  |  |  |  |  |  |  | **1 *2*** |
| **Far Eastern *Eunebria*** | ***813 (G)*** |  |  |  |  |  |  |  |  |  | ***1*** |
| ***Catonebria* Series** | **103 (G), 578 (A), 610 (C), 630 (T), 635 (T), 646 (T), 667 (C), 712 (C), 717 (C)** |  |  |  |  |  |  |  |  |  | **7 *2*** |
| ***Reductonebria* Complex** | **623 (T)** |  |  |  | **2413 (A)** |  |  | **4292 (A), 4508 (C)** |  |  | **4** |
| ***Insulanebria* (*snowi* group)** | **663 (G)** |  | **1529 (A)** |  |  |  |  |  |  |  | **2** |
| ***Reductonebria* + *Erwinebria*** |  |  | **1538 (A), 1550 (T)** |  |  |  |  |  |  |  | **2** |
| ***Catonebria* Complex** | ***357 (A)*** |  |  |  |  |  |  |  |  |  | ***1*** |
| ***Nivalonebria* (*paradisi* group)** | **237 (T), 620 (G)** |  |  |  |  |  | **3232 (C)** | ***4370 (G),* 4438 (T)** |  |  | **4 *1*** |
| ***Neaptenonebria* + *Palaptenonebria*** | **665 (T), *714 (T)*** |  |  |  |  |  |  | ***4436 (G)*** |  |  | **1 *2*** |
| ***Neaptenonebria*** |  |  |  |  |  |  |  | **4475 (A)** |  |  | **1** |
| ***Palaptenonebria*** |  |  |  |  |  |  | ***3958 (G)*** |  |  |  | ***1*** |
| ***Catonebria*** |  |  |  |  |  |  |  | **4060 (A)** |  |  | **1** |
| ***meanyi* subgroup** |  |  | **1535 (C)** |  |  |  |  |  |  |  | **1** |

**Table S2.** Unique amino acids supporting clades. Columns for each gene fragment provide the position (number) in the matrix for that fragment converted to a protein matrix and the name (in parentheses) for each amino acid unique to a clade as shown in Fig. 4. Amino acids in bold type are unique for the entire taxon sample; those in bold italics are unique among Nebriitae; those in regular type are unique among Nebriini; and those in regular italics are unique among *Nebria*. “#” is the number of amino acids unique at each level of inclusiveness supporting each clade; those preceded by a “+” indicate unique insertions; and those in brackets [ ] indicate unique deletions.

| **clade** | **16S-ND1**  **[ND1]** | **CO1 PJ** | **CO1 BC** | **CAD2** | **Topo** | **PEPCK** | ***wg*** | **#** |
| --- | --- | --- | --- | --- | --- | --- | --- | --- |
| **Nebriitae** |  |  |  |  |  |  | **27 (Valine), 35 (Valine), 46 (Valine),** | **3** |
| ***Notiophilus*** | **12 (Methionine)** | **238 (Methionine), 252 (Threonine), 253 (Valine)** | **99 (Threonine)** | **13 (Threonine), 40 (Isoleucine), 161 (Asparagine), 197 (Asparagine), 200 (Aspartic acid)** |  | ***94 (Isoleucine), 117 (Glutamine)*** | **41 (Glutamic acid), 44 (Threonine), 57 (Glycine), 58 (Asparagine), 60 (Valine), 62 (Serine), 63 (Lysine), 64 (Valine), 68 (Threonine),** | **19 *2*** |
| **Pelophilini + Opisthiini + Nebriini** |  | ***252 (Serine), 253 (Isoleucine)*** |  |  |  |  | **29 (Glycine)** | **1 *2*** |
| **Pelophilini+Opisthiini** |  |  |  | ***8 (Valine)*** | ***12 (Alanine)*** |  | **52 (Alanine), *126 (Glutamic acid)*** | **1 *3*** |
| **Pelophilini** |  | ***151 (Leucine)*** |  | **14 (Arginine), *15 (Arginine),*  20 (Histidine), 44 (Alanine)** | **20 (Leucine), *107 (Glutamic acid),* 151 (Glutamine)** |  | ***63 (Glycine),*  77 (Proline)** | **6 *4*** |
| ***Pelophila borealis*** |  |  |  | ***30 (Arginine),* 36 (Leucine)** | ***181 (Leucine), 191 (Lysine),*  192 (Serine)** |  |  | **2 *3*** |
| **Opisthiini** |  |  |  | ***15 (Asparagine),* 44 (Glutamine)** |  |  | ***64 (Threonine)*** | **1 *2*** |
| ***Opisthius*** |  |  |  |  | ***156 (Threonine),* 170 Glutamic acid)** |  | **68 (Glycine), 95 (Isoleucine)** | **3 *1*** |
| ***Paropisthius*** |  | **168 (Isoleucine), 209 (Valine)** |  | **14 (Glutamine), 38 (Arginine), 49 (Isoleucine)** |  |  |  | **5** |
| **Nebriini** |  |  |  |  |  |  | **11 (Histidine), *52 (Threonine),*** | **1 *1*** |
| ***Nippononebria* + *Leistus*** |  |  |  | 26 (Glutamine) |  |  |  | 1 |
| ***Nippononebria*** |  |  |  | **17 (Lysine), 143 (Glutamic acid)** |  |  |  | **2** |
| ***Nippononebria* s. str.** |  | **209 (Methionine)** | **94 (Isoleucine), 107 (Serine)** |  |  |  |  | **3** |
| ***Vancouveria*** |  | 158 (Tyrosine), **165 (Asparagine), 170 (Methionine)** |  | 39 (Isoleucine),  **44 (Histidine)** | **22 (Histidine)** |  | 25 (Serine), **144 (Glutamine)** | **5** 3 |
| ***Leistus*** |  | **95 (Phenylalanine)** |  |  |  |  |  | **1** |
| ***Sardoleistus* + *Pogonophorus* + *Leistus* + *Evanoleistus*** |  |  |  |  |  |  | **144 (Methionine)** | **1** |
| ***Pogonophorus*** | **23 (Isoleucine)** |  |  | **171 (Asparagine)** |  |  |  | **3** |
| ***Leistus* s. str.** |  |  |  |  | **[60 (Proline)]** |  |  | **[1]** |
| ***niger* clade + *Neoleistus*** |  |  |  | **230 (Alanaine)** |  | **87 (Aspartic acid)** |  | **2** |
| ***Neoleistus*** |  |  |  |  |  | **113 (Glycine)** |  | **1** |
| ***Oreonebria* Series** |  |  |  | *144 (Arginine)* |  |  |  | *1* |
| ***Parepinebriola*** |  |  |  |  |  | *198 (Threonine)* |  | *1* |
| ***Sadonebria* + *Eonebria*** |  |  | **166 (Threonine)** |  |  |  |  | **1** |
| ***Sadonebria*** | ***5 (Lycine)*** |  |  |  |  |  |  | ***1*** |
| ***Epispadias* + *Falcinebria*** | **10 (Cysteine)** | 256 (Tyrosine), *258 (Alanine),* **259 (Cysteine)** | **35 (Asparagine)** |  | **138 (Threonine)** |  |  | **4** 1 *1* |
| ***Falcinebria*** |  |  | **159 (Serine)** | **21 (Glutamine),** *22 (Valine)* | **235 (Asparagine)** |  |  | **3** *1* |
| ***Orientonebria* + *Archastes*** |  | **91 (Valine)** |  |  |  | ***58 (Histidine)*** | **81 (Leucine)** | **2 *1*** |
| ***Nebriola*** |  | **176 (Phenylalanine), 232 (Valine)** | **152 (Serine)** |  | ***108 (Glutamic acid)*** |  |  | **3 *1*** |
| ***Boreonebria* Complex** |  |  | **203 (Threonine)** |  |  |  |  | **1** |
| ***Nakanebria*** |  | **172 (Threonine), 271 (Isoleucine)** |  |  |  |  | **64 (Glycine)** | **3** |
| **European *Nebria* str. + *Spelaeonebria*** |  |  |  |  | ***12 (Threonine)*** |  |  | ***1*** |
| ***Epinebriola* Subcomplex** |  | *223 (Leucine)* |  |  |  |  |  | *1* |
| ***businskyorum* + *cf. laevistriata*** |  | **224 (Phenylalanine)** |  |  |  |  |  | **1** |
| ***martensi* group** |  | **75 (Isoleucine)** | **14 (Leucine)** |  |  |  |  | **2** |
| ***Paranebria*** |  | 259 (Phenylalanine) |  |  |  |  |  | 1 |
| ***Asionebria* + *Psilonebria* + *przewalskii* group** |  |  |  |  | **115 (Leucine), 152 (Glutamic acid)** |  |  | **2** |
| ***Psilonebria*** | **9 (Isoleucine)** |  |  | *152 (Lysine)* |  |  |  | **1** *1* |
| ***Eunebria* + *Eurynebria*** |  |  |  | **42 (Asparagine)** |  |  |  | **1** |
| **Yunnan & E Asian *Eunebria*** |  | *210 (Threonine)* |  |  |  |  |  | *1* |
| **Yunnan *Eunebria*** | **7 (Asparagine)** |  |  |  |  |  |  | **1** |
| ***Catonebria* Series** |  | 230 (Methionine) |  |  | *180 (Isoleucine)* |  |  | 1 *1* |
| ***Reductonebria* Complex** |  | *227 (Isoleucine),*  **245 (Tyrosine)** |  |  | **112 (Threonine)** |  |  | **2** *1* |
| ***Reductonebria*** |  | **214 (Leucine)** |  |  |  |  |  | **1** |
| ***Erwinebria*** |  | 172 (Glutamine) |  |  |  |  |  | 1 |
| ***Nivalonebria*** |  |  |  |  | ***138 (Glycine),*  160 (Isoleucine)** |  |  | **1 *1*** |
| ***Neaptenonebria* + *Palaptenonebria*** |  |  |  |  | ***160 (Valine)*** |  |  | ***1*** |
| ***Neaptenonebria*** |  |  |  |  | **173 (Asparagine)** |  |  | **1** |
| ***Palaptenonebria*** |  |  |  |  |  |  | *32 (Asparigine)* | *1* |
| ***gebleri* group** |  | ***233 (Valine)*** |  | *15 (Lysine)* |  |  |  | ***1*** *1* |
|  |  |  |  |  |  |  |  |  |

**Table S3.** Unique insertions and deletions supporting clades. The column for 28S provides the position number(s) in the 28S matrix and the number of bases involved in the unique insertion or deletion. The column for *wg* provides the position number(s) for each unique insertion or deletion in the matrix for that fragment converted to a protein matrix and the name (in parentheses) of the amino acid(s) inserted or the number of amino acids deleted for clades as shown in Fig. 4. Entries in bold type are unique for the entire taxon sample; those in bold italics are unique among Nebriitae; and those in regular type are unique among *Nebria.*

| **clade** | **28S** | ***wg*** |
| --- | --- | --- |
| ***Notiophilus*** | **907-908 (two-base deletion), 1113 (single-base insertion), *1262 (single-base insertion)*** | **40 (Arginine insertion), 47-49 (three-AA deletion), 54 (single-AA deletion), 77 (single-AA deletion)** |
| **Pelophilini + Opisthiini + Nebriini** |  | **38 (Glutamine insertion)** |
| ***Opisthius*** | **1247-1249 (three-base insertion)** |  |
| ***Paropisthius*** | **1233-1235 (three-base insertion)** |  |
| ***Leistus*** | **1127 (single-base deletion), 1134 (single-base deletion), 1202 (single-base deletion),** |  |
| ***Leistus + Evanoleistus*** | **1126 (single-base deletion), 1207 (single-base deletion)** |  |
| ***Sadonebria*** | **83 (single-base insertion), *408 (single-base insertion),* 454-486 (33-base insertion), 1373-1384 (11-insertion),** 1484-1495 (12-base insertion) |  |
| ***Eonebria*** | ***792-793 (two-base insertion),* 1251 (single-base deletion)** |  |
| **Central Asian *Eonebria*** | **448-449 (two-base insertion, 1093 (single-base insertion)** |  |
| ***Falcinebria*** | **685 (single-base insertion)** |  |
| ***Nebriola* Series + *Nebria* Series + *Catonebria* Series** | **365 (single-base insertion), 695 (single-base insertion)** |  |
| ***Nebriola*** | **893 (single-base insertion), *982 (single-base deletion),*  1355 (single-base insertion), 1524 (single-base deletion)** |  |
| ***Tyrrhenia*** | **909-911 (three-base insertion), 916-917 (two-base inseretion), 1524 (single-base deletion), 1528 (single-base deletion)** |  |
| ***Alpaeonebria* + *Spelaeonebria* + *Nebria*** | ***1525-1527 (three-base insertion)*** |  |
| ***Alpaeonebria*** | **1097 (single-base deletion), 1100 (single-base deletion)** |  |
| ***businskyorum* + *cf. laevistriata*** | **1088-1089 (two-base insertion)** |  |
| ***martensi* group** |  | **74-76 (three Glutamine insertions)** |
| ***Eunebria* + *Eurynebria*** | **797 (single-base insertion)** |  |
| ***Catonebria* Series** | 1203-1204 (two-base deletion) |  |
| ***Reductonebria*** | **1136 (single-base deletion)** |  |
| ***Neaptenonebria* + *Palaptenonebria*** | **799 (single-base insertion)** |  |
