# Supplementary material for: Phylogeny of the supertribe Nebriitae (Coleoptera, Carabidae) based on analyses of DNA sequence data
Source: Zookeys. 2021 Jun 16;1044:41–152. doi: 10.3897/zookeys.1044.62245 (PMC8222211; doi:10.3897/zookeys.1044.62245)
Supplement: Supplementary material 2 — Figures S1–S13 [file zookeys-1044-041-s002.pdf]

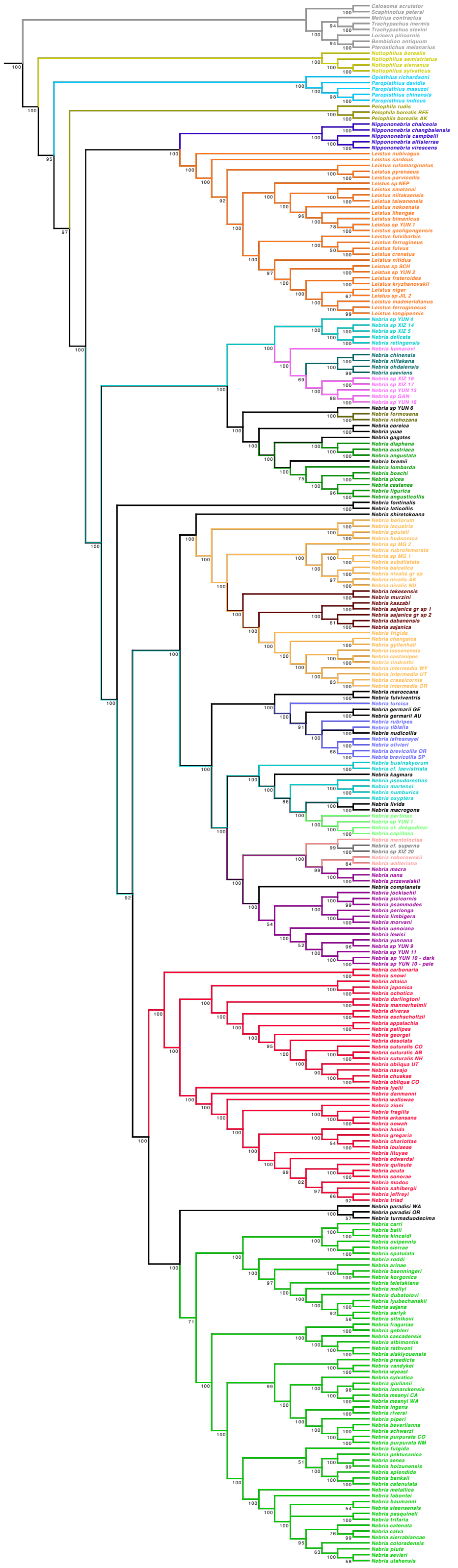

Figure S1. Majority rule consensus tree of 150,000 trees from Bayesian analysis of eight-gene concatenated matrix; numbers on branches indicate estimates of posterior probabilities, expressed as percentages.

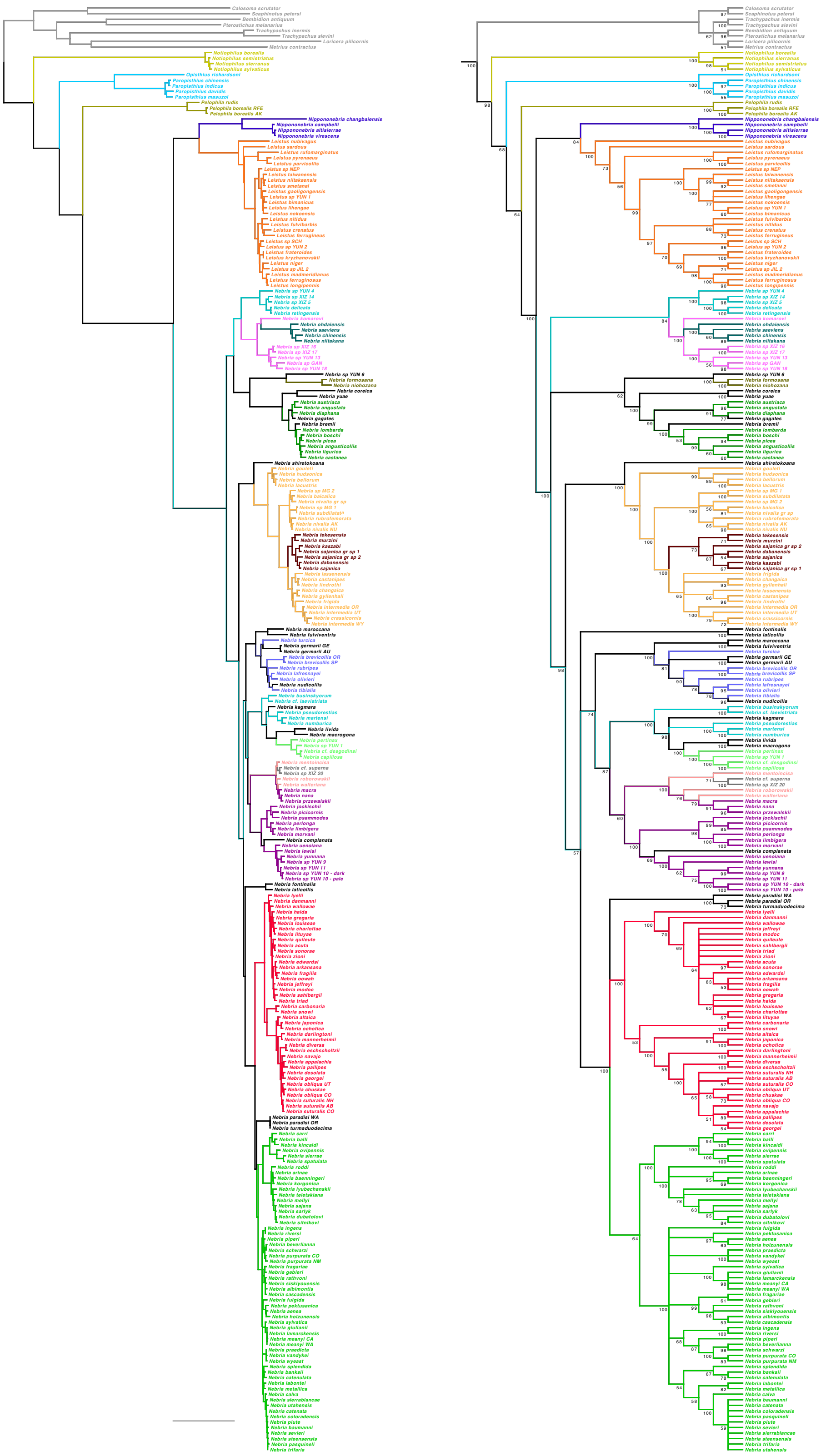

Figure S2. Maximum likelihood trees for concatenated matrix of all nuclear genes. Left: maximum likelihood tree; scale bar = 0.1 units, as estimated by RAxML. Right: majority rule consensus tree of trees from bootstrap replicates; numbers on branches indicate percentage of bootstrap replicates with that clade.

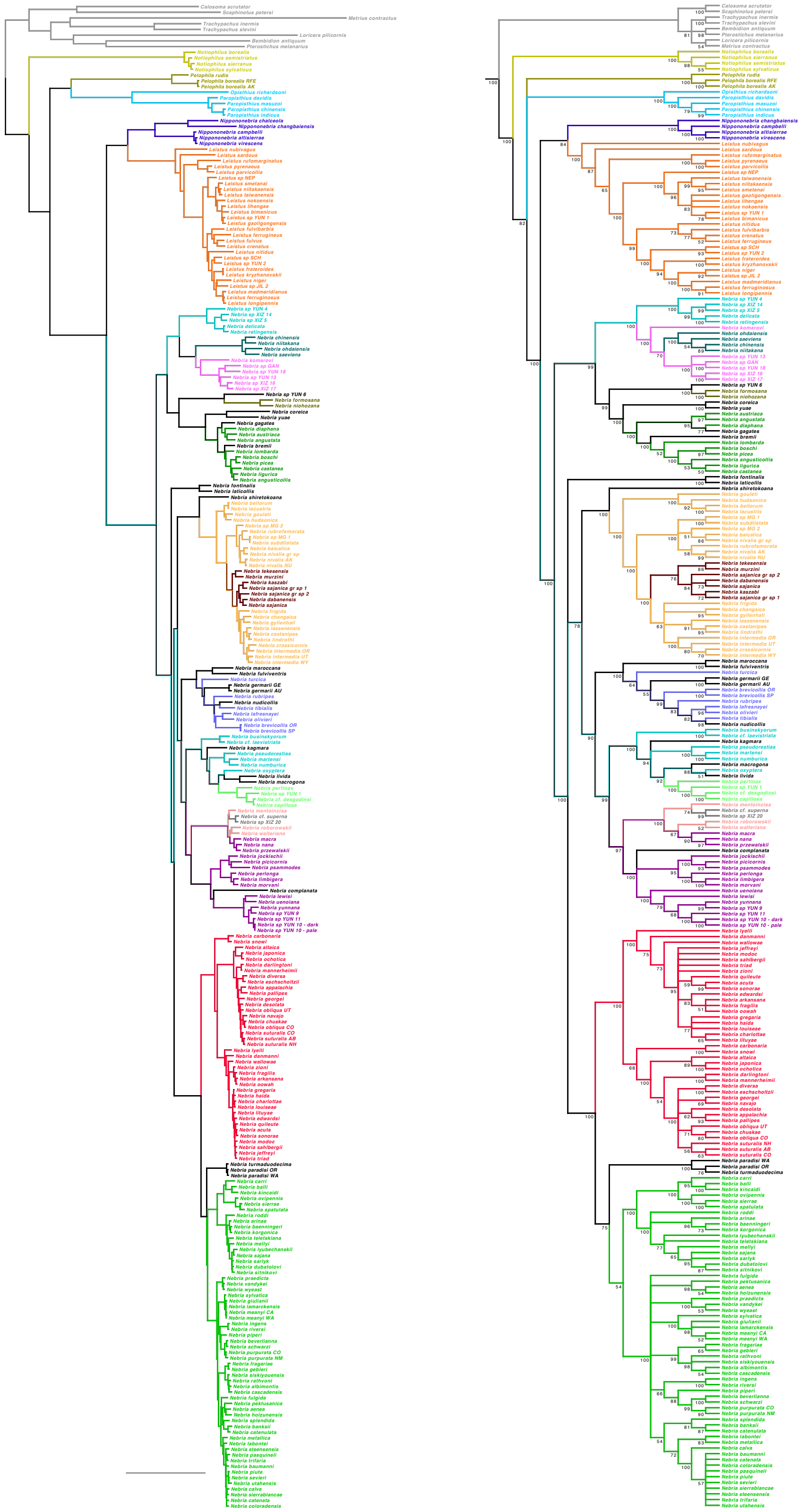

Figure S3. Maximum likelihood trees for concatenated matrix of all nuclear protein-coding genes. Left: maximum likelihood tree; scale bar = 0.1 units, as estimated by RAxML. Right: majority rule consensus tree of trees from bootstrap replicates; numbers on branches indicate percentage of bootstrap replicates with that clade.

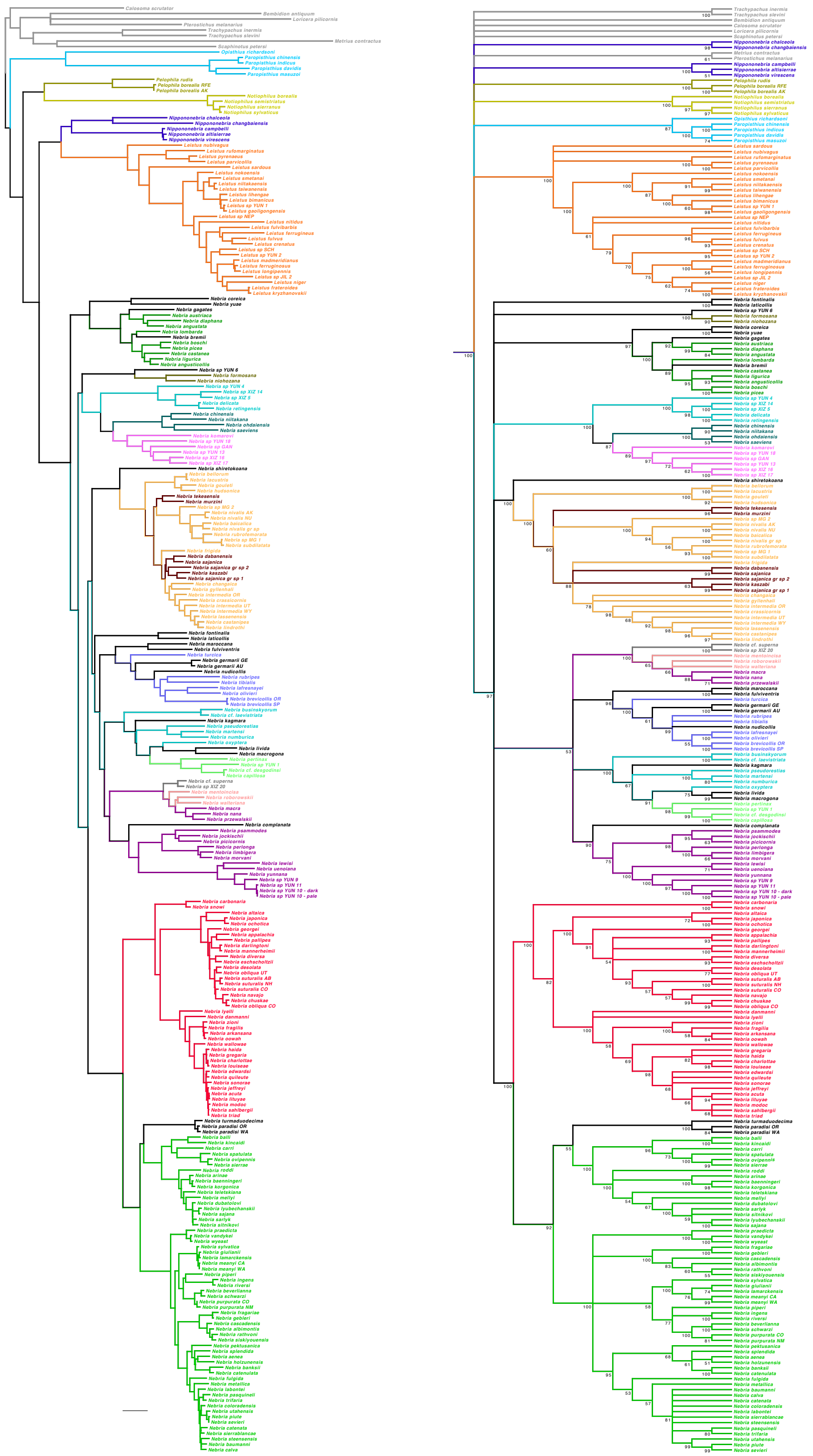

Figure S4. Maximum likelihood trees for concatenated matrix of all mitochondrial genes. Left: maximum likelihood tree; scale bar = 0.1 units, as estimated by RAxML. Right: majority rule consensus tree of trees from bootstrap replicates; numbers on branches indicate percentage of bootstrap replicates with that clade.

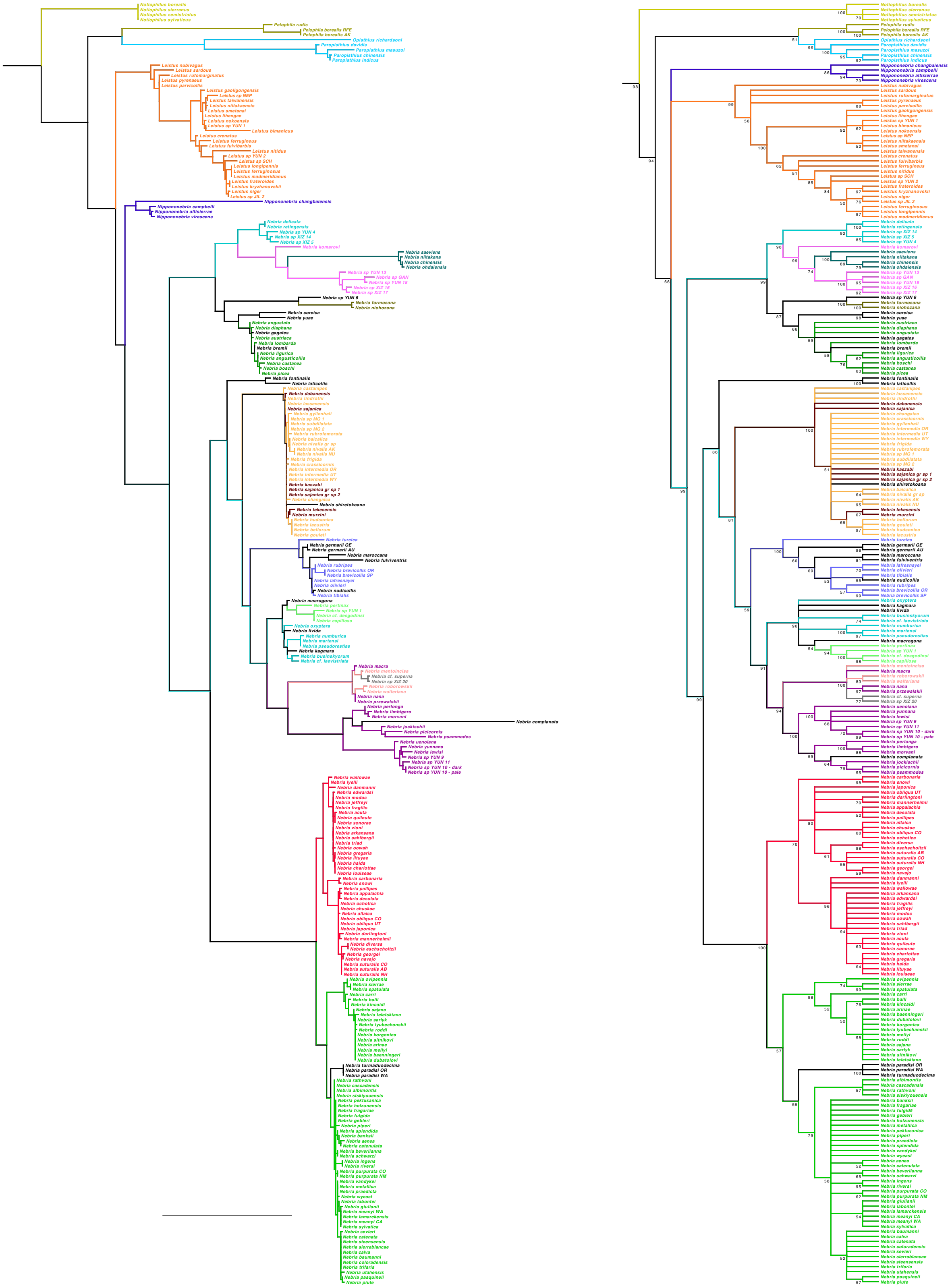

Figure S5. Maximum likelihood trees for 28S. Left: maximum likelihood tree; scale bar = 0.1 units, as estimated by RAxML. Right: majority rule consensus tree of trees from bootstrap replicates; numbers on branches indicate percentage of bootstrap replicates with that clade. Outgroups graphically omitted.

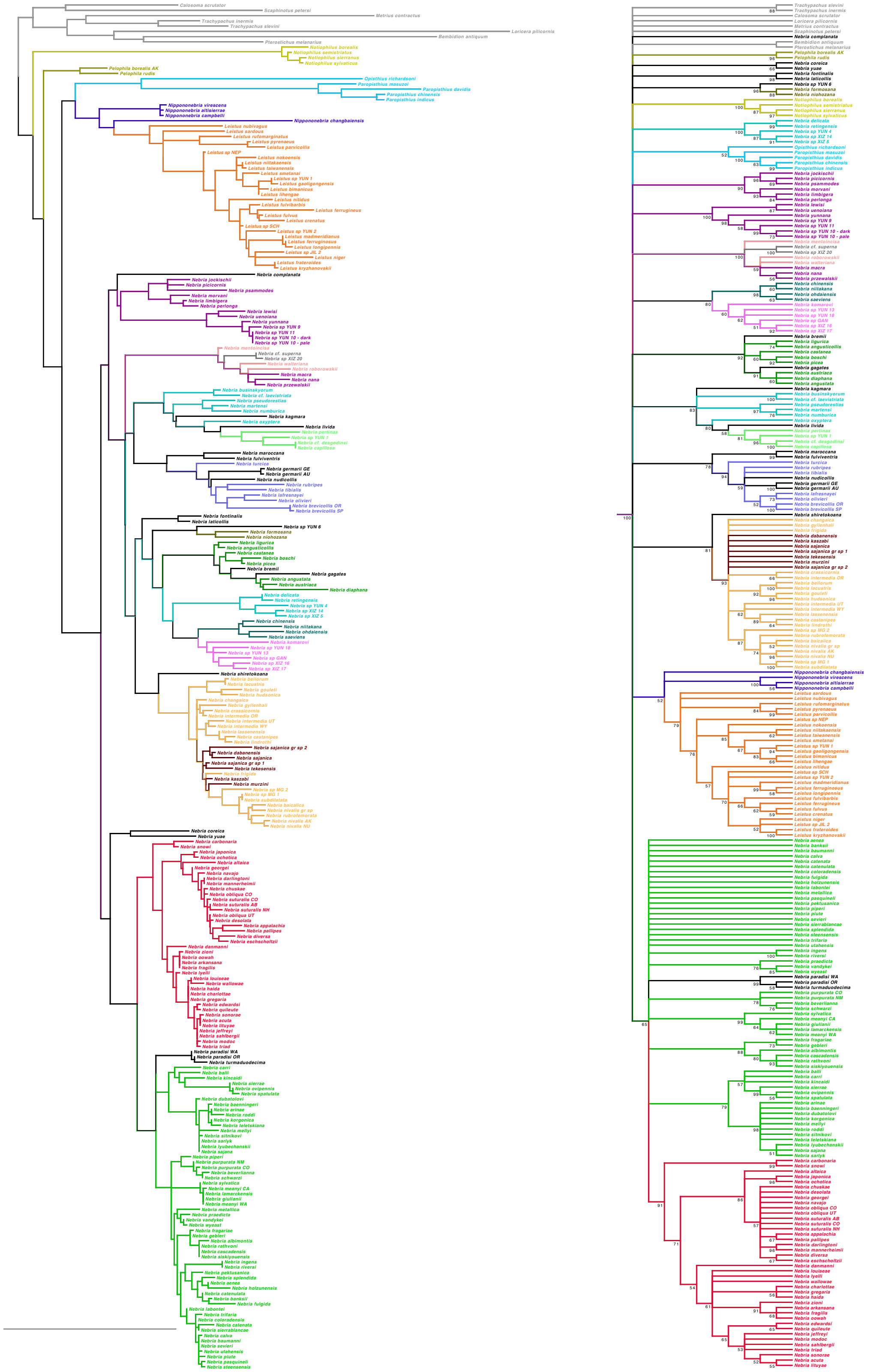

Figure S6. Maximum likelihood trees for 16S-ND1. Left: maximum likelihood tree; scale bar = 0.1 units, as estimated by RAxML. Right: majority rule consensus tree of trees from bootstrap replicates; numbers on branches indicate percentage of bootstrap replicates with that clade.

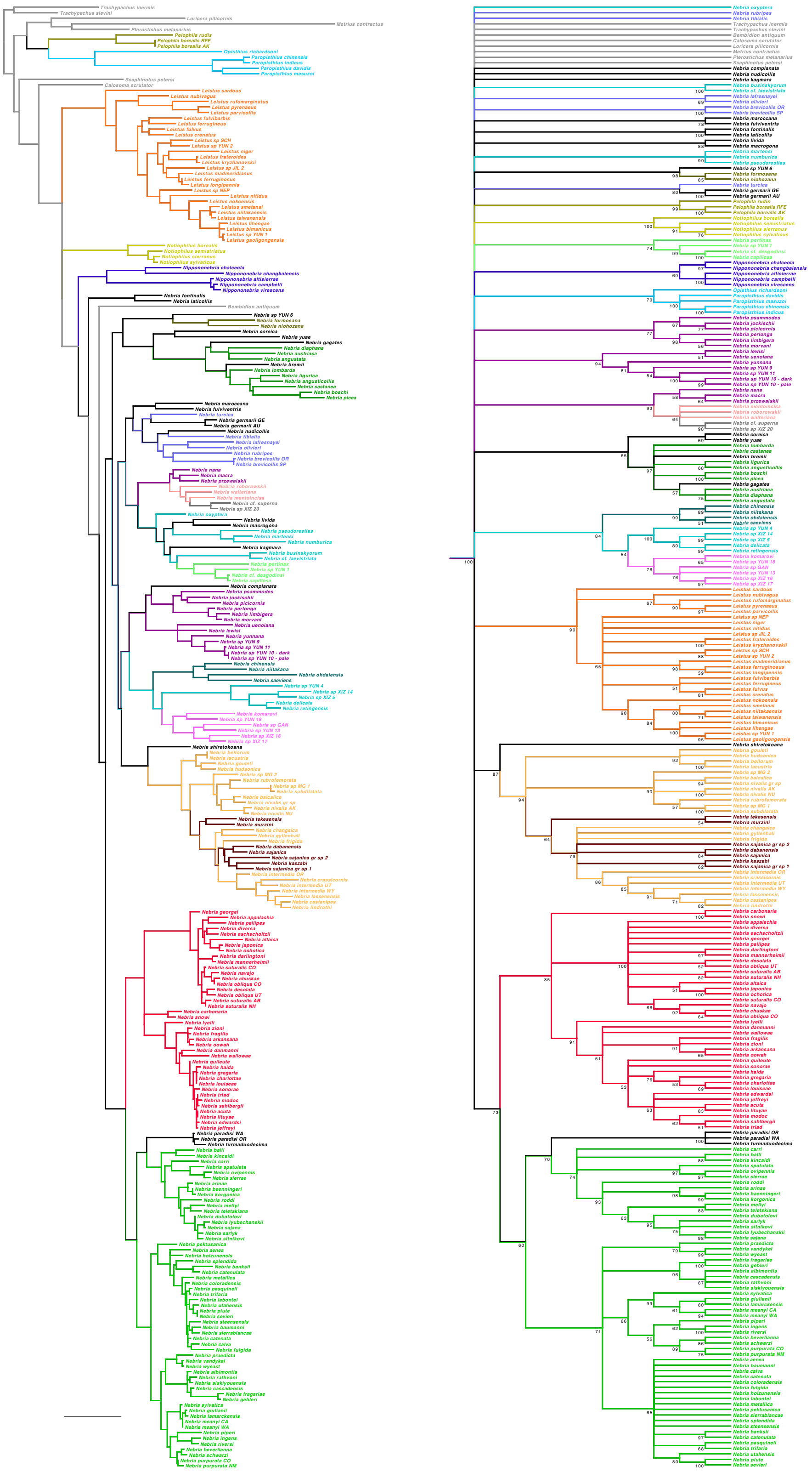

Figure S7. Maximum likelihood trees for the COI BC. Left: maximum likelihood tree; scale bar = 0.1 units, as estimated by RAxML. Right: majority rule consensus tree of trees from bootstrap replicates; numbers on branches indicate percentage of bootstrap replicates with that clade.

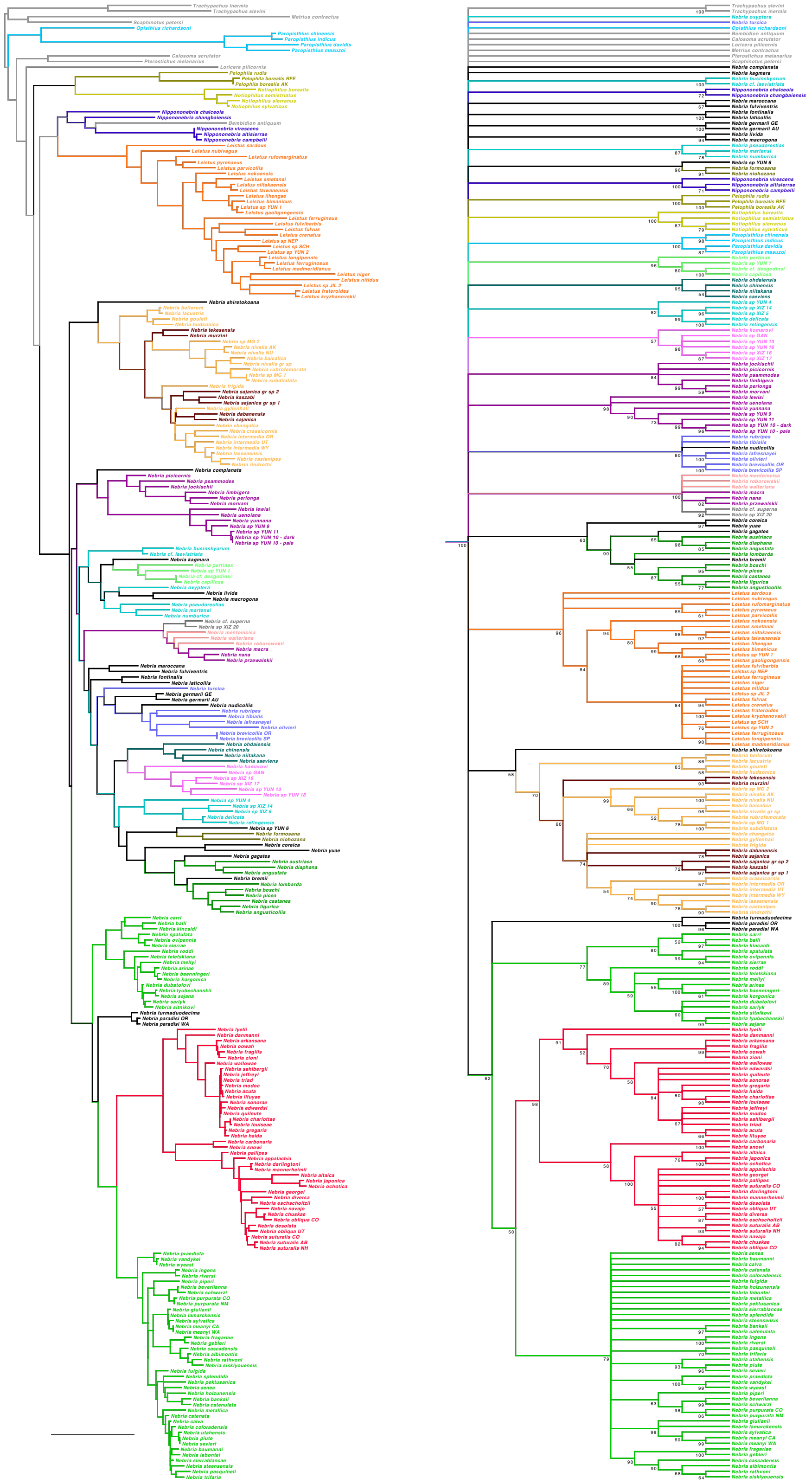

Figure S8. Maximum likelihood trees for the COI PJ. Left: maximum likelihood tree; scale bar = 0.1 units, as estimated by RAxML. Right: majority rule consensus tree of trees from bootstrap replicates; numbers on branches indicate percentage of bootstrap replicates with that clade.

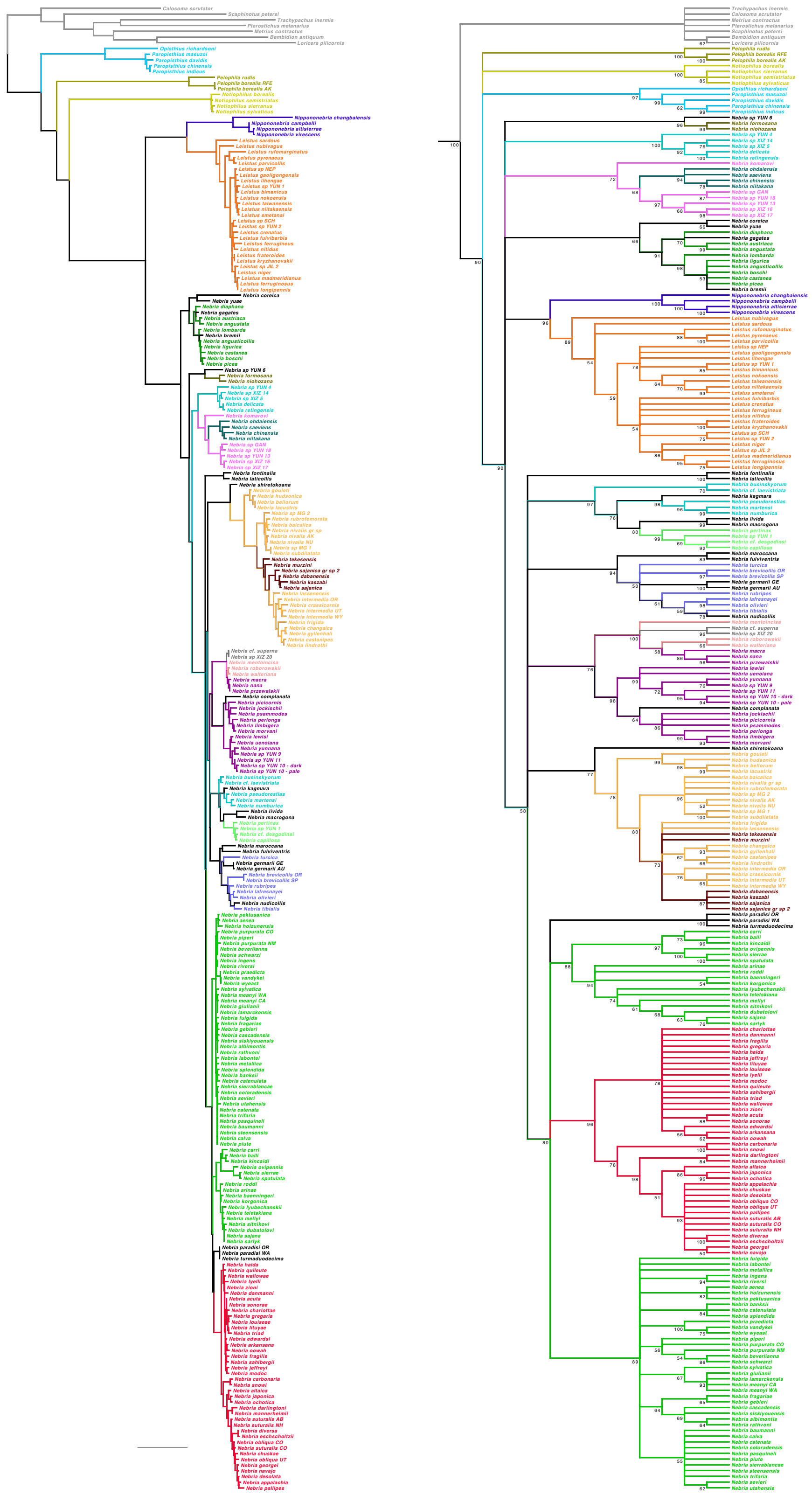

Figure S9. Maximum likelihood trees for the CAD2. Left: maximum likelihood tree; scale bar = 0.1 units, as estimated by RAxML. Right: majority rule consensus tree of trees from bootstrap replicates; numbers on branches indicate percentage of bootstrap replicates with that clade.

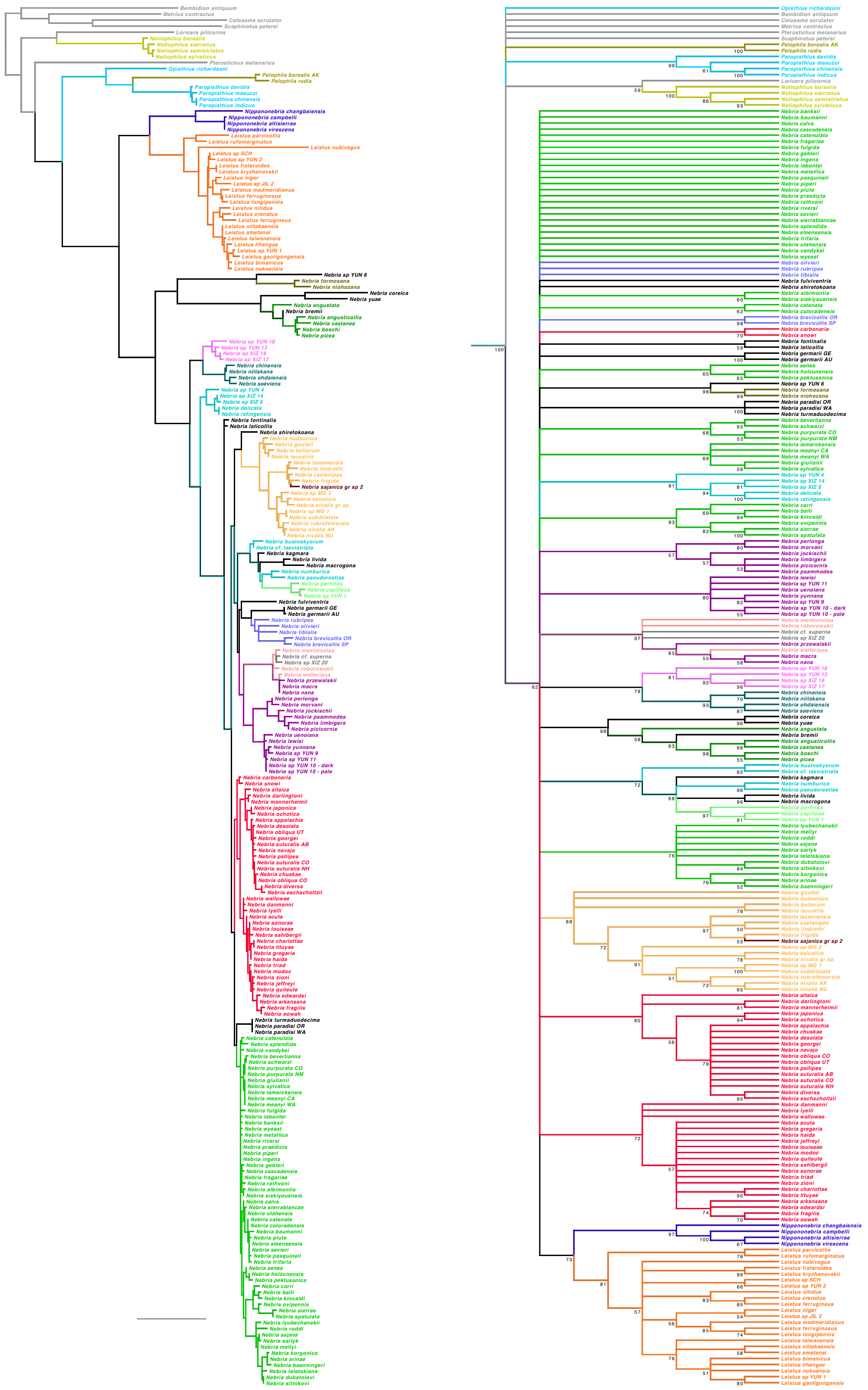

Figure S10. Maximum likelihood trees for the PEPCK. Left: maximum likelihood tree; scale bar = 0.1 units, as estimated by RAxML. Right: majority rule consensus tree of trees from bootstrap replicates; numbers on branches indicate percentage of bootstrap replicates with that clade.

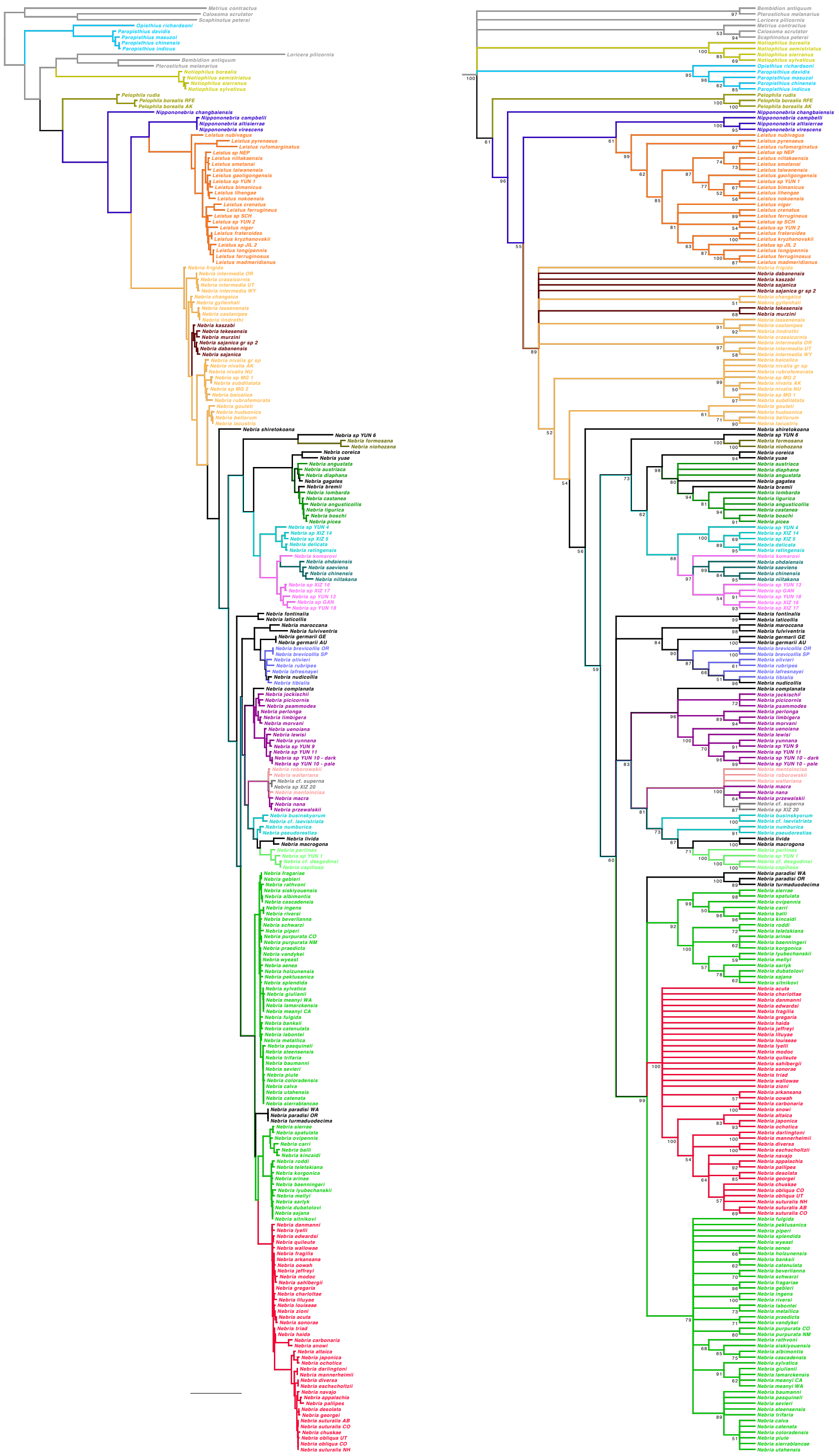

Figure S11. Maximum likelihood trees for the Topo. Left: maximum likelihood tree; scale bar = 0.1 units, as estimated by RAxML. Right: majority rule consensus tree of trees from bootstrap replicates; numbers on branches indicate percentage of bootstrap replicates with that clade.

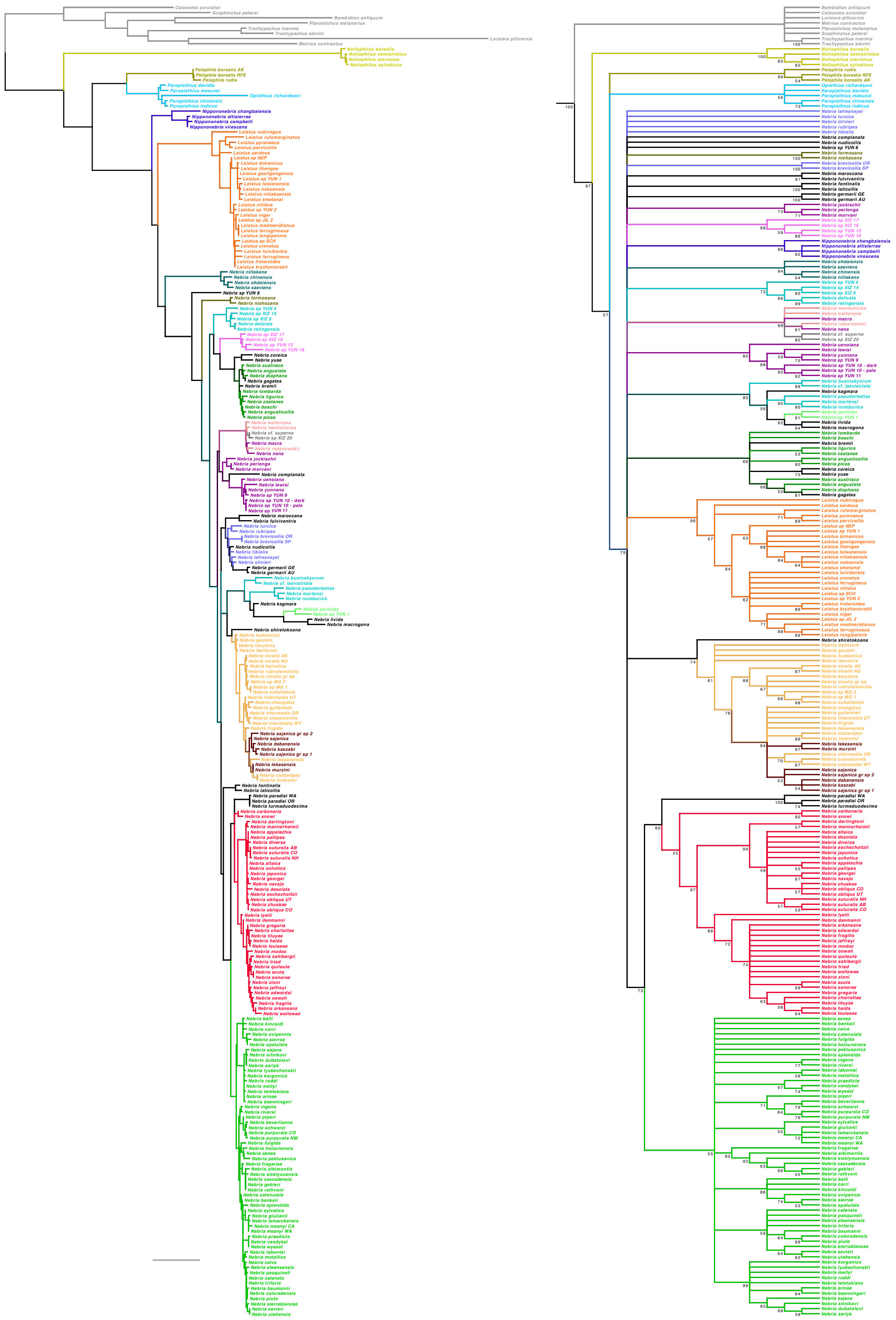

Figure S12. Maximum likelihood trees for the *wg*. Left: maximum likelihood tree; scale bar = 0.1 units, as estimated by RAxML. Right: majority rule consensus tree of trees from bootstrap replicates; numbers on branches indicate percentage of bootstrap replicates with that clade.

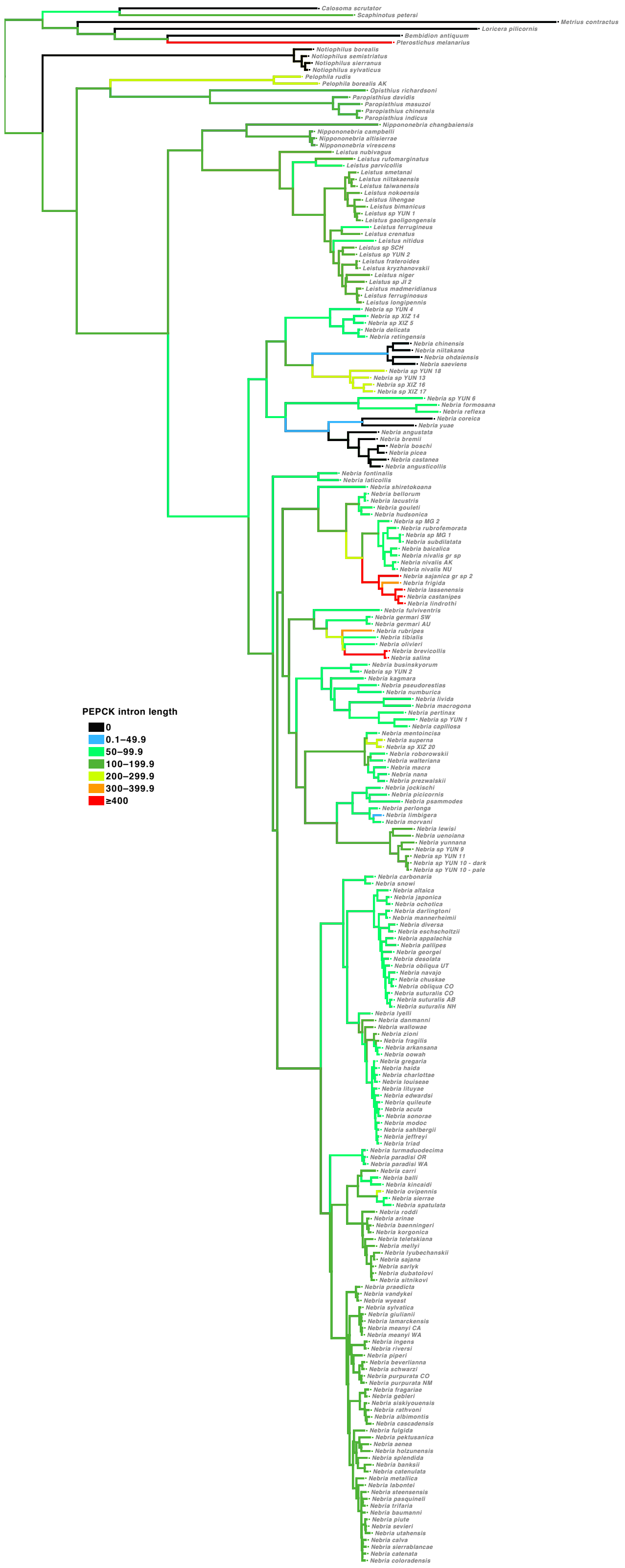

Figure S13. Maximum likelihood tree for concatenated matrix showing only those taxa for which PEPCK was sequenced, with branches colored according to the squared-change parsimony reconstruction of the length of the PEPCK intron.
